# Toward the Rational Design of Organic Catalysts for Organocatalysed Atom Transfer Radical Polymerisation

**DOI:** 10.3390/polym16030323

**Published:** 2024-01-24

**Authors:** Zhilei Wang, Chenyu Wu, Wenjian Liu

**Affiliations:** Institute of Frontier Chemistry, School of Chemistry and Chemical Engineering, Shandong University, Qingdao 266237, China; 201920282@mail.sdu.edu.cn

**Keywords:** O-ATRP, organic photocatalyst design, descriptors, property–performance relationships

## Abstract

Thanks to their diversity, organic photocatalysts (PCs) have been widely used in manufacturing polymeric products with well-defined molecular weights, block sequences, and architectures. Still, however, more universal property-performance relationships are needed to enable the rational design of such PCs. That is, a set of unique descriptors ought to be identified to represent key properties of the PCs relevant for polymerisation. Previously, the redox potentials of excited PCs (PC^*^) were used as a good descriptor for characterising very structurally similar PCs. However, it fails to elucidate PCs with diverse chromophore cores and ligands, among which those used for polymerisation are a good representative. As showcased by model systems of organocatalysed atom transfer radical polymerisation (O-ATRP), new universal descriptors accounting for additional factors, such as the binding and density overlap between the PC^*^ and initiator, are proposed and proved to be successful in elucidating the experimental performances of PCs in polymerisation. While O-ATRP is exemplified here, the approach adopted is general for studying other photocatalytic systems.

## 1. Introduction

It is well-known [1] that the macroscopic properties of polymers can be tuned by their molecular weight distribution [2,3], block sequences [4,5], and architectures [6,7]. This can be achieved in chain-growth polymerisation by controlling the growth of polymer chains through reversible activation and deactivation of the propagating species (referred to as controlled polymerisation). One popular strategy for controlling the chain growth is to incorporate photocatalysis, which enables temporal, spatial, and sequence control of the polymerisation process by external light regulation [8,9,10] so as to fabricate well-defined polymers and materialise 3D printing [11], surface patterning [12,13,14], and photo-flow processing [15,16]. This strategy has proven to be successful in a variety of controlled polymerisation systems that are distinguished by the end groups X of the polymerisation initiators R−X [17]. These systems are all mediated by a photocatalytic cycle composed of (i) photoexcitation, (ii) activation, and (iii) deactivation [17,18,19], specifically:

(i) PC→hvPC*1→ISCPC*3;

(iia) PC^*^ + R−X → PC^*^/R−X;

(iib) PC^*^/R−X → PC^·+^/X^−^ + R^·^;

(iic) R^·^ + *n*M → P_*n*_^·^;

(iii) PC^·+^/X^−^ + P_*n*_^·^ → PC + P_*n*_ − X.

**Figure 1:**
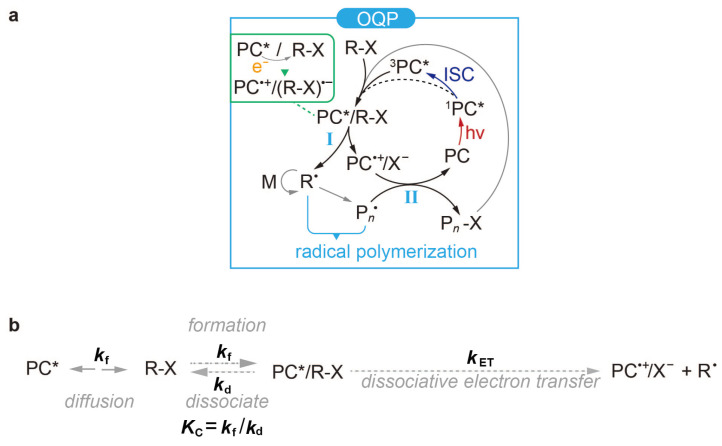
(**a**) Typical catalytic cycle for organocatalysed atom transfer radical polymerisation (O-ATRP) [19,23,24,25]. OQP: oxidative quenching pathway; I: activation; II: deactivation; ISC: intersystem crossing. (**b**) General processes of the activation step. $k_{f}$: rate constant for the formation of PC^*^/R−X through a diffusion-driven encounter between PC^*^ and R−X; $k_{d}$: rate constant for the dissociation of PC^*^/R−X; $K_{c}$: PC^*^/R−X formation/dissociation equilibrium constant for PC^*^ + R−X ↔ PC^*^/R−X; $k_{\mathrm{ET}}$: dissociative electron transfer rate constant.

In the photoexcitation step, a photocatalyst (PC) absorbs a photon to reach an excited-state PC (PC^*^). The activation step starts with the formation of an exciplex PC^*^/R−X, which then undergoes a chemical reaction to produce a reactive species R^·^ for the growth of a polymer P_*n*_^·^ with *n* monomer additions. Throughout the present work, the term ‘exciplex’ pertains to PC^*^/R−X and is used to signify its role as an encounter exciplex in the context of the electron transfer reaction. After this, the deactivation step restores all the intermediates to the ground state. The typical photocatalytic cycle is distinguished by the mechanism of the activation step known as the oxidative quenching pathway [17,18,20] (OQP, Figure 1a). The key process of the OQP can be called [21] dissociative electron transfer in the exciplex PC^*^/R−X (e.g., PC^*^/R−X → PC^·+^/X^−^ + R^·^), emphasising the concerted electron transfer (e.g., PC^*^/R−X → PC^·+^ + (R−X)^·−^) and the bond breaking of R−X (e.g., R−X → R^·^ + X^·^). Although transition metal-based (e.g., Cu, Au, Fe, Ir, and Ru) PCs were used in initial investigations [17,19], they were soon replaced by organic PCs to avoid the usually toxic metal residues in the polymer products [18,22]. As is well-known, organic systems can be facilely fabricated by virtue of widely accessible chemical groups. In particular, their simple geometric and electronic structures allow one to establish good structure–property–performance (SPP) relationships for the rational and precise design of desired organic PCs [17] by identifying a set of unique descriptors to represent key properties of the PCs relevant for polymerisation.

Investigations into SPP relationships have been instrumental in advancing the field of photo-controlled polymerisation, particularly in the development of new PCs over the past decade [9,17,18,19]. Despite these advancements, there are ongoing challenges. A key focus is the reduction of catalyst loading in organic PCs to ppm levels relative to the monomer, especially in the context of organocatalysed atom transfer radical polymerisation (O-ATRP) [26,27,28,29]. O-ATRP, while utilising commercially viable and affordable initiators suited for industrial applications, is highly dependent on the efficacy of PCs for precise polymerisation control. This makes minimising catalyst loading a complex task requiring rational strategies grounded in SPP relationships. The importance of this endeavour lies not only in reducing the cost of organic PCs to facilitate industrial application but also in decreasing residual PCs in the final polymer, which could otherwise compromise polymer quality [18].

Recent years have seen a surge in rational approaches guided by existing SPP relationships. These approaches have primarily focused on enhancing photon absorption, fostering the creation of long-lived states, and boosting excited-state redox capabilities [17,18]. Such concerted efforts have led to the development of a variety of effective organic PCs characterised by diverse chromophores and ligands. In the context of O-ATRP, certain organic PCs, particularly those with charge-transfer states as low-lying excited states, have shown promise in controlling O-ATRP with only ppm-level catalyst loading [27,28,30,31,32,33,34,35]. However, the mechanisms underlying the high efficiency of these charge-transfer PCs are not fully understood, which impedes a comprehensive understanding of the SPP relationships necessary for designing and optimising such ideal PCs. To address this gap, significant research efforts have been made. For instance, Damrauer and colleagues conducted in-depth kinetic studies of representative O-ATRP systems utilising the Rehm–Weller equation to analyse the characteristics of different PCs [35]. Although their work yielded valuable insights, it did not provide definitive conclusions. Nevertheless, since the Rehm–Weller equation includes practical factors relevant to PC efficiency, their findings offer an important basis for uncovering mechanistic details through advanced quantum chemical calculations.

In our study, we began with a theoretical analysis of the Rehm–Weller equation, identifying the binding strength within the excited encounter complex formed by the PC and the initiator R-X as a crucial, often overlooked factor for activation efficiency. This factor stands alongside the more commonly recognised electron transfer rate constant, offering a more comprehensive understanding of activation in O-ATRP. Building on this, we proposed four property descriptors encompassing the thermodynamics of the reaction as well as the stability of the encounter complex for both the activation and deactivation steps. We then rigorously tested these descriptors through high-level quantum chemical calculations and advanced wavefunction analysis methods using a classical O-ATRP system as a model. In particular, the recently developed localised orbital scaling correction (LOSC) [36,37] method was employed to address the delocalisation error in density functional theory (DFT) calculations. Additionally, our group’s newly developed fragment-to-molecule (F2M) [38,39] approach was applied to precisely delineate the electronic evolutions occurring during the activation process. By virtue of advanced approaches, our results not only confirmed the importance of the four descriptors but also solidified their physical foundation behind the O-ATRP mechanism. This bolstered our confidence in their practical application and validation within typical O-ATRP systems. Subsequently, these descriptors were used to evaluate a range of existing PCs for O-ATRP. Indeed, these descriptors demonstrated their efficacy in predicting performance outcomes in O-ATRP, especially in highlighting the exceptional efficiency of those organic PCs with ppm-level catalyst loading. On top of these insights, targeted recommendations are provided for developing more efficient PCs in O-ATRP.

## 2. Theoretical Background

Major performance aspects in controlled polymerisation include (i) initiator efficiency ($I^{*}$), (ii) molecular weight dispersity (*Đ*), and (iii) catalyst loading, among which $I^{*}$ describes what percentage of the initiators is effectively converted to polymer chains at the early stage of polymerisation. This can be calculated using the theoretical number-average molecular weight ($M_{n,\mathrm{theo}}$, g/mol) divided by the experimentally determined number-average molecular weight ($M_{n,\exp}$, g/mol), that is, $I^{*}$ = ($M_{n,\mathrm{theo}}$/$M_{n,\exp}$) × 100%, which is a percentage value [18]. An $I^{*}$ value lower than 100% indicates that some of the initiators either fail to activate or activate too late during polymerisation such that the converted polymers are too short to be detected. An $I^{*}$ value less than 100% might also indicate the early-stage degradation of some initiators during polymerisation. Generally, an $I^{*}$ close to 100% suggests efficient activation, where almost all initiators are transformed into polymer chains. However, an $I^{*}$ exceeding 100% could suggest the occurrence of side reactions that generate radicals, leading to auto-initiation. On the other hand, *Đ* describes the homogeneity of the polymer chain lengths. As such, $I^{*}\approx 100\%$ and as low a *Đ* as possible would mean virtually homogeneous polymer chains with predictable chain lengths [18,19]. The amount of PC necessary to achieve this is then called the catalyst loading (ppm, with respect to the monomer) [17,19]. According to the catalytic mechanisms shown in Figure 1a, the photoexcitation, activation, and deactivation steps may all affect these performance parameters and are hence analysed separately below.

The primary role of the photoexcitation step is to populate PC^*^ for the activation step, which can be classified into two types, with the first singlet excited state ($S_{1}$) of PC (^1^PC^*^) [40,41,42] or the first triplet excited state ($T_{1}$) of PC (^3^PC^*^) [27,30,32,43,44] as the major reaction species, as signified by a dominant singlet ($\Phi_{S}$) or triplet ($\Phi_{T}$) quantum yield [35] provided that ^1^PC^*^ and ^3^PC^*^ are both reactive. Note that, even if $\Phi_{T}$ is approximately the same as $\Phi_{S}$, ^3^PC^*^ can still be considered the major reactant [17] due to its longer lifetime $\tau_{T}$ than that ($\tau_{S}$) of ^1^PC^*^. Different PCs of the same category (^3^PC^*^ or ^1^PC^*^) can then be distinguished by their relative quantum yields and/or lifetimes. However, recent Stern–Volmer quenching experiments reveal [35] that $\Phi_{S/T}$ and $\tau_{S/T}$ are not characteristic factors for predicting the performances of different PCs. Instead, it is the activation step that provides the major information [35,41,42]. The impact of $\Phi_{S/T}$ and $\tau_{S/T}$ is significantly influenced by the catalyst loading. In particular, high $\Phi_{T}$ and long $\tau_{T}$ are the default for PC* to participate in the activation process effectively, especially when the catalyst loading is low, as this is one of the desired polymerisation performance aspects. As such, high $\Phi_{T}$ and long $\tau_{T}$ need to be guaranteed for the PC candidates before further analysis.

It is obvious that enhanced activation can improve the polymerisation performances [17,18] by facilitating the rapid activation of all the R−X (targeting $I^{*}\approx 100\%$), effective alternation in the growth of different chains (targeting *Đ* as low as possible), as well as the best utilisation of PCs (targeting catalyst loading as low as possible with respect to the monomer). The overall rate constant $k_{\mathrm{act}}$ ($M^{-1}$$s^{-1}$) of the activation step,

(1) $$k_{\mathrm{act}}=\frac{k_{f}K_{c}k_{\mathrm{ET}}}{K_{c}k_{\mathrm{ET}}+k_{f}},$$

is related [35,45] to three consecutive processes, viz., diffusion-driven formation of the exciplex PC^*^/R−X with a rate constant $k_{f}$ ($M^{-1}$$s^{-1}$), retention of the exciplex (i.e., ${\mathrm{PC}}^{*}+R-X\overset{k_{f}}{\underset{k_{d}}{⇌}}{\mathrm{PC}}^{*}/R-X$) with a formation/dissociation equilibrium constant $K_{c}$ ($M^{-1}$), and dissociative electron transfer in PC^*^/R−X with a rate constant $k_{\mathrm{ET}}$ ($s^{-1}$). $k_{f}$ can be estimated according to [35]

(2) $$k_{f}=\frac{8RT}{3\eta },$$

where η (N·s·$m^{-2}$) is the viscosity of the solvent, *R* (J·${\mathrm{mol}}^{-1}$·$K^{-1}$) is the ideal gas constant, and *T* (K) is the temperature. As such, $k_{f}$ can be considered a constant for a given solvent at room temperature. $K_{c}$ is defined by

(3) $$K_{c}=\frac{k_{f}}{k_{d}},$$

where $k_{d}$ ($s^{-1}$) is the dissociation rate constant of the exciplex PC^*^/R−X. Clearly, a higher $K_{c}$ means that PC^*^ and R−X in PC^*^/R−X are more strongly bound together in resistance to dissociation. By far, $K_{c}$ is usually estimated as a small constant under the assumption that the binding strength between PC^*^ and R−X is negligible [35]. If the variations of $k_{f}$ and $K_{c}$ can indeed be ignored, it can be deduced from Equation (1) that $k_{\mathrm{ET}}$ is the determining factor for $k_{\mathrm{act}}$. According to the Marcus model [21,46,47], $k_{\mathrm{ET}}$ reads

(4) $$\begin{array}{cc} k_{\mathrm{ET}} & =\frac{k_{B}T}{h}\exp\left[-\frac{\Delta G^{‡}}{k_{B}T}\right]\\ \; & \approx \frac{k_{B}T}{h}\exp\left[-\frac{\Delta E^{‡}}{k_{B}T}\right], \end{array}$$

where $k_{B}$ (J/K) is the Boltzmann constant, *h* (J·s) is the Planck constant, and $\Delta E^{‡}$ (J) is the electronic part of the Gibbs free energy barrier $\Delta G^{‡}$ (J). Under the displaced harmonic oscillator approximation, $\Delta G^{‡}$ (eV) can be calculated as

(5) $$\Delta G^{‡}=\frac{{\left(\Delta G_{0}+\lambda \right)}^{2}}{4\lambda },$$

where $\Delta G_{0}$ (eV) is the Gibbs free energy change of the aforementioned electron transfer and λ (eV) is the sum of the solvent reorganisation energy and the energy required to break the R−X bond [21]. Taking OQP as an example (Figure 1a), the electron transfer from PC^*^ to R−X can be decomposed to PC^*^ − $e^{-}$ → PC^·+^ and R−X + $e^{-}$ → (R−X)^·−^ such that $\Delta G_{0}$ can accordingly be written as [35]

(6) $$\Delta G_{0}=eE^{0}\left({\mathrm{PC}}^{\cdot +}/{\mathrm{PC}}^{*}\right)-eE^{0}\left(R-X/{\left(R-X\right)}^{.-}\right)-w_{C},$$

where $E^{0}$(PC^·+^/PC^*^) (eV) is the oxidation potential of PC^*^, $E^{0}$(R−X/(R−X)^·−^) (eV) is the reduction potential of R−X, *e* is the fundamental charge, and $w_{C}$ (eV) is a Coulombic work term associated with the electrostatic interaction between PC^·+^ and (R−X)^·−^ after electron transfer. It is hence clear that the performance of different PCs for the same initiator R−X can in this case be characterised by $E^{0}$(PC^·+^/PC^*^). Such redox potentials have been employed [17,18] successfully to elucidate PCs with very similar chemical and/or geometric structures, where similar binding strengths and density overlaps between PC^*^ and R−X can be expected. However, the situation is different for the majority of PCs developed for polymerisation, where a great variety of different chromophore cores and substituents are used [17]. In this case, neglecting the binding strength between PC^*^ and R−X will overestimate the dissociation rate $k_{d}$, thereby underestimating $K_{c}$ (cf. Equation (3)). To see how $K_{c}$ affects $k_{\mathrm{act}}$, we look at the change of $k_{\mathrm{act}}$ over that of $K_{c}$,

(7) $$\frac{dk_{\mathrm{act}}}{dK_{c}}=\frac{k_{f}^{2}k_{\mathrm{ET}}}{{\left(k_{\mathrm{ET}}K_{c}+k_{f}\right)}^{2}}>0$$

It can be seen that $k_{\mathrm{act}}$ is positively correlated with $K_{c}$ and becomes insensitive to $K_{c}$ only when $k_{\mathrm{ET}}$ is large enough ($k_{f}$ in the denominator can be neglected when $k_{\mathrm{ET}}$ approaches infinity, but this condition rarely happens), viz.,

(8) $$\frac{dk_{\mathrm{act}}}{dK_{c}}∼\frac{k_{f}^{2}}{k_{\mathrm{ET}}K_{c}^{2}}∼0$$

A direct deduction is that taking $K_{c}$ as a small constant will underestimate $k_{\mathrm{act}}$ in the case of low $k_{\mathrm{ET}}$; such an underestimation may amount to 4 to 5 times, as shown by a recent experimental study [35] of O-ATRP [18,19,26,48]. Close inspections reveal that the adopted aryl-substituted phenoxazine [35] type of PCs feature charge-transfer characters in their lowest excited states PC^*^ (i.e., PC^*^ exhibits charge separation), which is very common in such PCs [30,32,43]. Furthermore, it has been established that charge-transfer excited states, known for enhancing $\Phi_{T}$ in many cases [30,31,32], also lead to charge separation that enhances the binding strength $\Delta E_{b}^{\mathrm{ex}}$ with polar R-X (as discussed below). This helps to elucidate recent observations indicating that charge-transfer states can enhance O-ATRP performance, even in cases where $\Phi_{T}$ is not substantially improved [18]. Since charge-separated PC^*^ and R−X are both polar systems, it is expected that there exists appreciable binding in between, thereby leading to enhanced $K_{c}$ (cf. Equation (3)) and hence $k_{\mathrm{act}}$ (cf. Equation (7)). Through analysis of the Rehm–Weller equation, it is clear that a higher binding strength $\Delta E_{b}^{\mathrm{ex}}$ and longer lifetime of the encounter exciplex PC^*^/R−X contribute positively to the activation kinetics $k_{\mathrm{act}}$. The importance of the encounter complex lifetime has also been reported in electron transfer between proteins [49]. As such, the binding strength $\Delta E_{b}^{\mathrm{ex}}$ between PC^*^ and R−X is likely a key factor to be used as a descriptor. On the other hand, the density overlap between PC^*^ and R−X has been neglected when deriving Equation (6) for OQP in O-ATRP, which is doomed to fail when PC^*^ and R−X are close to each other in space. In such situations, the energy barrier $\Delta E^{‡}$ in $k_{\mathrm{ET}}$ (Equation (4)) should be directly calculated to evaluate the efficiency of dissociative electron transfer in O-ATRP instead of being estimated by virtue of the redox potentials (cf. Equations (5) and (6)).

Lastly, the deactivation step PC^·+^/X^−^ + P_*n*_^·^ → PC + P_*n*_−X in O-ATRP completes the catalytic cycle, where the ion pair PC^·+^/X^−^, produced from PC^*^/R−X → PC^·+^/X^−^ + R^·^ in the activation step [50,51,52], is the key species that can reversibly deactivates P*n*^·^ to generate the ground-state PC and P*n*−X for the next cycle. It is thus desirable to prevent PC^·+^/X^−^ from dissociating to PC^·+^ and X^−^ since the dissociated PC^·+^ can break the catalytic cycle by reacting with either the solvent molecules [51] or P*n*^·^ [34] (vide infra). To avoid such side reactions, the binding strength $\Delta E_{b}^{\mathrm{PC-X}}$ between PC^·+^ and X^−^ must be sufficiently high. Moreover, similar to the activation step, the deactivation energy barrier $\Delta E_{\mathrm{de}}^{‡}$ can serve as a descriptor to assess the efficiency of the deactivation step in O-ATRP instead of being estimated by virtue of the redox potentials (cf. Equations (5) and (6)). In short, the binding strength $\Delta E_{b}^{\mathrm{ex}}$ between PC^*^ and R−X and the energy barrier $\Delta E^{‡}$ for PC^*^/R−X → PC^·+^/X^−^ + R^·^ in the activation step, the binding strength $\Delta E_{b}^{\mathrm{PC-X}}$ between PC^·+^ and X^−^ in the deactivation step, as well as the deactivation energy barrier $\Delta E_{\mathrm{de}}^{‡}$ in the deactivation step can be used as four descriptors.

These property descriptors can subsequently be correlated with the performance of O-ATRP. This correlation is referred to as the property–performance relationship. In this study, the four property descriptors can be easily determined through quantum chemical calculations. Subsequently, they are validated by testing against reported experimental O-ATRP performances. Table 1 presents the calculated property descriptors alongside the experimental performance parameters for comparison. In the concluding section of this manuscript, more delicate correlations are established by analysing their physical relationships.

## 3. Methods

### 3.1. Reaction Path Analysis

Standard theoretical calculations were performed [54,55,56,57] using DFT with the PBE0 functional [58] and def2-SVP basis set [59] coupled with the D3BJ dispersion correction [60] and a density-based implicit solvation model (SMD) [61] featuring dimethyl formamide (DMF) as the solvent. To mitigate the delocalisation error typically present in approximate density functionals, which can impede accurate energy barrier analysis, the LOSC scheme [36,37] was applied. This correction was specifically used to adjust the single-point energies at each geometry.

Taking the model system (vide infra) used in the present work as an example, for each geometry along the PC^*^/R−X reaction path obtained by the intrinsic reaction coordinate (IRC) method [62], the UPBE0-D3BJ/def2-SVP/SMD-DMF energy of PC^*^/R−X is further corrected by LOSC at UPBE0/def2-SVP so as to obtain the LOSC-corrected energy profile and energy barrier $\Delta E^{‡}$. The LOSC-corrected molecular orbitals at each geometry are then used for analysis of the electron and hole transfer by virtue of the ground-state fragment-localised molecular orbitals (FLMOs) constructed under the F2M scheme [38,39] at that geometry.

### 3.2. Determination of Descriptors

The binding strengths $\Delta E_{b}^{\mathrm{ex}}$ of ^1,3^PC^*^/R−X and $\Delta E_{b}^{\mathrm{PC-X}}$ of PC^·+^/X^−^ are determined by taking the electronic energy changes *E*(^1,3^PC^*^) + *E*(R−X)−*E*(^1,3^PC^*^/R−X) and *E*(PC^·+^) + *E*(X^−^)−*E*(PC^·+^/X^−^), respectively. The $\Delta E^{‡}$ for dissociative electron transfer is determined by the highest energy of the ^1,3^PC^*^/R−X reaction path subtracted by the energy of equilibrium ^1,3^PC^*^/R−X. In cases where the ^1,3^PC^*^/R−X IRC cannot be obtained readily, a relaxed scan starting from the equilibrium ^1,3^PC^*^/R−X, which increases the distance between alkyl-C of R−X, is performed to obtain the reaction path. Such an approach also applies to the energy barrier $\Delta E_{\mathrm{de}}^{‡}$ for the deactivation reaction.

### 3.3. Visualisation

The electrostatic potential (ESP) maps of ^3^PC^*^ and ^1^PC^*^ on the van der Waals surfaces at their respective equilibrium geometries were obtained by analysing wavefunctions calculated at UPBE0/def2-SVP/SMD-DMF and TDDFT/PBE0/def2-SVP/SMD-DMF, respectively. The ESP maps were visualised using VMD v1.9.4 [63] in conjunction with Multiwfn v3.8 [64].

## 4. Results and Discussion

### 4.1. Model Systems

**Figure 2:**
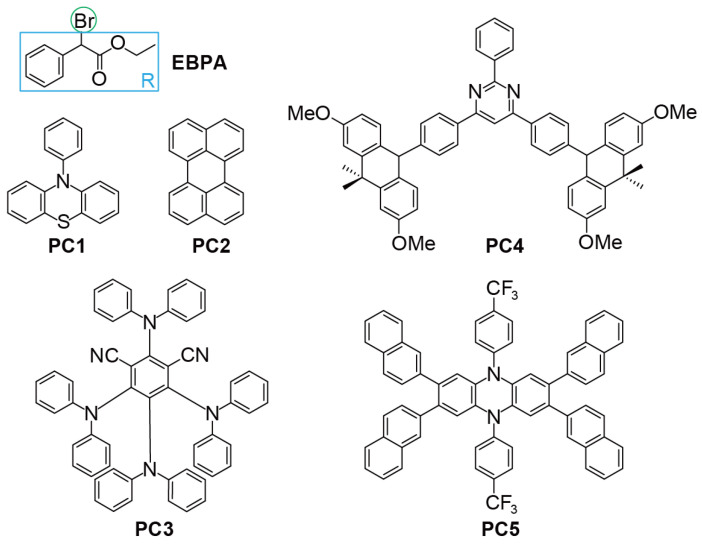
Chemical structures of the initiator (EBPA) and catalysts (PC1–5) in this study.

To examine the viability of the aforementioned four descriptors, we consider five model O-ATRP systems with EBPA (ethyl-α-bromophenylacetate) as the initiator and PC1-5 as the catalyst (see Figure 2). EBPA is one of the most commonly used initiators in O-ATRP [18,19], which takes the form of R−X, with R being an ethyl phenylacetate group and X being a Br atom. PC1-5 bears very different chromophore cores and substituents (cf. Figure 2), as well as distinct characters of PC^*^ (locally excited PC^*^ for PC1-2 [48,52] but charge-transfer PC^*^ for PC3-5 [27,28,53]). In accordance with such geometric and electronic differences, these PCs perform very differently in O-ATRP. As can be seen from Table 1, both PC1 [26,52] and PC2 [48], being the first O-ATRP PCs though, require rather high catalyst loading (≥1000 ppm) and are hence scarcely used nowadays. In contrast, PC3 [27] (No. 3a), PC4 [53], and PC5 [28] (No. 5a) can achieve acceptable polymerisation control ($I^{*}=61\%$ and *Đ* = 1.30, $I^{*}=91\%$ and *Đ* = 1.29, and $I^{*}=92\%$ and *Đ* = 1.27, respectively) at very low catalyst loading (10, 50, and 10 ppm, respectively). It can be seen from Table 2 that, among the photoexcitation properties (molar absorptivity $\epsilon_{\max}$ (L/mol·cm), quantum yields $\Phi_{S/T}$, and lifetimes $\tau_{S/T}$ (ns), only $\tau_{T}$ is very different between PC1 and PC4. However, the magnitude of $\tau_{T}$ (cf. Table 2) is just opposite the performances of PC1 and PC4 in O-ATRP (cf. Table 1), which is in line with the previous results that the photoexcitation properties of the PCs are not well-correlated with their performances [35,40,41,42].

To see why PC1 and PC4 are so different, the oxidation potentials $E^{0}$(PC^·+^/^1,3^PC^*^) (eV) of their ^1,3^PC^*^ were first examined (see Table 3). Unfortunately, the experimental results [27,28,48,52,53] were measured in different solvents, which prevents a direct comparison of the PCs. Moreover, such solvents were not those [e.g., N,N-dimethylformamide (DMF) and N,N-dimethylacetamide (DMAc)] that are commonly employed in O-ATRP. To first check the accuracy of the density functionals, the same solvents as used in the measurements of $E^{0}$(PC^·+^/^1,3^PC^*^) were employed in the TD-DFT calculations. As can be seen from Table S1 in the Supplementary Information (SI), among the five density functionals, PBE0 with D3BJ for dispersion correction, in conjunction with the def2-SVP basis set and SMD, performs the best on average (0.19 eV in error as compared to the experimental values of $E^{0}$(PC^·+^/^1,3^PC^*^); cf. Table 3). It can also be seen from Table 3 that there exist significant differences between DMF and those solvents employed merely for the purpose of measuring $E^{0}$(PC^·+^/^1,3^PC^*^). Therefore, it is more meaningful to use DMF (or similarly DMAc; cf. Table S2 in SI) when discussing the performances of PCs in O-ATRP. The so-calculated $E^{0}$(PC^·+^/^1,3^PC^*^), −2.91 (−2.31) and −2.22 (−2.21) eV for the ^1^PC^*^ (^3^PC^*^) of PC1 and PC4, respectively, indicate that ^1,3^PC^*^ of PC1 is more reducing than that of PC4, again opposite their performances. As such, the oxidation potentials cannot be taken as a molecular descriptor in this case. In passing, it is worth noting that the stabilities of PC^*^ and PC^·+^ can also play a significant role in O-ATRP performance, as seen in the alkyl core substitution (AkCS) effect observed for dihydrophenazine derivatives [18,28,34]. However, given that this study is primarily oriented towards identifying generally applicable property descriptors, we do not delve into factors specific to particular PC families, such as the stabilities of PC^*^ and PC^·+^ determined by their distinct chemical structures. Instead, we should switch to descriptors $\Delta E_{b}^{\mathrm{ex}}$, $\Delta E^{‡}$, $\Delta E_{b}^{\mathrm{PC-X}}$, and $\Delta E_{\mathrm{de}}^{‡}$ as discussed above. Before proceeding, it should be mentioned that $E^{0}$(PC^·+^/^1^PC^*^) of PC2 is higher than its $E^{0}$(PC^·+^/^3^PC^*^) by more than 1 eV, much larger than the corresponding differences of other PCs. It turns out that the highest occupied (HOMO; $\psi_{H}$) and lowest unoccupied (LUMO; $\psi_{L}$) molecular orbitals of PC2 are very close to each other in space, thereby resulting in a substantial repulsive interaction $2(\psi_{L}\psi_{H}|\psi_{L}\psi_{H})$ in ^1^PC^*^, which is not present in ^3^PC^*^ in view of the response kernel of TD-DFT (for more details, see Section S2 in the SI). In contrast, $2(\psi_{L}\psi_{H}|\psi_{L}\psi_{H})$ is much smaller for other PCs.

In addition to the above model systems with very different structures, we also examined another six catalysts with similar structures within the same family. Given that this study primarily focuses on identifying universally applicable property descriptors, we have included this part of the research in the SI (for more details, see Section S8).

### 4.2. Formation of Encounter Exciplex

The activation step starts with the formation of an exciplex ^1,3^PC^*^/R−X. In view of the quantum yields (see Table 2), ^1^PC^*^/R−X is the dominant species for PC2, but ^3^PC^*^/R−X is the dominant species for PC1, PC3, and PC4. As for PC5, ^3^PC^*^/R−X should also be predominantly populated according to the present calculations: An efficient $S_{1}$→$T_{2}$ intersystem crossing can occur at the minimum energy crossing point (MECP, at which the $S_{1}$/$T_{2}$ spin-orbit coupling [69] matrix element is 0.69 ${\mathrm{cm}}^{-1}$; see Supplementary Appendix B in the SI) by overcoming a 3.5 kcal/mol energy gap at $S_{1}$ from the $S_{1}$ equilibrium to the MECP.

**Figure 3:**
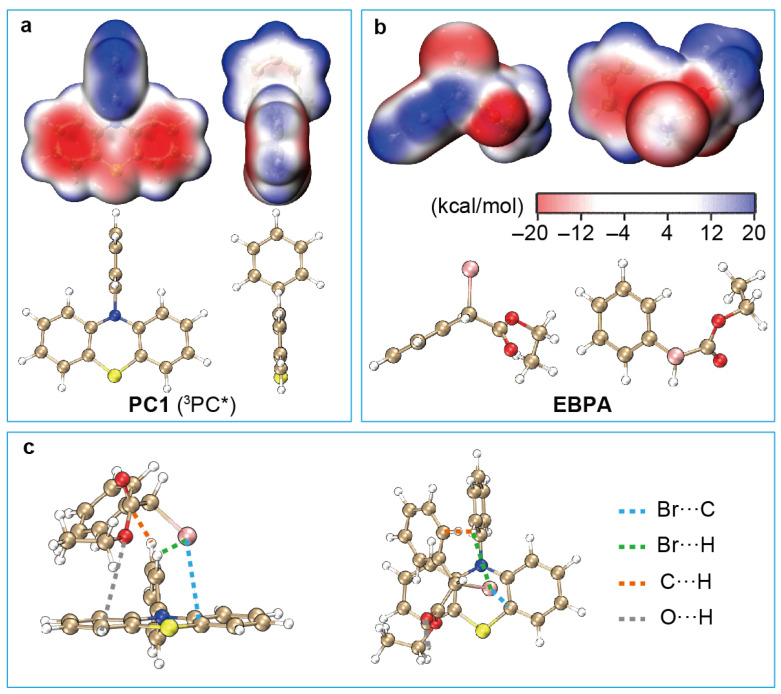
ESPs on the molecular surfaces of the $T_{1}$-state PC1 (**a**) and the ground-state EBPA (**b**) at UPBE0/def2-SVP/SMD-DMF. (**c**) Geometry of the exciplex ^3^PC^*^/R−X with PC1 as the catalyst and EBPA as the initiator optimised [57] with UPBE0-D3BJ/def2-SVP/SMD-DMF.

The equilibrium geometries of ^1,3^PC^*^ and ^1,3^PC^*^/R−X can, in principle, be optimised with the TDDFT analytic energy gradients [70]. However, to simplify the computation on one hand and facilitate subsequent analysis on the other, the unrestricted (U) PBE0-D3BJ/def2-SVP/SMD-DMF is used hereafter for ^3^PC^*^ and ^3^PC^*^/R−X. This option is further supported by the fact that UPBE0 and TDDFT/PBE0 yield very similar oxidation potentials for ^3^PC^*^ (see Tables S1 and S3 in the SI). In addition, we take a close look at the charge distributions in the case of PC1. It can be seen from Figure 3a that the ^3^PC^*^ of PC1 has some positive charges localised on the H atoms and some negative charges within the six-member C ring of the N-phenyl group. The two six-member C rings and the S atom in the phenothiazine group are also negatively charged. On the other hand, R−X has a phenyl group with positive charges on the H atoms and negative charges within the six-member C ring, as well as a negatively charged Br atom with a slightly positive outer region (Figure 3b). It is hence clear that ^3^PC^*^ and R−X can bind together by an electrostatic interaction within ^3^PC^*^/R−X, as shown in Figure 3c. The overall interaction between ^3^PC^*^ and R−X results in a binding strength $\Delta E_{b}^{\mathrm{ex}}$ of 11.2 kcal/mol, meaning that the dissociation of ^3^PC^*^/R−X to ^3^PC^*^ and R−X requires an energy of at least 11.2 kcal/mol in the case of PC1. The situation is very similar for the ^1^PC^*^/R−X of PC2. In contrast, the ^3^PC^*^/R−X of PC3, PC4, and PC5 have much higher binding strengths (15.0, 16.3, and 17.5 kcal/mol, respectively; cf. Table 1). This stems from the fact that the ^3^PC^*^ of PC3-5 have enhanced charge separations due to the charge transfer type of excitations [27,28,48,52,53] (see Figures S1 and S2 in the SI), thereby leading to stronger interactions with R−X.

### 4.3. Dissociative Electron Transfer

**Figure 4:**
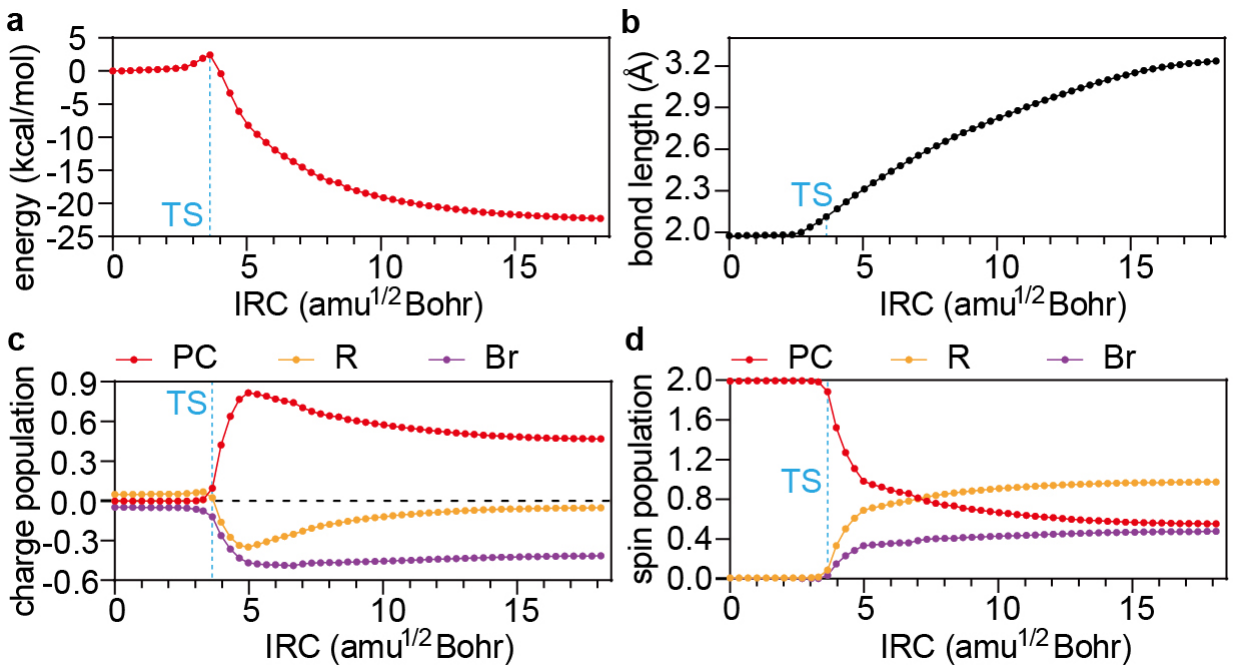
(**a**) Energy profile along the IRC of the PC1 activation reaction calculated by LOSC-UPBE0-D3BJ/def2-SVP/SMD-DMF. (**b**) Variation of the C−Br bond length. (**c**,**d**) Variation of the Mulliken charge (**c**) and spin (**d**) populations. TS: transition structure.

With the formation of the exciplex ^1,3^PC^*^/R−X, dissociative electron transfer ^1,3^PC^*^/R−X → PC^·+^/X^−^ + R^·^ can then occur. While the open-shell singlet reaction ^1^PC^*^/R−X → PC^·+^/X^−^ + R^·^ for PC2 has to be investigated by virtue of TDDFT, the open-shell triplet reactions ^3^PC^*^/R−X → PC^·+^/X^−^ + R^·^ for PC1, PC3, PC4, and PC5 can be analysed in terms of either UDFT or TDDFT. The former is adopted here because the intrinsic delocalisation errors inherent in approximate density functionals (which usually result in underestimated energy barriers) can, in this case, readily be cured by means of the localised orbital scaling correction (LOSC) scheme [36,37], i.e., LOSC-UPBE0-D3BJ/def2-SVP/SMD-DMF. Taking PC1 as an example (Figure 4a), the variation of the ^3^PC^*^/R−X geometry along the IRC is dominated by the elongation of the C−Br bond (Figure 4b). At the transition structure (TS), the C−Br bond length is 2.19 Å, while the energy barrier is 2.4 kcal/mol. Both values are larger than those without the LOSC (2.09 Å and 1.2 kcal/mol), reflecting the amount of charge delocalisation errors inherent in the PBE0 functional.

The change in the electronic structure of the PC1 ^3^PC^*^/R−X is monitored along the IRC by the net charge (Figure 4c) and spin (Figure 4d) on each of the moieties PC, R, and X. It can be seen that the electron transfer takes place in the vicinity of the TS from the PC to the R and somewhat more to the X (i.e., Br) moiety, clearly indicating that the electron transfer is responsible for the onset of the reaction and is thus rate-determining. After the peak electron transfer (shortly after the TS), the hole on the PC is gradually transferred to the R moiety, ending up with a R^·^ and a PC^·+^/X^−^. Therefore, the process can grossly be depicted as an electron transfer followed by a half-hole transfer from ^3^PC^*^ to R-X.

**Figure 5:**
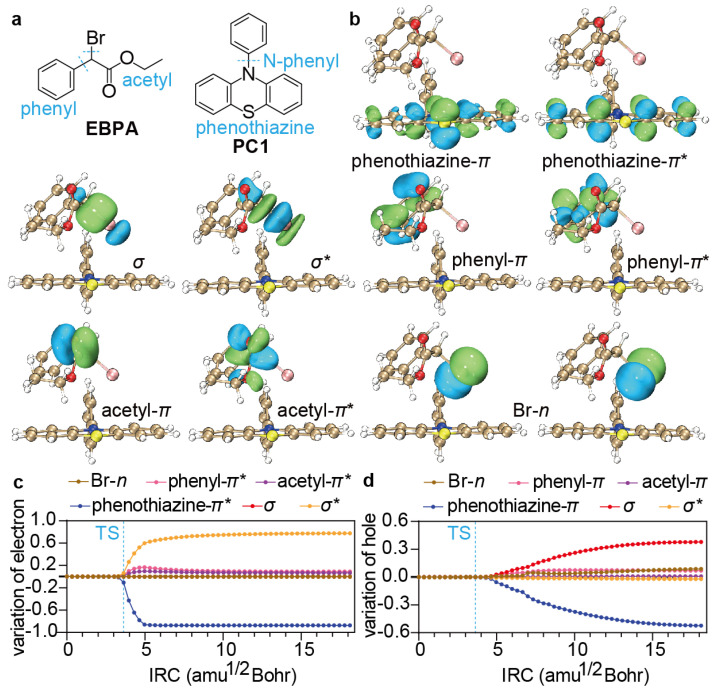
(**a**) Fragmentation of EBPA and PC1. (**b**) Representative ground-state FLMOs at the equilibrium of ^3^PC^*^/R−X. (**c**,**d**) Variation of the electron (**c**) and hole (**d**) populations on different FLMOs along the IRC.

To see the contributions of individual orbitals to the above electron/hole transfer, we take the ground state ($S_{0}$) of PC/R−X but at the equilibrium geometry of ^3^PC^*^/R−X as the reference, whose FLMOs [71] $\left\{\phi_{R_{0},p}^{\mathrm{FLMO}}\right\}$ can be constructed noniteratively in terms of the orthonormal primitive FLMOs from subsystem calculations [38,39] (phenyl, Br, and acetyl for EBPA and N-phenyl and phenothiazine for PC1; see Figure 5a) by using the BDF program package [54,56]. Representative reference FLMOs, including phenyl-$\pi /\pi^{*}$, acetyl-$\pi /\pi^{*}$, $\sigma /\sigma^{*}$, and Br-*n* for R−X, as well as phenothiazine-$\pi /\pi^{*}$ for PC, are shown in Figure 5b (see Figures S3 and S4 in the SI for additional FLMOs). The FLMOs $\left\{\phi_{R,p}^{\mathrm{FLMO}}\right\}$ at any geometry can also be constructed in the same way. The occupied spin orbitals $\left\{\phi_{R,i\sigma }^{\mathrm{FLMO}}\right\}$ of ^3^PC^*^/R−X are then expanded in terms of the reference FLMOs $\phi_{R_{0},p}^{\mathrm{FLMO}}$ with the coefficients $C_{pi}^{\sigma }\left(R\right)$ such that the total number ($N^{\sigma }$) of electrons of spin σ can be written as $N^{\sigma }=\sum_{p}n_{p}^{\sigma }\left(R\right)$, with $n_{p}^{\sigma }\left(R\right)=\sum_{i}n_{i}^{\sigma }{\left|C_{pi}^{\sigma }\left(R\right)\right|}^{2}$. The variation $\Delta n_{p}^{\sigma }\left(R\right)$ of $n_{p}^{\sigma }\left(R\right)$ with respect to $n_{p}^{\sigma }\left(R_{0}\right)$ reflects the change in the σ-spin occupation number (ranging between 0∼1) of $\phi_{R,p}^{\mathrm{FLMO}}$ as compared to that at the equilibrium geometry of ^3^PC^*^/R−X. Since the excited electron of ^3^PC^*^ is associated with α-spin, $\Delta n_{p}^{\alpha }\left(R\right)$ will reveal the amount of transfer of the excited electron. Similarly, $1-\Delta n_{p}^{\beta }\left(R\right)$ is a good indicator of the hole transfer since the hole has β-spin. It is then clear that the electron transfer takes place predominantly from phenothiazine-$\pi^{*}$ to $\sigma^{*}$ in the vicinity of the TS (see Figure 5c), after the peak of which a half-hole transfer occurs from phenothiazine-π mostly to σ (see Figure 5d).

As such, the energy barrier $\Delta E^{‡}$ for the dissociative electron transfer ^1,3^PC^*^/R−X → PC^·+^/X^−^ + R^·^ mainly represents the feasibility of the $\pi^{*}\to \sigma^{*}$ electron transfer process: the lower the $\Delta E^{‡}$, the faster the process. The calculated $\Delta E^{‡}$ are documented in Table 1.

### 4.4. Deactivation and Side Reactions

**Figure 6:**
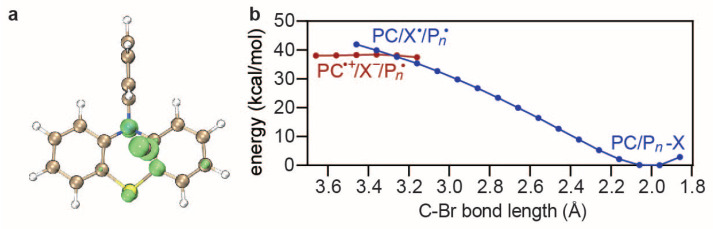
Net Mulliken spin populations of PC^·+^/X^−^ (**a**) as well as a relaxed scan of the C−Br bond between the $sp^{2}$ alkyl-C of P_*n*_^·^ and the Br of PC^·+^/X^−^ (**b**) using UPBE0-D3BJ/def2-SVP/SMD-DMF.

The activation step produces a complex PC^·+^/X^−^ as well as a R^·^ growing into a polymer P_*n*_^·^, after which the deactivation step PC^·+^/X^−^ + P*n*· → PC + $P_{n}$−X can take place to complete the catalytic cycle. Since PC^·+^/X^−^/P_*n*_^·^ bears a singlet bi-radical character, a symmetry-broken initial guess should be taken in the UPBE0-D3BJ/def2-SVP/SMD-DMF calculations. Taking PC1 as an example, Figure 6a shows that the unpaired electron of PC^·+^/X^−^ is mostly polarised on the Br atom, which is favorable [50,51,52] for a bi-radical reaction with the $sp^{2}$ alkyl-C of P_*n*_^·^. To further confirm this, a relaxed scan that shortens the distance between the Br of PC^·+^/X^−^ and the $sp^{2}$ alkyl-C of P_*n*_^·^ from a value of 3.6 Å to the equilibrium (2.0 Å) is performed, which describes two diabatic states: (i) PC^·+^/X^−^ and P_*n*_^·^ with a negative charge localised on X^−^, positive charge localised on PC^·+^, and an unpaired electron localised on P_*n*_^·^; (ii) PC/P*_n_*−X with a virtually neutral PC and R-X. The result reveals an almost monotonically decreasing energy profile, ending with PC/P*_n_*−X as the product (see Figure 6b). Although the deactivation energy barrier $\Delta E_{\mathrm{de}}^{‡}$ is nearly zero for PC1, it can be a very important descriptor in other cases. Therefore, $\Delta E_{\mathrm{de}}^{‡}$ is used here as the fourth descriptor.

This deactivation step may be impeded by the possible dissociation of PC^·+^/X^−^ into PC^·+^ and Br^−^, or alternatively into ground-state PC and Br^·^. We ruled out the potential pathway to produce PC and Br^·^ by calculating the dissociation energy of PC^·+^/X^−^. Taking PC1 as an example, the dissociation energy is 21.7 kcal/mol, which is significantly higher than the value of 15.7 kcal/mol required to produce PC^·+^ and Br^−^. Thus, the formation of PC^·+^ and Br^−^ appears to be the more likely pathway. PC^·+^ can react with species such as radical R^·^ [34] and solvent molecules [51], thus breaking the catalytic cycle. Even worse, the unpaired electron in PC^·+^ resides mainly on S and N, so that a side reaction may occur between S and P_*n*_^·^ (NB: N is blocked by phenyl-H and is hence not reactive; see Figure S6a in the SI). To confirm this, the distance between the S of PC^·+^ and the $sp^{2}$ alkyl-C of P_*n*_^·^ is shortened from a value of 3.5 Å to the equilibrium (2.0 Å) in a relaxed scan, which does reveal a monotonically decreasing energy profile (see Figure S6b in SI), leading to an unwanted product PC−P_*n*_^+^. Clearly, the only way to prevent this is a large enough binding strength $\Delta E_{b}^{\mathrm{PC}-X}$ so as to resist the dissociation of PC^·+^/X^−^. As can be seen from Table 1, the $\Delta E_{b}^{\mathrm{PC}-X}$ amounts to 15.7 kcal/mol for PC1, resulting from the stabilisation of the negatively charged Br atom by the positively charged phenyl-H. A similar situation occurs for PC4, with $\Delta E_{b}^{\mathrm{PC}-X}$ being 16.0 kcal/mol. The $\Delta E_{b}^{\mathrm{PC}-X}$ for PC2 is much lower (13.4 kcal/mol) since the Br can only be weakly attracted by the peripheral H of the perylene plane. In contrast, PC5 and PC3 have two and multiple phenyl-H to stabilise the Br, respectively, such that they have much larger $\Delta E_{b}^{\mathrm{PC}-X}$ (17.4 and 22.2 kcal/mol, respectively). In addition, high $\Delta E_{b}^{\mathrm{PC}-X}$ for PC^·+^/X^−^ to produce PC^·+^ and X^−^, together with the dilute reaction condition for PC, especially when low catalyst loading is desired for polymerisation control, make it less possible for termolecular encounters to occur among PC^·+^, X^−^ and R^·^ in the deactivation step.

The polymer P_*n*_−X resulting from the deactivation can then bind with another ^1,3^PC^*^ to form an exciplex ^1,3^PC^*^/P_*n*_−X, with the binding strength $\Delta E_{b}^{\mathrm{ex}}$ being 11.6 kcal/mol, which is very much the same as that (11.2 kcal/mol) between ^1,3^PC^*^ and R−X (see Section S5 in the SI for more details). This is not surprising as the P*_n_* moiety of P_*n*_−X and the R moiety of R−X have similar chemical groups and differ only in that the former is more bulky in size (Section S5 in the SI). For simplicity, the $\Delta E_{b}^{\mathrm{ex}}$ for ^1,3^PC^*^/R−X is also used to represent that for ^1,3^PC^*^/P_*n*_−X below.

### 4.5. Validity of New Descriptors

To correlate more directly with the performances of PCs in O-ATRP, the descriptors in the energy representation $\Delta E^{X}$ ($=\Delta E_{b}^{\mathrm{ex}},\Delta E^{‡},\Delta E_{b}^{\mathrm{PC-X}},\Delta E_{\mathrm{de}}^{‡}$) are further converted to a lifetime representation $\tau_{1/2}^{X}$ ($=\tau_{b}^{\mathrm{ex}},\tau^{‡},\tau_{b}^{\mathrm{PC-X}},\tau_{\mathrm{de}}^{‡}$) according to [72]

(9) $$\tau_{1/2}^{X}=\frac{ln2}{k^{X}}$$

(10) $$\;k^{X}=\frac{k_{B}T}{h}e^{\frac{-\Delta E^{X}}{k_{B}T}}$$

**Figure 7:**
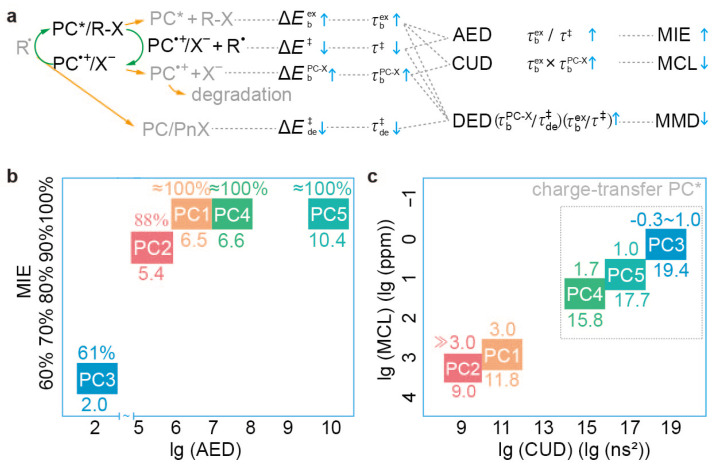
Derivation of the activation efficiency descriptor (AED) $\tau_{b}^{\mathrm{ex}}/\tau^{‡}$, catalyst utilisation descriptor (CUD) $\tau_{b}^{\mathrm{ex}}\times \tau_{b}^{\mathrm{PC-X}}$ and deactivation efficiency descriptor (DED), $\left(\tau_{b}^{\mathrm{PC-X}}/\tau_{\mathrm{de}}^{‡}\right)/\left(\tau_{b}^{\mathrm{ex}}/\tau^{‡}\right)$ (**a**) to correlate with the maximum initiator efficiency (MIE) (**b**) and minimum catalyst loading (MCL) (**c**), respectively. lg(x) stands for the logarithmic value of the AED, MCL, and CUD. See Section S6 in SI for more details.

The results are documented in Table 1. Both $\tau_{b}^{\mathrm{ex}}$ and $\tau^{‡}$ are related to the activation step. The former tells how long the exciplex ^1,3^PC^*^/R−X can exist prior to its dissociation, whereas the latter indicates how long it takes ^1,3^PC^*^/R−X to undergo a dissociative electron transfer. As such, their ratio $\tau_{b}^{\mathrm{ex}}/\tau^{‡}$ reflects how likely the dissociative electron transfer is to occur before ^1,3^PC^*^/R−X dissociates, thereby being a good ‘activation efficiency descriptor’ (AED); the larger the ratio $\tau_{b}^{\mathrm{ex}}/\tau^{‡}$, the more efficient the activation (cf. Figure 7a). A higher activation efficiency then implies a faster conversion of R−X initiators to polymer chains, thereby rendering a higher ‘maximum initiator efficiency’ (MIE; the highest possible $I^{*}$ that can be achieved with sufficient catalyst loading). This is indeed the case for PC1-5, as can be seen from Figure 7b [cf. Table 1. The MIE for PC2 is revised based on reported data [48] (see Section S6 and Table S5 in SI for more details); the MIE for PC6-11 is summarised in Table S7 in the SI].

In contrast, $\tau_{b}^{\mathrm{PC-X}}$ is related to the deactivation step, for it tells how long the ion pair PC^·+^/X^−^ can survive to complete the catalytic cycle (i.e., PC^·+^/X^−^ + P*n*· → PC/P*n*−X). To restart the catalytic cycle, a new exciplex ^1,3^PC^*^/P_*n*_−X must be prevented from dissociating to ^1,3^PC^*^ and P_*n*_−X. As such, the longer lifetimes of both PC^·+^/X^−^ ($\tau_{b}^{\mathrm{PC-X}}$) and ^1,3^PC^*^/P_*n*_−X ($\tau_{b}^{\mathrm{ex}}$) can keep more catalysts “at work” to complete and restart the catalytic cycle repeatedly for the alternating growth of polymer chains so that their product $\tau_{b}^{\mathrm{ex}}\times \tau_{b}^{\mathrm{PC-X}}$ can be a ‘catalyst utilisation descriptor’ (CUD; cf. Figure 7a). Since the CUD is reflected by the ‘minimum catalyst loading’ (MCL) to achieve satisfactory polymerisation control (ideally $I^{*}\approx 100\%$ and as low a *Đ* as possible), it is not surprising that the CUD is well correlated with the MCL for PC1-5, as can be seen from Figure 7c [cf. Table 1; the MCL for PC2 is revised based on reported data [48] (see Section S6 and Table S5 in SI for more details); the MCL for PC6-11 is summarised in Table S7 in the SI].

Lastly, $\tau_{\mathrm{de}}^{‡}$ is also associated with the deactivation step, serving as a crucial indicator of the kinetic efficiency of the deactivation reaction (PC^·+^/X^−^ + P*n*· → PC/P*n*−X). However, it is widely recognised that achieving effective control in O-ATRP relies on more efficient deactivation than activation. This ensures that all activated chains can quickly transition to a dormant state. Clearly, the ratio $\left(\tau_{b}^{\mathrm{PC-X}}/\tau_{\mathrm{de}}^{‡}\right)$ represents the deactivation efficiency, whereas $\left(\tau_{b}^{\mathrm{ex}}/\tau^{‡}\right)$ represents the activation efficiency. The overall ratio $\left(\tau_{b}^{\mathrm{PC-X}}/\tau_{\mathrm{de}}^{‡}\right)/\left(\tau_{b}^{\mathrm{ex}}/\tau^{‡}\right)$ then indicates the relative efficiency of deactivation compared to activation, which is then a good ‘deactivation efficiency descriptor’ (DED). A higher value of DED indicates that deactivation is significantly more efficient than activation. Evidently, the DED only needs to attain a sufficient value to ensure that the deactivation reaction constantly deactivates the growing chains, causing them to be dormant, preventing their accumulation (leading to dead chains and much increased *Đ*), and preserving polymerisation control. Therefore, when the DED is adequately high, the ‘minimum molecular weight dispersity’ (MMD) will be low. Conversely, if the DED is too low, the MMD will be significantly high. This is indeed the case for PC1-5 with different structures and PC5-11 with similar structures. Particularly for PC7, PC8, and PC11, the MMD is higher than other PCs, indicating the less efficient alternating growth of polymer chains. The DED values for PC1-11 in O-ATRP can be deduced from Table 1 and seen from Table S7 in the SI.

Overall, PC5 performs the best within PC1-5. Still, however, it is not as good as PC3 as far as the MCL is concerned. This stems from the fact that its $\tau_{b}^{\mathrm{PC-X}}$ ($6.3\times 10^{8}$ ns) is much shorter than that ($2.1\times 10^{12}$ ns) of PC3. As such, there is still much room for optimising the PCs to achieve better polymerisation control at a lower catalyst loading in O-ATRP. The key for performing this optimisation is outlined as follows:

1. Interaction between ^3^PC^*^ and R-X: PC5 features a sizable conjugated chromophore core with six conjugated ligands attached in a nearly orthogonal configuration. The attached aryl ligands, being electron-donating compared to the electron-withdrawing dihydrophenazine chromophore core, result in low-lying charge-transfer states, particularly ^3^PC^*^, facilitating its interaction with R-X and thereby enhancing $\Delta E_{b}^{\mathrm{ex}}$. Additionally, the orthogonal arrangement exposes the aryl ligands’ peripheral hydrogen binding sites to both sides of the chromophore plane, allowing R-X to leverage these sites, further boosting $\Delta E_{b}^{\mathrm{ex}}$.
2. Electron affinity balance: The electron affinities and the disparity between the chromophore core and ligands are important for overall analysis. PC5 achieves a delicate balance, ensuring that while constructing low-lying charge-transfer states is feasible, the overall electron affinity is neither excessively strong nor weak. This balance allows ^3^PC^*^ to donate an electron to R-X, facilitating a low $\Delta E^{‡}$ for dissociative electron transfer. Simultaneously, PC^·+^ maintains sufficient oxidation, ensuring a low enough $E_{\mathrm{de}}^{‡}$ for the deactivation reaction. If the chromophore core and ligands are too electron-withdrawing or electron-donating, inefficient activation or deactivation occurs, leading to poor polymerisation control.
3. Br atom placement in PC^·+^/X^−^: Similar to PC3, PC^·+^/X^−^ in PC5 positions the Br atom within an “H-nest” formed by peripheral H atoms of orthogonal ligands attached to the chromophore core. This configuration results in a high $\Delta E_{b}^{\mathrm{PC-X}}$, maximising the stabilisation of PC^·+^/X^−^ and minimising the loss of reactive PC species within the catalytic cycle. Consequently, this design reduces catalyst loading, contributing to effective polymerisation control.

So far, no single PC candidate has managed to excel in all three aspects simultaneously. PC5 meets the criteria for aspects 1 and 2, while PC3 excels in aspect 3. Therefore, the foundation for future optimisation can either begin with PC5, focusing on aspect 3, or start with PC3, targeting aspects 1 and 2.

## 5. Conclusions

Four descriptors have been identified to elucidate the activation and deactivation steps of the PCs in O-ATRP, viz., the binding strength $\Delta E_{b}^{\mathrm{ex}}$ of ^1,3^PC^*^/R−X, the energy barrier $\Delta E^{‡}$ for the dissociative electron transfer, the binding strength $\Delta E_{b}^{\mathrm{PC-X}}$ of PC^·+^/X^−^, and the deactivation energy barrier $\Delta E_{\mathrm{de}}^{‡}$ for the deactivation step. The descriptors can further be converted to an equivalent lifetime representation ($\tau_{b}^{\mathrm{ex}}$, $\tau^{‡}$, $\tau_{b}^{\mathrm{PC-X}}$, and $\tau_{\mathrm{de}}^{‡}$, respectively), such that the compound descriptors $\tau_{b}^{\mathrm{ex}}/\tau^{‡}$ (AED), $\tau_{b}^{\mathrm{ex}}\times \tau_{b}^{\mathrm{PC-X}}$ (CUD), and $\left(\tau_{b}^{\mathrm{PC-X}}/\tau_{\mathrm{de}}^{‡}\right)/\left(\tau_{b}^{\mathrm{ex}}/\tau^{‡}\right)$ (DED) can be derived to characterise the MIE, MCL, and MMD, respectively. In particular, the much lower MCL in the case of a charge-transfer ^1,3^PC^*^, as compared to the locally excited counterpart, has been successfully explained by the charge separation that greatly enhances the binding strength $\Delta E_{b}^{\mathrm{ex}}$ between the charge-transfer ^1,3^PC^*^ and the polar initiator R−X, which then raises the CUD and lowers the MCL. Based on analyses carried out here, guidelines for optimising O-ATRP catalysts can be updated to: (i) modify the chemical/geometric structure of the PC to achieve better charge separation in ^1,3^PC^*^ and thus a higher binding strength $\Delta E_{b}^{\mathrm{ex}}$ between ^1,3^PC^*^ and R−X; (ii) use a more electron-donating chromophore core and substituents in the PC to lower the barrier $\Delta E^{‡}$ for the dissociative electron transfer; and (iii) modify the chemical/geometric structure of the PC to achieve a higher binding strength $\Delta E_{b}^{\mathrm{PC-X}}$ between PC^·+^ and X^−^.

In comparison to prevailing approaches primarily focused on the redox potentials associated with the PC, the property descriptor approach presented in this study considers both the PC and R-X, emphasising their interactions. A key strength lies in its consideration of binding strengths within ^3^PC^*^/R−X and PC^·+^/X^−^, often overlooked but demonstrated as significant factors in this work. This approach is thus inherently more detailed and reliable than the previous approach since the activation and deactivation steps are highly associated with interactions between the PC and R-X.

In this way, the next step is to employ the new property descriptor approach by taking account of the previously neglected interactions between the PC and R-X. This not only accelerates the development of PCs for O-ATRP but also further validates the advantages of the property descriptor approach. As the pursuit of exceptional O-ATRP performance has reached an advanced stage, further optimisation of organic PCs must encompass considerations from different aspects of the catalytic cycle. The property descriptor approach, grounded in property–performance relationships, thus becomes important for the future rational design of organic PCs for O-ATRP, which need to balance synthetic feasibility, sustainability, cost, and adaptability to polymerisation systems as well.

## Figures and Tables

**Table 1:** Performance of catalysts in O-ATRP and calculated molecular descriptors.

		Polymerisation *^a^*			Descriptor *^b^*							
No.	Catalyst	Loading ppm	$I^{*}$ *^c^*	*Đ*	$\Delta E_{b}^{\mathrm{ex}}$ kcal/mol	$\tau_{b}^{\mathrm{ex}}$ ns	$\Delta E^{‡}$ kcal/mol	$\tau^{‡}$ ns	$\Delta E_{b}^{\mathrm{PC-X}}$ kcal/mol	$\tau_{b}^{\mathrm{PC-X}}$ ns	$\Delta E_{\mathrm{de}}^{‡}$ kcal/mol	$\tau_{\mathrm{de}}^{‡}$ ns
1	PC1 [26,52]	1000	116%	1.30	11.2	1.8 × $10^{4}$	2.4	6.4 × $10^{-3}$	15.7	3.6 × $10^{7}$	0.4	2.2 × $10^{-4}$
2	PC2 [48]	1000	45%	1.39	9.7	1.5 × $10^{3}$	2.4	6.4 × $10^{-3}$	13.4	7.4 × $10^{5}$	0.0	1.1 × $10^{-4}$
3a	PC3 [27]	10	61%	1.30	15.0	1.1 × $10^{7}$	12.3	1.2 × $10^{5}$	22.2	2.1 × $10^{12}$	1.7	2.0 × $10^{-3}$
3b		0.5	53%	1.39								
4	PC4 [53]	50	91%	1.29	16.3	9.4 × $10^{7}$	7.3	2.5 × $10^{1}$	16.1	7.2 × $10^{7}$	1.9	2.8 × $10^{-3}$
5a	PC5 [28]	10	92%	1.27	17.5	7.6 × $10^{8}$	3.3	2.9 × $10^{-2}$	17.4	6.3 × $10^{8}$	3.9	8.1 × $10^{-2}$
5b		50	106%	1.14								

Note: ${\;}^{a}$  Results with the EBPA (Nos. 1 and 2) or a similar diethyl-2-bromo-2-methylmalonate (Nos. 3, 4, and 5) initiator solvated in DMF (No. 3) or DMAc (Nos. 1, 4, and 5) except for No. 2 (not solvated), methyl methacrylates as the monomer expect for No. 2 (benzyl acrylates), and a formulation of [monomer]:[initiator] = 100:1 except for Nos. 3 and 4 (200:1). The overall best polymerisation results with monomer conversions proceeding to ≥50% are selected here for each PC to maximise comparability. ${\;}^{b}$ $\Delta E^{‡}$ calculated with (LOSC-) PBE0-D3BJ/def2-SVP/SMD-DMF for PC1, PC3, PC4, and PC5, and TDDFT/PBE0-D3BJ/def2-SVP/SMD-DMF for PC2; $\Delta E_{b}^{\mathrm{ex}}$, $\Delta E_{b}^{\mathrm{PC-X}}$ and $\Delta E_{\mathrm{de}}^{‡}$ calculated with UPBE0-D3BJ/def2-SVP/SMD-DMF for all PCs. ${\;}^{c}$ The value of $I^{*}$≫100% indicates autoinitiation or the occurrence of other side reactions.

**Table 2:** Reported photoexcitaion properties for PC1–5.

Catalyst Unit	$\lambda_{\max}$ nm	$\epsilon_{\max}$ L/mol·cm	$\Phi_{S}$ ${\;}^{a}$	$\tau_{S}$ ${\;}^{b}$ ns	$\Phi_{T}$ ${\;}^{c}$	$\tau_{T}$ ${\;}^{d}$ ns
PC1 ^*e*^ [65,66]	319	4900	28%	3.4	72%	$7.2\times 10^{7}$
PC2 ${\;}^{f}$ [67]	440	34,000	∼100%	4.0	∼0%	–
PC3 ${\;}^{g}$ [27,68]	468	13,900	23%	2.4	77%	$3.8\times 10^{8}$
PC4 ${\;}^{h}$ [53]	398	3900	35%	18	65%	$3.0\times 10^{3}$
PC5 ${\;}^{i}$ [28]	388	20,000	–	–	–	–

Note: ${\;}^{a}$ $\Phi_{S}=1-\Phi_{T}$. ${\;}^{b}$  Estimated using the fluorescence lifetime [53,66,67,68]. ${\;}^{c}$ $\Phi_{T}$ of PC1 is estimated using the phosphoresence quantum yield; $\Phi_{T}$ of PC2 is taken as the intersystem crossing quantum yield calculated from the fluoresence quantum yield along with the fluorescence and internal conversion rate constants [67]; $\Phi_{T}$ of PC3 [68] and PC4 [53] are taken as the intersystem crossing quantum yields. ${\;}^{d}$ Estimated using the phosphorescence lifetime [53,66,67,68]. ^*e*^ Photoexcitation data for PC1 are all measured in tetrahydrofuran [66], except that $\epsilon_{\max}$ is measured in cyclohexane [65]. ${\;}^{f}$ Photoexcitation data for PC2 are all measured in toluene [67]. ${\;}^{g}$ Photoexcitaion data for PC3 are all measured in acetonitrile [68], except that $\lambda_{\max}$ and $\epsilon_{\max}$ are measured in DMF. ${\;}^{h}$ Photoexcitation data for PC4 are all measured in toluene [67]. ${\;}^{i}$ Photoexcitation data for PC5 are all measured in N,N-dimethylacetamide [28].

**Table 3:** Experimental and calculated excited-state oxidation potentials (in eV; relative to the saturated calomel electrode) for PC1-5.

Catalyst	Solvent	$E^{0}$(PC^·+^/^1^PC^*^)/$E^{0}$(PC^·+^/^3^PC^*^)		
		Experiment	This Work ^*a*^	This Work ^*ab*^
PC1	cyclohexane	−1.92/−1.70 [52]	−2.17/−1.57	−2.91/−2.31
PC2	toluene	−1.87/−0.70 [48]	−1.70/−0.41	−2.12/−1.05
PC3	acetonitrile	−1.58/−1.41 [27]	−1.77/−1.58	−1.77/−1.59
PC4	chloroform	−1.79/−1.76 ${\;}^{c}$ [53]	−1.94/−1.94	−2.22/−2.21
PC5	toluene	−1.84 [28]/–	−2.03/−1.58	−2.52/−2.10

Note: ${\;}^{a}$ Calculated with TDDFT/PBE0-D3BJ/def2-SVP and SMD. ${\;}^{b}$ Calculated with DMF as the solvent. ${\;}^{c}$ Derived from ground-state oxidation potentials and photoluminescence data in the literature [53]. Further insights into the variations in the properties of different PCs can be found in our recent review [17].

## Data Availability

Data are available upon request to the corresponding author.

## Supplementary Materials

The following supporting information can be downloaded at https://www.mdpi.com/article/10.3390/polym16030323/s1: Figure S1: Illustration of the electron and hole of ^3^PC^*^ (represented by the α-HOMO and β-LUMO, respectively) for PC1, PC3, PC4, and PC5, as well as ^1^PC^*^ (represented by the LUMO and HOMO, respectively, in the case of HOMO→LUMO-dominated $S_{1}$ excitation) for PC2. ^3^PC^*^ and ^1^PC^*^ are calculated by UPBE0/def2-SVP/SMD-DMF and TDDFT/PBE0/def2-SVP/SMD-DMF, respectively; Figure S2: ^3^PC^*^ (PC1, PC3, PC4, and PC5) and ^1^PC^*^ (PC2) electrostatic potential maps [63,64] on the van der Waals surfaces at their respective equilibrium geometries, calculated by UPBE0/def2-SVP/SMD-DMF and TDDFT/PBE0/def2-SVP/SMD-DMF, respectively; Figure S3: Ground-state ($S_{0}$) regional FLMOs corresponding to the six phenyl-$\pi /\pi^{*}$ of PC/R−X but at the equilibrium geometry of ^3^PC^*^/R−X calculated by PBE0/def2-SVP; Figure S4: Ground-state ($S_{0}$) regional FLMOs corresponding to the fourteen phenothiazine-$\pi /\pi^{*}$ of PC/R−X but at the equilibrium geometry of ^3^PC^*^/R−X calculated by PBE0/def2-SVP; Figure S5: (a) Chemical structures of R−X (EBPA), PC1, and P_*n*_−X. (b,c) Equilibrium geometries of the exciplexes ^3^PC^*^/R−X (b) and ^3^PC^*^/P_*n*_−X (c) for PC1 calculated by UPBE0-D3BJ/def2-SVP/SMD-DMF; Figure S6: Net Mulliken spin populations of PC^·+^ (a) as well as a relaxed scan of the C−S bond between the $sp^{2}$ alkyl-C of P_*n*_^·^ and the S of PC^·+^ (b) using UPBE0-D3BJ/def2-SVP/SMD-DMF; Figure S7: Chemical structures of core-substituted diaryl dihydrophenazines (PC5-11); Table S1: Experimental and calculated excited-state oxidation potentials (in eV; relative to the saturated calomel electrode) for PC1-5; Table S2: Calculated excited-state oxidation potentials (in eV; relative to the saturated calomel electrode; Table S3: Calculated triplet excited-state oxidation potentials (in eV; relative to the saturated calomel electrode) by UDFT for PC1-5; Table S4: Adiabatic and vertical energy differences between $S_{1}$ and $T_{1}$, dominant configurations of both excitations, and $2(\psi_{L}\psi_{H}|\psi_{L}\psi_{H})$ for PC1-5; Table S5: Polymerisation performances of PC1-5 in O-ATRP; Table S6: Polymerisation performances of PCs 5-11 in O-ATRP; Table S7: Performances of catalysts in O-ATRP and calculated molecular descriptors.

## Abbreviations

The following abbreviations are used in this manuscript:

PC	Photocatalyst
O-ATRP	Organocatalysed atom transfer radical polymerisation
OQP	Oxidative quenching pathway
R-X	Initiator
$I^{*}$	Initiator efficiency
*Đ*	Molecular weight dispersity
PC^*^	Excited-state PC
^1,3^PC^*^	First singlet/triplet excited-state PC
S_1_/T_1_	First singlet/triplet state
$\Phi_{S/T}$	Singlet/triplet quantum yield
$\tau_{S/T}$	Lifetime of first singlet/triplet excited state of PC
$k_{\mathrm{act}}$	Overall rate constant for activation
$k_{f}$	Rate constant for the diffusion-controlled encounter of PC^*^ and R-X to form an exciplex
$k_{d}$	Rate constant for the dissociation of the exciplex to PC^*^ and R-X
$K_{c}$	Formation/dissociation equilibrium constant for the exciplex
$k_{\mathrm{ET}}$	Rate constant for dissociative electron transfer in the exciplex
*T*	Temperature
η	Viscosity of the solvent
$k_{B}$	Boltzmann constant
*h*	Planck constant
$\Delta G^{‡}$	Gibbs free energy barrier for activation
$\Delta E^{‡}$	Electronic energy part of $\Delta G^{‡}$
$\Delta G_{0}$	Gibbs free energy change of electron transfer for activation
λ	Sum of the solvent reorganisation energy and the energy required to break the R-X bond
$\Delta E_{b}^{\mathrm{ex}}$	Binding strength between PC^*^ and R-X
$\Delta E_{\mathrm{de}}^{‡}$	Electronic energy barrier for deactivation
$\Delta E_{b}^{\mathrm{PC-X}}$	Binding strength between PC^·+^ and X^−^
EBPA	Ethyl-α-bromophenylacetate
TDDFT	Time-dependent density functional theory
LOSC	Localised orbital scaling correction
SMD	Density-based implicit solvation model
HOMO	Highest occupied molecular orbital
LUMO	Lowest unoccupied molecular orbital
$\psi_{H/L}$	Orbital wavefunction of HOMO/LUMO
IRC	Intrinsic reaction coordinate
FLMO	Fragment-localised molecular orbital
$\tau^{‡}$	Lifetime representation for $\Delta E^{‡}$
$\tau_{b}^{\mathrm{ex}}$	Lifetime representation for $\Delta E_{b}^{\mathrm{ex}}$
$\tau_{\mathrm{de}}^{‡}$	Lifetime representation for $\Delta E_{\mathrm{de}}^{‡}$
$\tau_{b}^{\mathrm{PC-X}}$	Lifetime representation for $\Delta E_{b}^{\mathrm{PC-X}}$
MIE	Maximum initiator efficiency
AED	Activation efficiency descriptor
MCL	Minimum catalyst loading
CUD	Catalyst utilisation descriptor
MMD	Minimum molecular weight dispersity
DED	Deactivation efficiency descriptor
