# Supplementary material for: Toward the Rational Design of Organic Catalysts for Organocatalysed Atom Transfer Radical Polymerisation
Source: Polymers (Basel). 2024 Jan 24;16(3):323. doi: 10.3390/polym16030323 (PMC10857621; doi:10.3390/polym16030323)
Supplement: Supplementary file 1 [file polymers-16-00323-s001.zip › polymers-2818220-supplementary.pdf]

Supporting Information
Toward Rational Design of Organic Catalysts for Organocatalysed Atom Transfer Polymerisation

Zhilei Wang, Chenyu Wu,* and Wenjian Liu*

Institute of Frontier Chemistry, School of Chemistry and Chemical Engineering, Shandong University, Qingdao 266237, China; 201920282@mail.sdu.edu.cn

E-mail: w@sdu.edu.cn (C.W.); liuwj@sdu.edu.cn (W.L.)

S1. Excited-State Redox Potentials

The oxidation potentials $E^0(\mathrm{PC}^{\cdot+}/{}^{1,3}\mathrm{PC}^*)$ of the lowest singlet/triplet excited state ${}^{1,3}\mathrm{PC}^*$ of a photocatalyst (PC) can be calculated as

$$E^0(\mathrm{PC}^{\cdot+}/{}^{1,3}\mathrm{PC}^*) = G_{\mathrm{SCE}} - \Delta G(\mathrm{PC}^{\cdot+}/{}^{1,3}\mathrm{PC}^*) \approx G_{\mathrm{SCE}} - \Delta E(\mathrm{PC}^{\cdot+}/{}^{1,3}\mathrm{PC}^*), \tag{S1}$$

$$\Delta E(\mathrm{PC}^{\cdot+}/{}^{1,3}\mathrm{PC}^*) = E({}^{1,3}\mathrm{PC}^*) - E(\mathrm{PC}^{\cdot+}), \tag{S2}$$

where $G_{\mathrm{SCE}} = -4.60$ eV[1,2] is the reduction Gibbs free energy of the saturated calomel electrode, whereas $\mathrm{E}({}^{1,3}\mathrm{PC}^*)$ and $\mathrm{E}(\mathrm{PC}^{\cdot+})$ are the electronic energies of ${}^{1,3}\mathrm{PC}^*$ and $\mathrm{PC}^{\cdot+}$ at their equilibrium geometries, respectively. Time-dependent density functional theory (TD-DFT) is used herein in conjunction with the def2-SVP basis set[3] and the density-based implicit solvation model (SMD).[4] To check the accuracy of density functionals, B3LYP[5,6] with the D3BJ[7] dispersion correction (B3LYP-D3BJ), PBE0[8]-D3BJ, M06-2X[9] with the D3[10]

dispersion correction (M06-2X-D3), CAM-B3LYP[11]-D3BJ, and ωB97X-D3[12] are compared. The results, calculated with the same solvents as used in the experimental measurements, are shown in Table S1. As compared to the experimental $E^0(\mathrm{PC}^{\cdot+}/^1\mathrm{PC}^*)$ [$E^0(\mathrm{PC}^{\cdot+}/^3\mathrm{PC}^*)$], B3LYP-D3BJ and PBE0-D3BJ result in the smallest errors on average, being 0.18 (0.15) and 0.19 (0.19) eV, respectively. PBE0-D3BJ/def2-SVP/SMD is then used throughout. For a direct comparison of PCs, it is more meaningful to calculate $E^0(\mathrm{PC}^{\cdot+}/^{1,3}\mathrm{PC}^*)$ with the solvents commonly used for O-ATRP, i.e., N,N-dimethylformamide (DMF) and N,N-dimethylacetamide (DMAc). As shown in Table S2, the results with DMF are virtually the same as those with DMAc, and thus DMF is used as a representative throughout this work.

Table S1: Experimental and calculated excited-state oxidation potentials (in eV; relative to the saturated calomel electrode) for PC1-5.

catalyst	solvent	$E^0(\mathrm{PC}^{\cdot+}/^1\mathrm{PC}^*)/E^0(\mathrm{PC}^{\cdot+}/^3\mathrm{PC}^*)^a$					
		experiment	B3LYP^b	PBE0^b	M06-2X^c	CAM-B3LYP^b	ωB97X-D3^d
PC1	cyclohexane	-1.92/-1.70[13]	-2.19/-1.65	-2.17/-1.57	-2.22/-1.66	-2.44/-1.72	-2.43/-1.70
PC2	toluene	-1.87/-0.70[14]	-1.75/-0.52	-1.70/-0.41	-1.69/-0.50	-1.89/-0.53	-1.87/-0.51
PC3	acetonitrile	-1.58/-1.41[15]	-1.78/-1.65	-1.77/-1.58	-2.09/-1.65	-2.30/-1.77	-2.39/-1.73
PC4	chloroform	-1.79/-1.76^e[16]	-1.89/-1.89	-1.94/-1.94	-2.53/-2.23	-2.86/-2.39	-3.11/-2.41
PC5	toluene	-1.84[17]/ –	-2.04/-1.65	-2.03/-1.60	-2.21/-1.73	-2.51/-1.87	-2.59/-1.91
average error^f		0.00/0.00	0.18/0.15	0.19/0.19	0.42/0.24	0.60/0.30	0.68/0.29

Note: ^{a}Calculated with TDDFT/PBE0-D3BJ/def2-SVP and the SMD solvation model. ^{b}Dispersion correction D3BJ[7] is employed. ^{c}Dispersion correction D3[10] is employed. $^d\omega$B97X-D3 is reoptimized from ωB97X-D with the D3 dispersion correction.[12] ^{e}Derived from the ground-state oxidation potentials and photoluminescence data in literature.[16] ^{f}Absolute difference between the calculated and experimental $E^0(\mathrm{PC}^{\cdot+}/^1\mathrm{PC}^*)$ [$E^0(\mathrm{PC}^{\cdot+}/^3\mathrm{PC}^*)$] being averaged over PC1-5 (PC1-4; N.B.: $E^0(\mathrm{PC}^{\cdot+}/^3\mathrm{PC}^*)$ for PC5 has not been reported).

Apart from TD-DFT, the oxidation potentials $E^0(\mathrm{PC}^{\cdot+}/^3\mathrm{PC}^*)$ of $^3\mathrm{PC}^*$ can also be calculated by unrestricted DFT (UDFT). The results are shown in Table S3. Out of the five density functionals, PBE0-D3BJ performs the best on average (0.15 eV in error). In view of this, PBE0-D3BJ is also selected as the density functional for calculating the energy barriers $\Delta E^{\ddagger}$ for dissociative electron transfer of the triplet exciplex $^3\mathrm{PC}^*/\mathrm{R{-}X}$.

Table S2: Calculated excited-state oxidation potentials (in eV; relative to the saturated calomel electrode) with DMF and DMAc solvents for PC1-5.

catalyst	$E^0(PC^{\cdot+}/^1PC^*)/E^0(PC^{\cdot+}/^3PC^*)$	
	DMF[a]	DMAc[b]
PC1	-2.91/-2.31	-2.91/-2.32
PC2	-2.12/-1.05	-2.12/-1.05
PC3	-1.77/-1.59	-1.77/-1.59
PC4	-2.22/-2.21	-2.22/-2.21
PC5	-2.52/-2.10	-2.53/-2.10

Note: [a]Calculated by TDDFT/PBE0-D3BJ/def2-SVP/SMD-DMF. [b]Calculated by TDDFT/PBE0-D3BJ/def2-SVP/SMD-DMAc.

Table S3: Calculated triplet-excited-state oxidation potentials (in eV; relative to the saturated calomel electrode) by UDFT for PC1-5.

catalyst	solvent	$E^0(PC^{\cdot+}/^3PC^*)$[a]				
		B3LYP[b]	PBE0[b]	M06-2X[c]	CAM-B3LYP[b]	ωB97X-D3[d]
PC1	cyclohexane	-1.73	-1.66	-1.81	-1.82	-1.78
PC2	toluene	-0.50	-0.37	-0.43	-0.45	-0.41
PC3	acetonitrile	-1.65	-1.53	-1.60	-1.66	-1.59
PC4	chloroform	-1.98	-1.88	-1.95	-2.05	-2.02
PC5	toluene	-1.68	-1.60	-1.73	-1.84	-1.85
average error[e]		0.17	0.15	0.22	0.25	0.23

Note: [a]Calculated with UPBE0-D3BJ/def2-SVP and the SMD solvation model. [b]Dispersion correction D3BJ[7] is employed. [c]Dispersion correction D3[10] is employed. [d]ωB97X-D3 is reoptimized from ωB97X-D with the D3 dispersion correction.[12] [e]Absolute difference between the calculated and experimental $E^0(PC^{\cdot+}/^3PC^*)$ being averaged over PC1-5.

S2. Energy Difference between S_1 and T_1

The adiabatic energy difference $\Delta E_{\mathrm{ST}}^{\mathrm{ad}}$ between S_1 and T_1, which equals to $-[E^0(\mathrm{PC}^{\cdot+}/{}^1\mathrm{PC}^*) - E^0(\mathrm{PC}^{\cdot+}/{}^3\mathrm{PC}^*)]$, are listed in Table S4 for PC1-5. It is interesting to notice that the $\Delta E_{\mathrm{ST}}^{\mathrm{ad}}$ for PC2 (1.06 eV) is notably larger than those for other PCs. This trend also holds for the vertical energy difference $\Delta E_{\mathrm{ST}}^{\mathrm{vert}}$ (cf. Table S4) which allows for an analysis solely at the S_0 equilibrium geometry: The transition from the highest occupied (HOMO; ψ_{H}) and lowest unoccupied (LUMO; ψ_{L}) molecular orbitals, $\psi_{\mathrm{H}} \rightarrow \psi_{\mathrm{L}}$, is the dominant configuration (contribution $> 70\%$) for both the S_1 and T_1 states of PC1-5 (cf. Table S4), which means that the repulsive interaction $2(\psi_{\mathrm{L}}\psi_{\mathrm{H}}|\psi_{\mathrm{L}}\psi_{\mathrm{H}})$ in ${}^1\mathrm{PC}^*$ but not present in ${}^3\mathrm{PC}^*$ can be a good indicator of $\Delta E_{\mathrm{ST}}^{\mathrm{vert}}$, in view of the response kernel of TD-DFT,[18] i.e.,

$$\begin{aligned}\Delta E_{\mathrm{S/T}}^{\mathrm{vert}} &= E_{\mathrm{S}}^* - E_{\mathrm{T}}^* \\ &= 2\left(\phi_{\mathrm{L}}\phi_{\mathrm{H}}\left|\frac{1}{r_{12}}\right|\phi_{\mathrm{H}}\phi_{\mathrm{L}}\right) + 2c_{\mathrm{xc}}\left(\phi_{\mathrm{L}}\phi_{\mathrm{H}}\left|(f_{\mathrm{xc}}^{\mathrm{S}} - f_{\mathrm{xc}}^{\mathrm{T}})\delta(\vec{r}_1 - \vec{r}_2)\right|\phi_{\mathrm{H}}\phi_{\mathrm{L}}\right) \\ &\approx 2\left(\phi_{\mathrm{L}}\phi_{\mathrm{H}}\left|\frac{1}{r_{12}}\right|\phi_{\mathrm{H}}\phi_{\mathrm{L}}\right),\end{aligned} \tag{S3}$$

where E_{S}^* and E_{T}^* are the vertical excitation energies for S_1 and T_1, respectively, c_{xc} is the scaling coefficient denoting the portion of the static exchange-correlation potential included in the chosen hybrid functional, and $f_{\mathrm{xc}}^{\mathrm{S}} - f_{\mathrm{xc}}^{\mathrm{T}}$ is the difference between the exchange-correlation kernels for S_1 and T_1, being a small term. As such, it is not surprising to see that larger $2(\psi_{\mathrm{L}}\psi_{\mathrm{H}}|\psi_{\mathrm{L}}\psi_{\mathrm{H}})$ corresponds to larger $\Delta E_{\mathrm{ST}}^{\mathrm{vert}}$ and $\Delta E_{\mathrm{ST}}^{\mathrm{ad}}$ (cf. Table S4). That is, PC2's largest $\Delta E_{\mathrm{ST}}^{\mathrm{ad}} = 1.06$ eV is attributed to its most significant $2(\psi_{\mathrm{L}}\psi_{\mathrm{H}}|\psi_{\mathrm{L}}\psi_{\mathrm{H}}) = 5.8 \times 10^{-3}$.

Table S4: Adiabatic and vertical energy differences between S_1 and T_1, dominant configurations of both excitations, and $2(\psi_L\psi_H|\psi_L\psi_H)$ for PC1-5.

catalyst	$\Delta E_{ST}^{ad\,a}$	$\Delta E_{ST}^{vert\,b}$	$\phi_H \rightarrow \phi_L$ in S_1/T_1 ^{bc}	$2(\psi_L\psi_H\|\psi_L\psi_H)^b$
PC1	0.59 eV	0.56 eV	95%/78%	1.9×10^{-3}
PC2	1.06 eV	1.21 eV	97%/95%	5.8×10^{-3}
PC3	0.18 eV	0.27 eV	96%/95%	1.3×10^{-3}
PC4	0.01 eV	0.01 eV	93%/92%	4.2×10^{-5}
PC5	0.42 eV	0.42 eV	95%/81%	1.7×10^{-3}

Note: The results are calculated by TDDFT/PBE0-D3BJ/def2-SVP/SMD-DMF. [a]Adiabatic energy difference between S_1 and T_1, equaling to $-(E^0(PC^{\cdot+}/^1PC^*) - E^0(PC^{\cdot+}/^3PC^*))$. [b]Calculated based on the equilibrium geometry of S_0. [c]The contribution of the $\phi_H \rightarrow \phi_L$ configuration of S_1 and T_1 excitations, respectively.

S3. Electronic Structure of Lowest Excited States

Clearly, the binding strength $\Delta E_{\mathrm{b}}^{\mathrm{ex}}$ of the exciplex ^{1,3}PC*/R−X (between ^{1,3}PC* and R−X) is dependent on the electronic structure of ^{1,3}PC*. To see this, the excitation types of ^{1,3}PC* for PC1-5 are documented in Fig. S1. The ^{3}PC* of PC1 and ^{1}PC* of PC2 are all local excitations, with positively charged regions only exist in the vicinity of peripheral phenyl-H atoms (cf. Fig. S2). By contrast, the ^{3}PC* of PC3, PC4, and PC5 are all charge-transfer type of excitations (cf. Fig. S1), which renders notable charge separation with half of the molecule positively charged and the other half negatively charged (cf. Fig. S2). Since the charge-separated ^{3}PC* and R−X are both polar systems, higher $\Delta E_{\mathrm{b}}^{\mathrm{ex}}$ of ^{3}PC*/R−X for PC3-5 can be expected (cf. Table 1 in the main text).

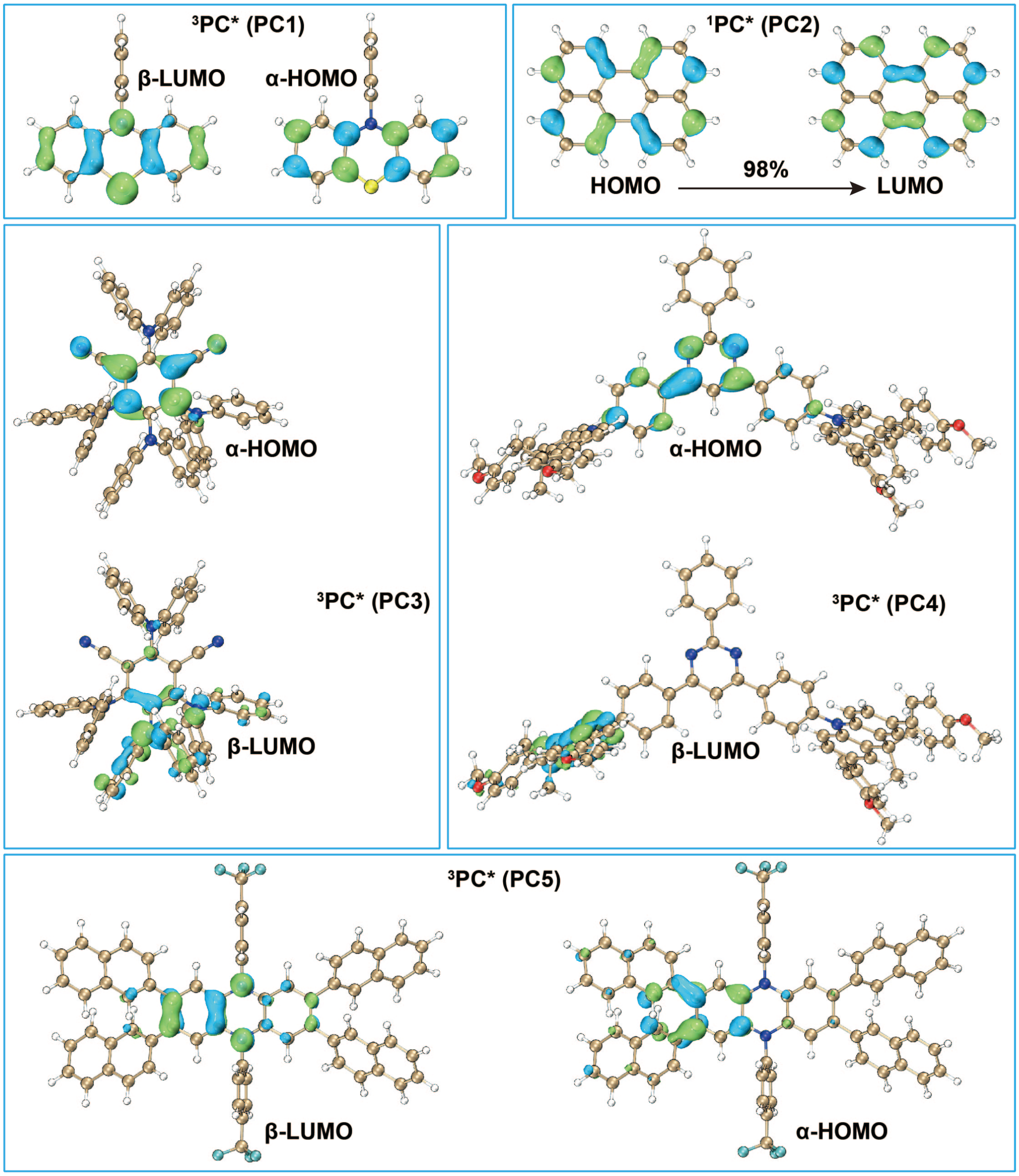

Figure S1: Illustration of the electron and hole of ^{3}PC* (represented by the α-HOMO and β-LUMO, respectively) for PC1, PC3, PC4, and PC5, as well as ^{1}PC* (represented by the LUMO and HOMO, respectively, in the case of HOMO$\rightarrow$LUMO-dominated S_1 excitation) for PC2. ^{3}PC* and ^{1}PC* are calculated by UPBE0/def2-SVP/SMD-DMF and TDDFT/PBE0/def2-SVP/SMD-DMF, respectively.

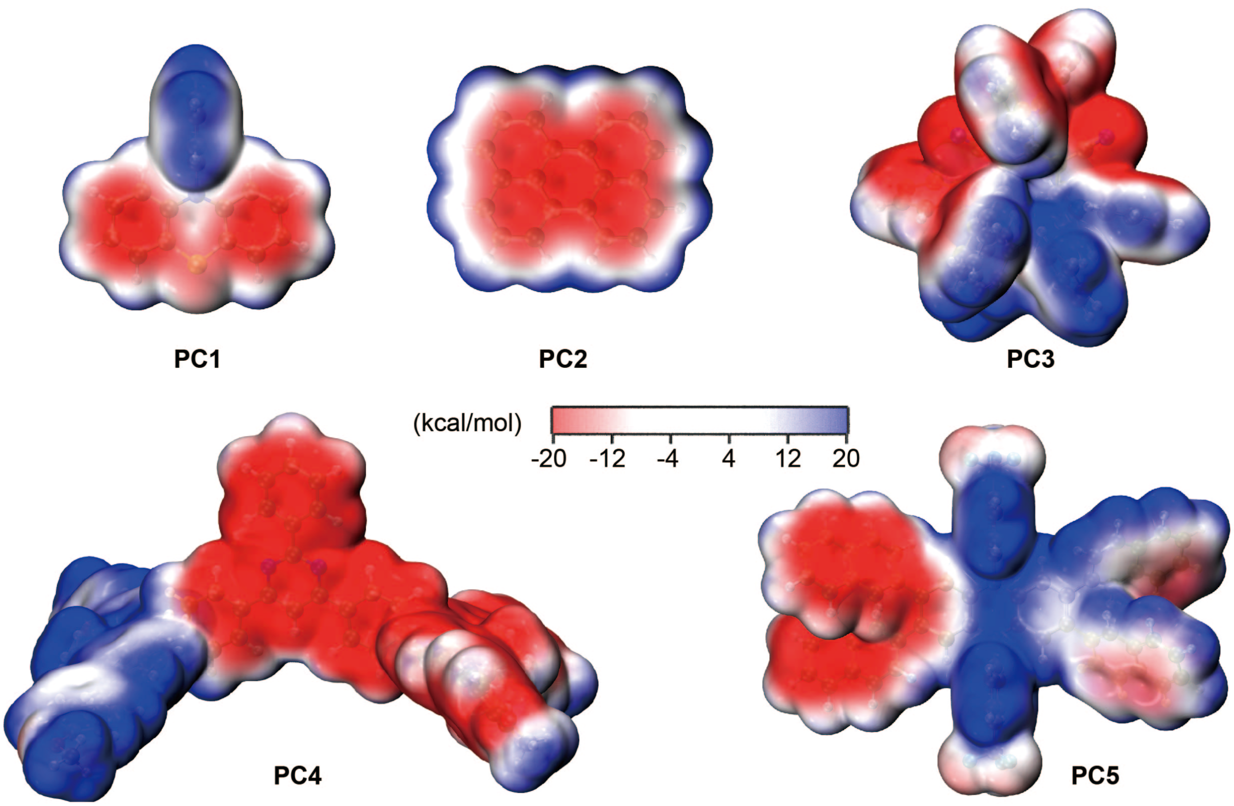

Figure S2: ^{3}PC* (PC1, PC3, PC4, and PC5) and ^{1}PC* (PC2) electrostatic potential maps[19,20] on the van Der Waals surfaces at their respective equilibrium geometries, calculated by UPBE0/def2-SVP/SMD-DMF and TDDFT/PBE0/def2-SVP/SMD-DMF, respectively.

S4. Fragment Localized Molecular Orbitals

In the main text, the dissociative electron transfer ^{1,3}PC*/R−X $\longrightarrow$ PC$^{\cdot+}$/X$^-$ + R$^\cdot$ in O-ATRP is studied in detail with PC1 as an example, where fragment localized molecular orbitals (FLMO)[21] are used to reveal the contributions of individual orbitals to the electron/hole transfer process. The FLMOs are constructed noniteratively in terms of the orthonormal primitive FLMOs from subsystem calculations[22,23] (phenyl, Br, and acetyl for EBPA; N-phenyl and phenothiazine for PC1) by using the BDF program package.[24,25] In particular, the localized FLMOs for a conjugated π system can be canonicalized to produce the same number of regional FLMOs (delocalized within the π system). That is, the six phenyl-π/π^* and fourteen phenothiazine-π/π^* localized FLMOs are converted to six (see Fig. S3) and fourteen (see Fig. S4) regional FLMOs, respectively. The so-obtained new FLMOs $\phi^{\mathrm{FLMO}}_{\mathrm{R}_0,p}$, still being orthonormal, can be used to expand the occupied spin orbitals $\{\phi^{\mathrm{FLMO}}_{\mathrm{R},i\sigma}\}$ of ^{3}PC*/R−X with coefficients $C^{\sigma}_{pi}(\mathrm{R})$. The coefficients for the six phenyl-π/π^* (and fourteen phenothiazine-π/π^* as well) are summed up for analysis (i.e., π/π^* orbitals of a conjugated system are discussed as a whole), in order to simplify the discussion.

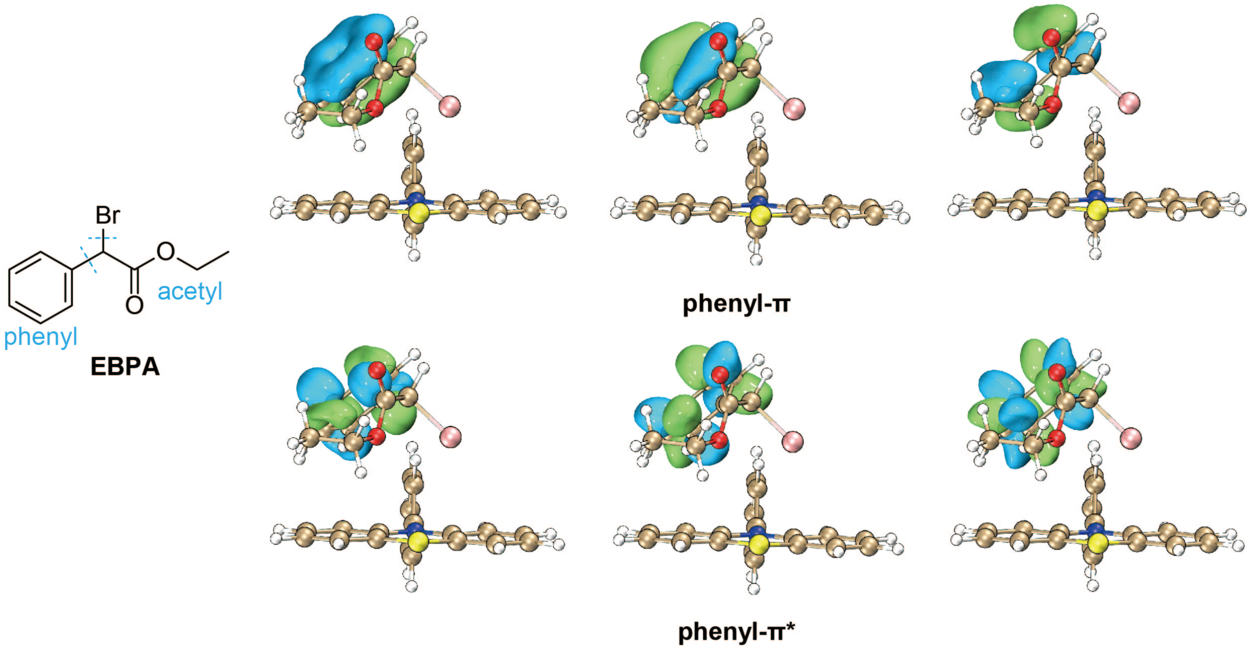

Figure S3: Ground-state (S_0) regional FLMOs corresponding to the six phenyl-π/π^* of PC/R−X but at the equilibrium geometry of ^{3}PC*/R−X, calculated by PBE0/def2-SVP.

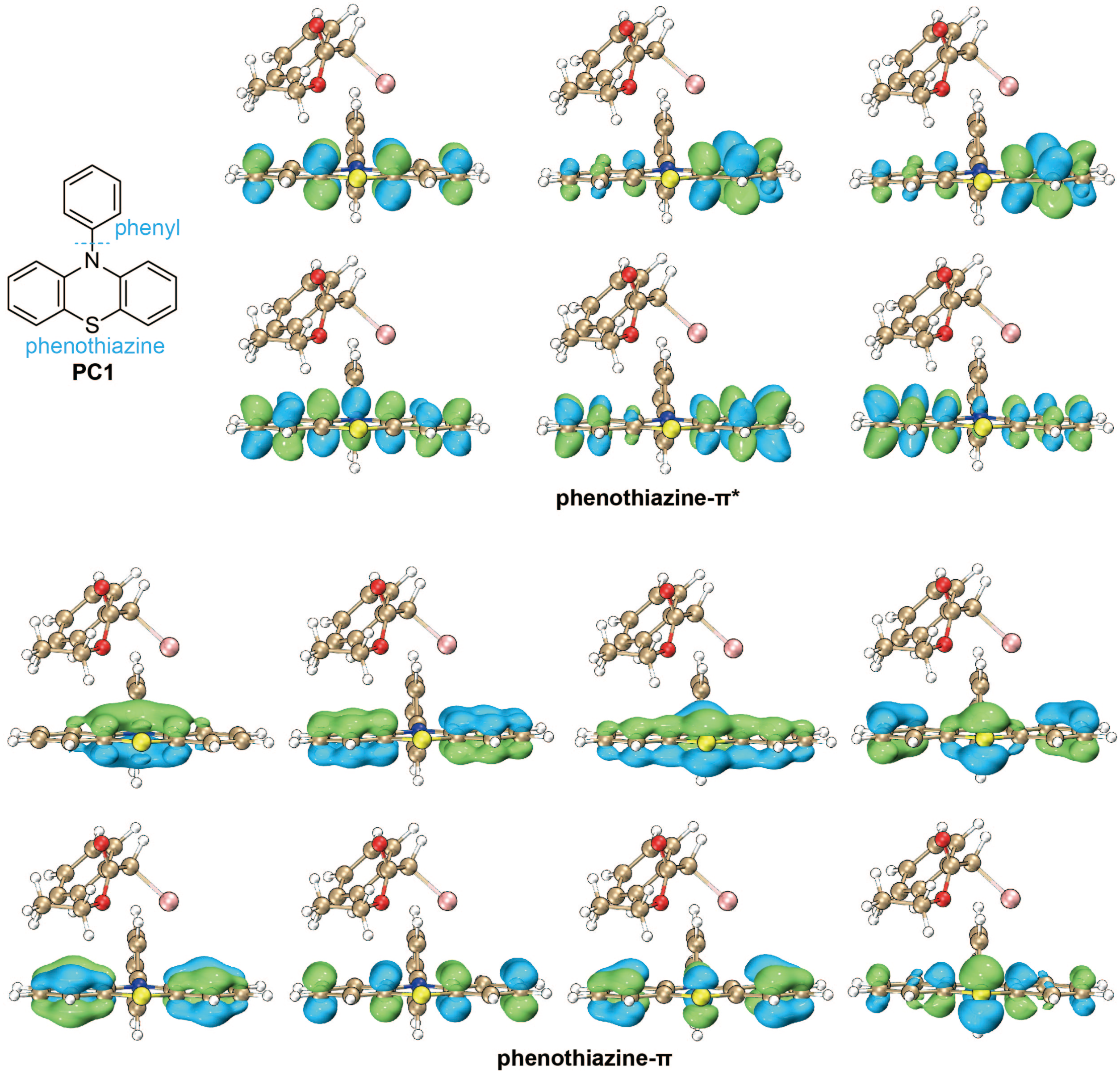

Figure S4: Ground-state (S_0) regional FLMOs corresponding to the fourteen phenothiazine-π/π^* of PC/R−X but at the equilibrium geometry of ^{3}PC*/R−X, calculated by PBE0/def2-SVP.

S5. Exciplexes for Activation and Reactivation

From discussions in the main text, it is noticed that the initiator R−X, which can bind with ^{1,3}PC* to form an exciplex ^{1,3}PC*/R−X, becomes a polymer chain P_n−X during the polymerization, so that the exciplex becomes ^{1,3}PC*/P_n−X. The question is then whether ^{1,3}PC*/R−X and ^{1,3}PC*/P_n−X have similar binding strengths $\Delta E_{\mathrm{b}}^{\mathrm{ex}}$. To see this, chemical structures of R−X and P_n−X (cf. Fig. S5a) are checked first, which both contain the ethyl-α-bromo-acetate core structure but differ in that the latter has a more bulky α-substituent than the former. Owing to such similarity, R−X and P_n−X can bind with the ^{3}PC* of PC1 in a similar way (see Fig. S5b and c, respectively) and thus result in $\Delta E_{\mathrm{b}}^{\mathrm{ex}}$ of 11.2 kcal/mol and 11.6 kcal/mol, respectively, which do not differ significantly from each other.

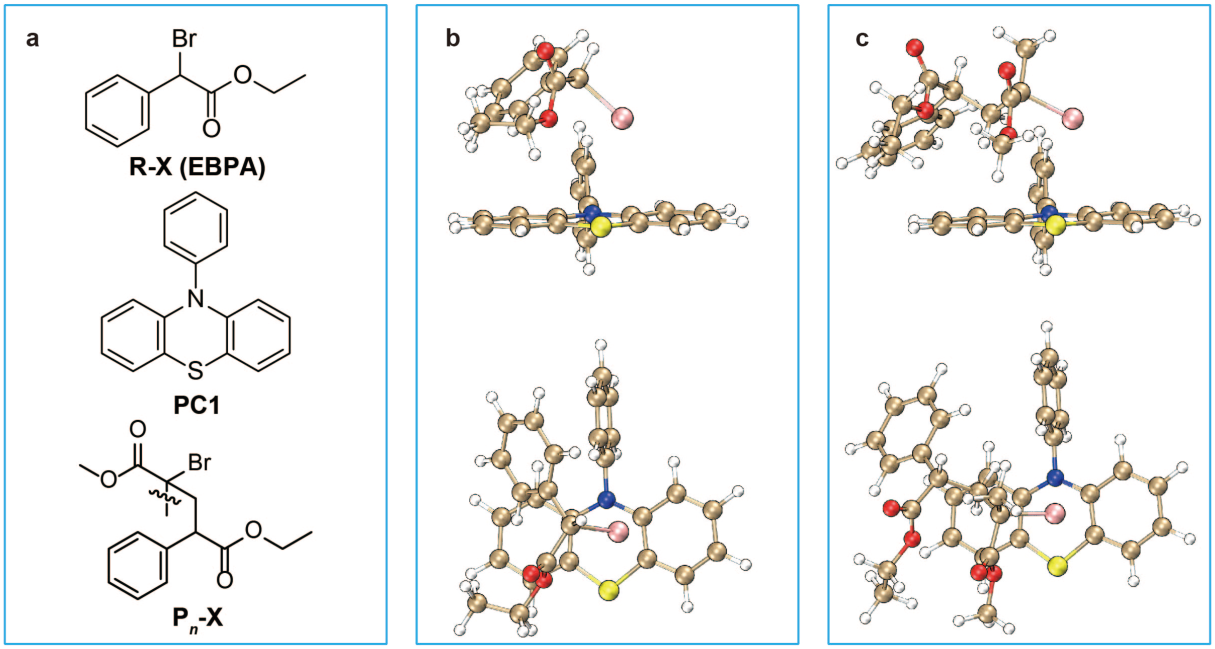

Figure S5: (a) Chemical structures of R−X (EBPA), PC1, and P_n−X. (b-c) Equilibrium geometries of the exciplexes ^{3}PC*/R−X (b) and ^{3}PC*/P_n−X (c) for PC1, calculated by UPBE0-D3BJ/def2-SVP/SMD-DMF.

S6. Maximum Initiator Efficiency, Minimum Catalyst Loading and Minumum Molecular weight Dispersity

The 'maximum initiator efficiency' (MIE) in O-ATRP is defined as the highest initiator efficiency I^* (best to be $\approx$ 100%) that is possible with a specific PC. On the other hand, the 'minimum catalyst loading' (MCL) is defined as the lowest catalyst loading of a PC being sufficient for achieving satisfactory polymerization control (i.e., high I^* and low $Đ$; ideally, $I^* \approx 100\%$ and as low $Đ$ as possible). The 'minimum molecular weight dispersity' (MMD) in O-ATRP is defined as the lowest achievable molecular weight dispersity $Đ$ for a particular PC. To determine properly the MIE, MCL and MMD for different PCs, standard O-ATRP conditions are used, where the molar ratio between the monomer and R−X, [M]:[R−X], should be around 100:1∼200:1, and monomer conversions should be $\geq$ 50%.

The so-determined MIE for O-ATRP with PC1 (cf. Nos. 1-3 of Table S5), PC4 (cf. Nos. 12-13 of Table S5), and PC5 (cf. Nos. 15-18 of Table S5) turn out to be $\approx$ 100%, whereas the MCL for PC1 (cf. No. 1 of Table S5), PC4 (cf. No. 12 of Table S5), and PC5 (cf. Nos. 17 of Table S5) are 1000 ppm, 50 ppm, and 10 ppm, respectively. As for PC3, it is clear from Nos. 9-11 of Table S5 that the I^* cannot exceed 61% even if the catalyst loading is increased from 0.5 ppm to 100 ppm, indicating an MIE of 61%. Since the best polymerization control with PC3 ($I^* = 61\%$ and $Đ = 1.30$) being achieved at 10 ppm loading (cf. No. 10 of Table S5) is only slightly better than that ($I^* = 53\%$ and Đ = 1.39; cf. No. 11 of Table S5) at 0.5 ppm, the MCL for PC3 is then 0.5 $\sim$ 10 ppm. Lastly, as seen in Nos. 4-7 of Table S5, with the loading of PC2 being increased from 1 ppm to 1143 ppm, the I^* is improved from 1% to 14% yet still being very low, meaning that the MCL for PC2 must be $\gg$ 1000 ppm. Due to solubility issues, a catalyst loading much higher than 1143 ppm is not possible with PC2, which means that the exact MCL for PC2 cannot be determined. On the other hand, $I^* = 88\%$ (cf. No. 8 in Table S5) is found possible with PC2 by reducing the loading of R−X by nine times (i.e., from [M]:[R−X] = 875:9 to [R−X] = 875:1), which can be regarded

(in terms of the [R−X]:[PC2] ratio) as increasing the loading of PC2 by nine times. It can then be deduced that the MIE for O-ATRP with PC2 is around 88% and the MCL for PC2 could be up to nine times higher than 1143 nm (i.e., $\gg$ 1000 ppm).

Table S5: Polymerization performances of PC1-5 in O-ATRP.

No.	catalyst	loading^a	R−X	monomer	[M]:[R−X]^b	$M_{n,exp}$^{c}	$M_{n,theo}$^{d}	I^{*e}	$Đ^f$	Ref.
1^g	PC1	1000 ppm	EBPA	MMA	100:1	6200	7200	116%	1.30	26
2^g	PC1	1000 ppm	EBPA	BMA	100:1	12000	11000	92%	1.25	26
3^g	PC1	1000 ppm	EBPA	BMA	100:1	15400	14000	91%	1.32	26
4^h	PC2	1143 ppm	EBPA	MMA	875:9	41200	5760	14%	1.29	14
5^h	PC2	114 ppm	EBPA	MMA	875:9	125000	6200	5%	1.39	14
6^h	PC2	11 ppm	EBPA	MMA	875:9	253000	5200	2%	1.46	14
7^h	PC2	1 ppm	EBPA	MMA	875:9	273000	2750	1%	1.57	14
8^h	PC2	1143 ppm	EBPA	MMA	875:1	55800	48900	88%	1.65	14
9^h	PC3	100 ppm	DBMM	MMA	200:1	17900	10100	56%	1.56	15
10^h	PC3	10 ppm	DBMM	MMA	200:1	19300	11800	61%	1.30	15
11^h	PC3	0.5 ppm	DBMM	MMA	200:1	23300	12200	53%	1.39	15
12^g	PC4	50 ppm	DBMM	MMA	200:1	12200	13900	114%	1.27	16
13^g	PC4	25 ppm	DBMM	MMA	200:1	13200	14500	110%	1.58	16
14^g	PC4	10 ppm	DBMM	MMA	200:1	14700	11500	78%	1.81	16
15^g	PC5	100 ppm	DBMM	MMA	100:1	9500^j	10200^j	93%	1.09	17
16^g	PC5	50 ppm	DBMM	MMA	100:1	7800^j	8200^j	106%	1.14	17
17^g	PC5	10 ppm	DBMM	MMA	100:1	8900^j	8200^j	92%	1.27	17
18^g	PC5	5 ppm	DBMM	MMA	100:1	7400^j	7600^j	103%	1.42	17

Note: ^{a}Catalyst loading with respect to the monomer. ^{b}Molar ratio between the monomer and R−X. ^{c}Experimentally determined number-averaged molecular weight. ^{d}Theoretical number-averaged molecular weight derived from monomer conversions. ^{e}Initiator efficiency, defined as $M_{n,exp}/M_{n,theo}$. ^{f}Molecular weight dispersity, defined as the ratio between the weight-averaged and number-averaged molecular weights. ^{g}Performed in DMAc as the solvent. ^{h}Performed in DMF as the solvent. ^{i}Performed without an additional solvent (i.e., neat monomers). ^{j}Derived based on the weight-averaged molecular weight, $Đ$, and I^*.
DMAc: N,N-dimethyl acetamide. DMF: N,N-dimethyl formamide. DMSO: dimethyl sulfoxide. EBPA: ethyl-α-bromophenylacetate. DBMM: diethyl 2-bromo-2-methylmalonate. MMA: methyl methacrylate. BMA: benzyl methacrylate. BA: benzyl acrylate.

S7. Potential Side Reaction for $PC^{\cdot+}$

From discussions in the main text, the unpaired electron in $PC^{\cdot+}$ resides mainly on S and N, such that a side reaction may occur between S and $P_n^{\cdot}$ (NB: N is blocked by phenyl-H and is hence not reactive, see Fig. S6a). To confirm this, the distance between the S of $PC^{\cdot+}$ and the sp^2 alkyl-C of $P_n^{\cdot}$ is shortened from a value of 3.5 Å to the equilibrium (2.0 Å) in a relaxed scan, which does reveal a monotonically decreasing energy profile (see Fig. S6b), so as to lead to an unwanted product $PC{-}P_n^{+}$.

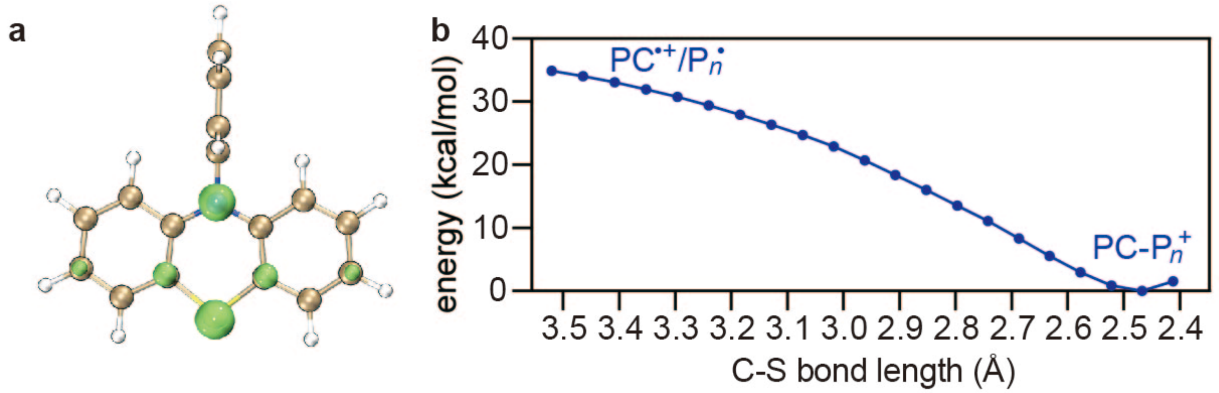

Figure S6: Net Mulliken spin populations of $PC^{\cdot+}$ (a) as well as relaxed scan of the C−S bond between the sp^2 alkyl-C of $P_n^{\cdot}$ and the S of $PC^{\cdot+}$ (b), using UPBE0-D3BJ/def2-SVP/SMD-DMF.

S8. Validity of New Descriptors for PCs within the Same Family

In the main text, we have examined PCs bear very different chromophore cores and substituents. To broden the validation scope of our proposed descriptors, we also tested PCs bearing the same chromophore core and similar structures. The most common PC families employed for OQP in O-ATRP encompass dihydrophenazines,[17,27,28] phenothiazines,[29] phenoxazines[30] and acridines.[31] We have selected core-substituted diaryl dihydrophenazines (see Fig. S7) as newly added model systems because these PCs possess following characteristics: (i) similar chemical structures with the same chromophore core; (ii) the ability to mitigate side reactions associated with alkyl core substitution (AkCS) [17-19]; (iii) a wealth of distinguishable experimental results adopting different catalyst loadings, which are ideal for determining the performance descriptors MIE, MCL and MMD. Whereas the reasons for omitting other PC families are as follows: (i) non-core-substituted diaryl dihydrophenazines suffer AkCS effect;[17,27,28] (ii) non-core-substituted diaryl dihydrophenazines, phenothiazines and phenoxazines are mostly reported with fixed catalyst loadings (e.g. 1000 ppm). The relatively limited experimental data makes it challenging to determine the performance descriptors (MIE, CUD and MMD); (iii) dimethyl acridines, on the other hand, are mostly reported with n-butyl acrylate (BA) as the monomer, making the experimental results less comparable with those adopting methyl methacrylate (MMA).

In the main text, we have identified three performance descriptors (MIE, MCL, and MMD). To gain the MIE, MCL and MMD, the performances for PCs 5-11 are summarized in Table S6. From the experimental date, we can conclude that the MIE for O-ATRP with PC5 (cf. Nos. 1-4 of Table S6), PC6 (cf. Nos. 5-8 of Table S6), PC7 (cf. Nos. 9-10 of Table S6), PC8 (cf. No. 11 of Table S6), PC9 (cf. Nos. 12-15 of Table S6), PC10 (cf. Nos. 16-19 of Table S6), and PC11 (cf. No. 20 of Table S6) turn out to be $\approx 100\%$. The MCL for PC5 (cf. Nos. 1-4 of Table S6), PC6 (cf. Nos. 5-8 of Table S6), PC9 (cf. Nos. 12-15 of Table

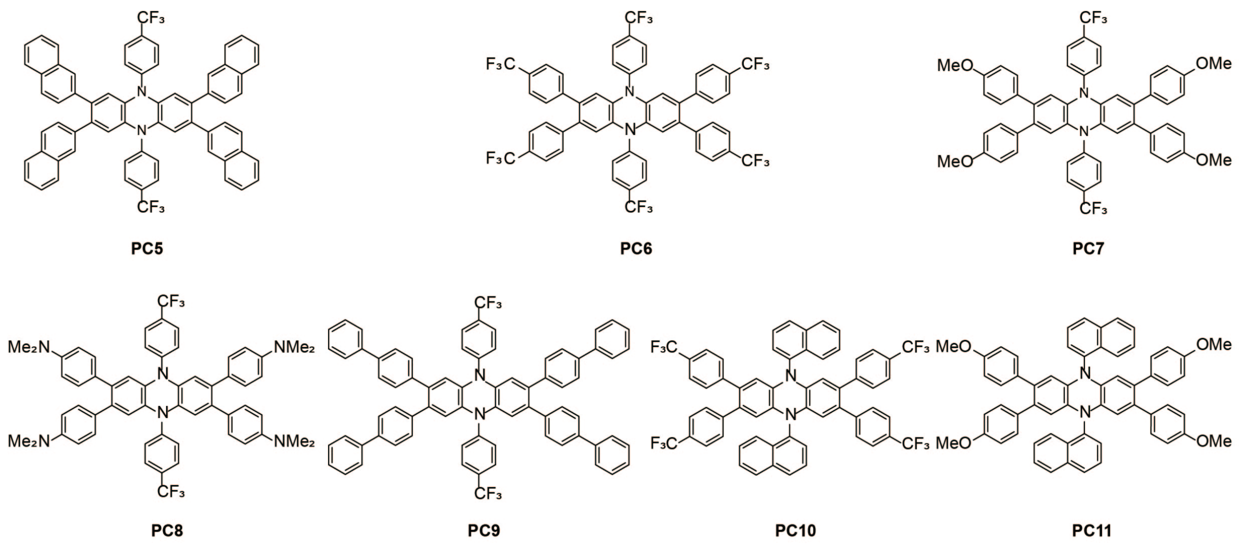

Figure S7: Chemical structures of core-substituted diaryl dihydrophenazines (PC5-11)

S6), PC10 (cf. Nos. 16-19 of Table S6) are around 50 ppm, whereas for PC7 (cf. Nos. 9-10 of Table S6), PC8 (cf. No. 11 of Table S6), and PC11 (cf. No. 20 of Table S6), it is hard to give precise MCLs for lacking of more detailed controlled experiments. However, based on the existing experimental data,[17,28] we can conclude qualitatively that the MCLs are higher than 100 ppm. The MMD for PC7 (cf. Nos. 9-10 of Table S6), PC8 (cf. No. 11 of Table S6), and PC11 (cf. No. 20 of Table S6) are higher than that for PC5 (cf. Nos. 1-4 of Table S6), PC6 (cf. Nos. 5-8 of Table S6), PC9 (cf. Nos. 12-15 of Table S6) and PC10 (cf. Nos. 16-19 of Table S6), indicating less control for polymerization.

In the main text, we have identified three pairs of property-performance descriptors (AED-MIE, CUD-MCL, and DED-MMD). To validate these descriptors pairs, we have included corresponding results for PCs 5-11 in Table S7. By carefully analyzing these results, the following conclusions can be drawn: (i) Generally, the property descriptors all well align with relevant performance descriptors in the expected manner. (ii) Specifically, the AED values for PC5-11 are all exceeded than that PC3 at a value of 9.6×10^1. This implies a more rapid conversion of R-X initiators to polymer chains, thereby rendering a higher MIE. This observation aligns perfectly with the reported MIE values, wherein PC5-11 yield a MIE of approximately to 100%, higher than that PC3 at a value of 61%. (iii) A lower MCL

Table S6: Polymerization performances of PCs 5-11 in O-ATRP.

No.	catalyst	loading[a]	R−X	monomer	[M]:[R−X][b]	$M_{n,exp}$[c]	$M_{n,theo}$[d]	I^{*e}	$Đ^f$	Ref.
1[g]	PC5	100 ppm	DBMM	MMA	100:1	9500[j]	10200[j]	93%	1.09	17
2[g]	PC5	50 ppm	DBMM	MMA	100:1	7800[j]	8200[j]	106%	1.14	17
3[g]	PC5	10 ppm	DBMM	MMA	100:1	8900[j]	8200[j]	92%	1.27	17
4[g]	PC5	5 ppm	DBMM	MMA	100:1	7400[j]	7600[j]	103%	1.42	17
5[g]	PC6	500 ppm	DBMM	MMA	100:1	7180[j]	7010[j]	98%	1.18	17
6[g]	PC6	100 ppm	DBMM	MMA	100:1	7720[j]	7640[j]	99%	1.14	17
7[g]	PC6	50 ppm	DBMM	MMA	100:1	9560[j]	9040[j]	95%	1.14	17
8[g]	PC6	10 ppm	DBMM	MMA	100:1	8000[j]	7620[j]	95%	1.50	17
9[g]	PC7	1000 ppm	DBMM	MMA	100:1	4500[j]	4730[j]	105%	1.34	17
10[g]	PC7	100 ppm	DBMM	MMA	100:1	8520[j]	7960[j]	93%	1.49	17
11[g]	PC8	1000 ppm	DBMM	MMA	100:1	7470[j]	9640[j]	129%	1.82	17
12[g]	PC9	500 ppm	DBMM	MMA	100:1	6900[j]	8140[j]	118%	1.12	17
13[g]	PC9	100 ppm	DBMM	MMA	100:1	6620[j]	6480[j]	98%	1.13	17
14[g]	PC9	50 ppm	DBMM	MMA	100:1	8940[j]	9560[j]	107%	1.13	17
15[g]	PC9	10 ppm	DBMM	MMA	100:1	7830[j]	7550[j]	96%	1.29	17
16[g]	PC10	500 ppm	DBMM	MMA	100:1	7530[j]	7030[j]	93%	1.07	28
17[g]	PC10	100 ppm	DBMM	MMA	100:1	7710[j]	6710[j]	87%	1.16	28
18[g]	PC10	50 ppm	DBMM	MMA	100:1	8800[j]	8090[j]	92%	1.07	28
19[g]	PC10	10 ppm	DBMM	MMA	100:1	7170[j]	6940[j]	97%	1.49	28
20[g]	PC11	1000 ppm	DBMM	MMA	100:1	5320[j]	5370[j]	101%	1.57	28

Note: [a]Catalyst loading with respect to the monomer. [b]Molar ratio between the monomer and R−X. [c]Experimentally determined number-averaged molecular weight. [d]Theoretical number-averaged molecular weight derived from monomer conversions. [e]Initiator efficiency, defined as $M_{n,exp}/M_{n,theo}$. [f]Molecular weight dispersity, defined as the ratio between the weight-averaged and number-averaged molecular weights. [g]Performed in DMAc as the solvent. DMAc: N,N-dimethyl acetamide. DBMM: diethyl 2-bromo-2-methylmalonate. MMA: methyl methacrylate.

must be achieved with a higher CUD. Indeed, the MCL values for PC7, PC8 and PC11 with lower CUD values are higher than those for PC5, PC6, PC9 and PC10 with high CUD values. Thus, the CUD results exhibit a strong correlation with MCL for PC5-11. (vi) When comparing the DED values, it becomes apparent that when the DED is sufficiently low (as seen in PC7, PC8 and PC11), the MMD will tends to be significantly high, signifying a less efficient alternating growth of polymer chains. Thus, the DED values align well with MMD.

In short, the newly added PC6-11 of the core-substituted diaryl dihydrophenazines also well verify the validity of the three pairs of property-performance descriptors established in this work.

Table S7: Performances of catalysts in O-ATRP and calculated molecular descriptors

No.	catalyst	polymerisation[a] loading ppm	I^{*c}	$Đ$	descriptor[b] ΔE_b^{ex} kcal/mol	τ_b^{ex} ns	$\Delta E^{\ddagger}$ kcal/mol	$\tau^{\ddagger}$ ns	$\Delta E_b^{PC\text{-}X}$ kcal/mol	$\tau_b^{PC\text{-}X}$ ns	$\Delta E_{de}^{\ddagger}$ kcal/mol	$\tau_{de}^{\ddagger}$ ns	AED	CUD ns^2	DED
5a	PC5[17]	50	106%	1.14	17.5	7.6×10^{8}	3.3	2.9×10^{-2}	17.4	6.3×10^{8}	3.9	8.5×10^{-2}	2.6×10^{10}	4.9×10^{17}	3.1×10^{-1}
5b		10	92%	1.27											
6a	PC6[17]	50	95%	1.14	17.4	6.5×10^{8}	4.3	1.6×10^{-1}	18.2	2.5×10^{9}	3.8	6.8×10^{-2}	4.1×10^{9}	1.6×10^{18}	9.0×10^{0}
6b		10	95%	1.50											
7a	PC7[17]	1000	105%	1.34	17.2	4.6×10^{8}	1.5	1.4×10^{-3}	16.8	2.3×10^{8}	4.6	2.6×10^{-1}	3.3×10^{11}	1.1×10^{17}	2.7×10^{-3}
7b		100	93%	1.49											
8	PC8[17]	1000	129%	1.82	17.1	3.9×10^{8}	0.9	5.1×10^{-4}	15.7	3.7×10^{7}	5.4	1.0×10^{0}	7.6×10^{11}	1.4×10^{16}	4.7×10^{-5}
9a	PC9[17]	50	107%	1.13	17.4	6.5×10^{8}	3.0	1.8×10^{-2}	17.4	6.5×10^{8}	4.3	1.6×10^{-1}	3.7×10^{10}	4.2×10^{17}	1.1×10^{-1}
9b		10	96%	1.29											
10a	PC10[27]	50	92%	1.07	17.4	6.5×10^{8}	3.1	2.1×10^{-2}	18.0	1.6×10^{9}	4.1	1.1×10^{-1}	3.1×10^{10}	1.2×10^{18}	5.1×10^{-1}
10b		10	97%	1.49											
11	PC11[27]	1000	101%	1.57	16.6	1.7×10^{8}	1.1	7.2×10^{-4}	16.6	1.7×10^{8}	5.0	5.2×10^{-1}	2.3×10^{11}	2.8×10^{16}	1.4×10^{-3}

Note: [a]Results with diethyl-2-bromo-2-methylmalonate initiator solvated in DMAc, methyl methacrylates as the monomer, and a formulation of [monomer]:[initiator] = 100:1. [b]$\Delta E^{\ddagger}$ calculated with (LOSC-) PBE0-D3BJ/def2-SVP/SMD-DMF for PC5-11; ΔE_b^{ex}, $\Delta E_b^{PC\text{-}X}$ and $\Delta E_{de}^{\ddagger}$ calculated with UPBE0-D3BJ/def2-SVP/SMD-DMF for PC5-11. [c]the value of $I^* \gg 100\%$ indicates autoinitiation or the occurrence of other side reactions.

Supplementary Appendix A: Minimum Energy Crossing Point

The geometry for the minimum energy crossing point between S_1 and T_2 of PC5 is optimized by PBE0-D3BJ/def2-SVP, at which the spin orbit coupling matrix element between S_1 and T_2 is calculated by sf-X2C-TDDFT-SOC[32]/PBE0-D3BJ/X2C-SVPall using the BDF[24,25] software package.

Coordinates (Å):

C, 1.89326648, -2.92199012, 0.94192309

C, 2.19103626, -1.63655047, 0.48655190

C, 1.23061011, -0.81642647, -0.11481242

C, -0.08540284, -1.31534896, -0.30174600

C, -0.39988529, -2.58736336, 0.20857547

C, 0.54109175, -3.38240771, 0.85808881

C, -0.71036307, 0.72349886, -1.41353227

C, 0.59166545, 1.24635132, -1.19740592

C, 0.87701415, 2.55328704, -1.61145627

C, -0.06354454, 3.34947757, -2.26535405

C, -1.33730297, 2.78296810, -2.58632992

C, -1.63958602, 1.50496651, -2.12173655

N, -1.01969868, -0.53726378, -0.95030163

C, -2.33277843, -1.04578022, -1.14603979

C, -3.35680136, -0.71503052, -0.22911650

C, -2.60987977, -1.87470374, -2.25613281

C, -4.62341404, -1.20189323, -0.42225242

C, -3.87762830, -2.36039489, -2.44176341

C, -4.91645352, -2.03814316, -1.53529629

H, 3.21881989, -1.28111841, 0.54432585

H, -1.43189622, -2.92943236, 0.14366136

H, 1.84718115, 2.97958125, -1.36011024

H, -2.60568462, 1.06510468, -2.36535142

H, -3.11072497, -0.07429748, 0.61497121

H, -1.79860457, -2.10591304, -2.94330949

H, -5.42541360, -0.95635504, 0.27250586

H, -4.11441896, -2.99693785, -3.29117033

N, 1.53147206, 0.46015180, -0.55653252

C, 2.84399010, 0.97458267, -0.34952593

C, 3.79368022, 0.87757676, -1.37289686

C, 3.16621454, 1.57523033, 0.87458567

C, 5.06824397, 1.38091636, -1.17092480

H, 3.51234436, 0.40637208, -2.31501798

C, 4.44132873, 2.07668263, 1.07172615

H, 2.40367540, 1.64158658, 1.65120951

C, 5.40067637, 1.98242072, 0.05083606

H, 5.81925730, 1.31725041, -1.95813637

H, 4.70496833, 2.55673933, 2.01492210

C, -6.27643060, -2.55734687, -1.70680094

C, 6.78456286, 2.48684971, 0.29800705

F, -6.63327196, -3.39805779, -0.70768981

F, -6.44063329, -3.23496972, -2.85356823

F, -7.20277861, -1.57289099, -1.69894124

F, 6.77522930, 3.64559355, 0.96911409

F, 7.45971908, 2.68447858, -0.83860984

F, 7.49959505, 1.62169536, 1.03454625

C, 3.01364909, -3.75761495, 1.42997197

C, 3.08432585, -5.11098119, 1.14198688

C, 4.05995857, -3.17437646, 2.19887673

C, 4.14558926, -5.91775225, 1.61414691

H, 2.30851088, -5.58393217, 0.53695941

C, 5.10660394, -3.93083954, 2.66643391

H, 4.01100262, -2.11259871, 2.45027358

C, 5.18258955, -5.32132472, 2.39611784

C, 4.20809652, -7.31008333, 1.34055746

H, 5.89055872, -3.46535586, 3.26972225

C, 6.24001386, -6.13647744, 2.87307562

C, 5.24757670, -8.07432805, 1.81637624

H, 3.40894316, -7.76506770, 0.75019447

C, 6.27410500, -7.48261433, 2.58929899

H, 7.02962954, -5.67535210, 3.47162420

H, 5.28238542, -9.14483612, 1.60088531

H, 7.09495192, -8.10076094, 2.96176438

C, 0.06955377, -4.64316016, 1.48100451

C, 0.55157013, -5.06642954, 2.70785040

C, -0.91062088, -5.44056055, 0.82606029

C, 0.11687312, -6.27491203, 3.30125659

H, 1.28845073, -4.46637964, 3.24523060

C, -1.35696145, -6.61558726, 1.38045139

H, -1.28907444, -5.12992274, -0.14991177

C, -0.85765827, -7.07301690, 2.62645133

C, 0.62864082, -6.72517573, 4.54715122

H, -2.09781704, -7.22167695, 0.85212310

C, -1.28343433, -8.28790845, 3.22193991

C, 0.19542221, -7.90750737, 5.09949942

H, 1.38083840, -6.11712968, 5.05594482

C, -0.77055367, -8.69649005, 4.43135879

H, -2.02810428, -8.89495457, 2.70110100

H, 0.59911790, -8.24402948, 6.05729603

H, -1.10678342, -9.63446228, 4.88080205

C, -2.34017736, 3.46082380, -3.44333183

C, -1.96071014, 4.21142814, -4.54319607

C, -3.72807363, 3.33514126, -3.15467536

C, -2.91108583, 4.87160856, -5.35703072

H, -0.90508923, 4.30955057, -4.80360382

C, -4.67472919, 3.95885440, -3.93060178

H, -4.04378792, 2.76090899, -2.28131676

C, -4.30086114, 4.74803581, -5.04816988

C, -2.52386318, 5.66377121, -6.47026721

H, -5.73480350, 3.86304185, -3.68085820

C, -5.24868553, 5.41855800, -5.86262770

C, -3.46680990, 6.30120615, -7.24194633

H, -1.45970155, 5.76415185, -6.69797413

C, -4.84258949, 6.17711159, -6.93635880

H, -6.31011171, 5.32150651, -5.62124215

H, -3.15543517, 6.90985735, -8.09411857

H, -5.58245922, 6.68866418, -7.55739355

C, 0.29695613, 4.75995521, -2.54072816

C, -0.62638288, 5.78280232, -2.40201066

C, 1.61950546, 5.09299480, -2.94774758

C, -0.29416166, 7.12856373, -2.68452136

H, -1.64315279, 5.56320704, -2.07077313

C, 1.97386718, 6.39129106, -3.22286546

H, 2.35568026, 4.29679289, -3.07847763

C, 1.03327514, 7.44638200, -3.10764877

C, -1.24665150, 8.17607519, -2.57051754

H, 2.99054335, 6.62188138, -3.55224380

C, 1.35766101, 8.79578000, -3.39992345

C, -0.90150121, 9.47513485, -2.86030966

H, -2.26346736, 7.92862268, -2.25586842

C, 0.41333469, 9.78882360, -3.27850731

H, 2.37383453, 9.03270887, -3.72478085

H, -1.64450436, 10.27117796, -2.77166011

H, 0.67504387, 10.82534019, -3.50658862

Supplementary Appendix B: Molecular Coordinates (Å)

^{3}PC*/R−X for PC1

charge = 0, multiplicity = 3

C, 2.68357125431424, -3.01384809840874, -0.02662753706230

C, 3.05782291581401, -1.75563995905385, -0.42872158320506

C, 2.15049219764131, -0.89517711908349, -1.09302075984551

C, 0.78722963298383, -1.33703102653332, -1.34160642704566

C, 0.44209643303819, -2.64509927659099, -0.91908209549569

C, 1.34581797498276, -3.46746571311319, -0.28555629631126

C, 0.09495291349491, 0.74552228137746, -2.45368411879028

C, 1.39670712270711, 1.37953531186588, -2.34541646689906

C, 1.58790858729017, 2.68538063436542, -2.85879801532758

C, 0.56299448344910, 3.37122260486772, -3.46137591771402

C, -0.72782712654371, 2.75744983623952, -3.56648628892950

C, -0.94030391416158, 1.49077562027187, -3.07270079225040

S, 2.72273980346414, 0.64244017400612, -1.56232088126760

N, -0.13618928746327, -0.51987589563345, -1.95929695918057

C, -1.47434428979933, -1.03043490189957, -2.07825038038785

C, -2.38316965757591, -0.80124645624959, -1.04793627249957

C, -1.83541504437593, -1.74615051914611, -3.21719034756374

C, -3.67963401735457, -1.30160304765607, -1.16274781863584

C, -3.13408827710670, -2.24377504885108, -3.32321815551044

C, -4.05422258754065, -2.02217019460372, -2.29782285035303

H, 3.40020177170568, -3.66510822694025, 0.47780910169021

H, 4.07571655004654, -1.39917022317990, -0.24393929183350

H, -0.57027603341237, -3.00756134101699, -1.09812247577261

H, 1.03827098508490, -4.46948650910100, 0.02046235308299

H, 2.57677628252128, 3.14403899945383, -2.76275737832311

H, 0.73084235858892, 4.37638383340220, -3.85373607816358

H, -1.55286122584002, 3.29523436885425, -4.03830840965880

H, -1.93191525975938, 1.04604356022255, -3.15960206306750

H, -2.07041021261340, -0.23559317127570, -0.16794687412964

H, -1.10051655819147, -1.90935316804562, -4.00855386961563

H, -4.39837575595031, -1.12695509565608, -0.35865063098666

H, -3.42681391660308, -2.80768550416194, -4.21219960524542

H, -5.07091901913852, -2.41368429398847, -2.38407340677507

C, -3.32160769937694, -1.06391015294224, 2.71003741249687

C, -2.50915800178660, 0.06651550513146, 2.76266697506448

C, -1.13731931051860, -0.03209196794742, 2.49666648225110

C, -0.59114642063276, -1.28061020942898, 2.17308305577388

C, -1.40468621257758, -2.41129045168004, 2.13180769411525

C, -2.76997301772554, -2.30711426815658, 2.39721522515116

H, -4.39083162179107, -0.97345498764278, 2.91751106878602

H, -2.94128070597807, 1.04014819257493, 3.00964363805805

H, 0.47131942824556, -1.36880472593935, 1.93997294243540

H, -0.96606583042779, -3.37999326861375, 1.88096265071444

H, -3.40604696828393, -3.19494856412826, 2.35787318322795

C, -0.30045209398920, 1.21531057343173, 2.53615746689618

H, -0.80494036172448, 1.98331816898919, 3.13281130306513

Br, -0.13973670770213, 1.99536014058518, 0.73458446918963

C, 1.06271887352897, 0.99953027811026, 3.16823762609596

O, 1.25827575535013, 1.24431309825698, 4.33245457955355

O, 1.95706833588136, 0.49585249876649, 2.33586613283603

C, 3.25487476524913, 0.17597061971870, 2.85859445305387

C, 3.27529170213719, -1.19494161577685, 3.48605870044191

H, 3.54627370909822, 0.95305355825110, 3.58010226682562

H, 3.92489332018821, 0.23230011718968, 1.99046253106111

H, 2.58978712786486, -1.25080743194812, 4.34468050743151

H, 2.99682267562312, -1.97029096578776, 2.75644744045543

H, 4.29158117565105, -1.41576357575110, 3.84692978809273

^{1}PC*/R−X for PC2

charge = 0, multiplicity = 1

C, -3.01740592364594, 1.05335183949919, 0.88968861262040

C, -1.77785725333103, 0.42222369124150, 0.58292549673453

C, -0.87956687608174, 1.07110863374438, -0.31560260529844

C, -1.23628223771038, 2.34363155472241, -0.87460773989223

C, -2.47763474777827, 2.93008727639139, -0.53330378466393

C, -3.35169654406483, 2.28201443044501, 0.33913121739561

H, -3.71957622730170, 0.57528307657879, 1.57225305117185

C, 0.38038224570482, 0.48846386747463, -0.64658063006699

C, -0.33479174585600, 2.99714447545067, -1.74688219305065

H, -2.73800972309807, 3.90081907828531, -0.96348941925553

H, -4.30802061675174, 2.74553043223954, 0.59431791037124

C, 0.89030732780903, 2.41355481784519, -2.06983063656324

C, 1.24664495479651, 1.18498847339262, -1.53619391165737

H, -0.61514804465502, 3.96863444217599, -2.16258052383676

H, 1.57915138380882, 2.92993907302476, -2.74298245606174

H, 2.21620131184729, 0.76283417441040, -1.79407450755784

C, -1.39840019992147, -0.83075517510460, 1.17942603827254

C, -0.13034237102185, -1.40299689761984, 0.86406463802381

C, -2.24500610896132, -1.50624931942026, 2.10444157558772

C, 0.75840946629320, -0.76524480873485, -0.05200072628190

C, 0.27027565937404, -2.62628515642436, 1.49897808039527

C, -1.84925851511025, -2.69246219141829, 2.70506411265857

H, -3.22041945974005, -1.09047437535946, 2.35476705462378

C, 2.01586585275341, -1.37384222193166, -0.32597271517259

C, 1.53882638694028, -3.17873300535239, 1.20588325444242

C, -0.60603991935542, -3.25396908303239, 2.41571187160940

H, -2.51817676423656, -3.18800250242146, 3.41324236319139

C, 2.39369734426671, -2.55231282947637, 0.29814212346340

H, 2.71043156634532, -0.90377229263532, -1.01997046584706

H, 1.83636617634036, -4.10672769046363, 1.70145490880075

H, -0.29025130948279, -4.18493310177295, 2.89393626446930

H, 3.37057854338408, -2.99025383316805, 0.07823499888195

C, 4.42240530218232, 1.04721446313272, 1.70582706204991

Br 4.93098980276769, 1.11225750415020, -0.17664773936620

C 5.33985565546669, 1.95695927660508, 2.51298519877480

O 5.86342074499541, 2.96240455259945, 2.11342743158250

O 5.41144723285877, 1.49593483541894, 3.75514710496522

C 6.06967022757024, 2.32045253607759, 4.73120801990403

C 5.12849709717677, 3.36780423922899, 5.27591283536860

H 6.95696897312639, 2.77622266869578, 4.26879369421990

H 6.39376778681579, 1.62153328603963, 5.51338958827768

H 4.81947900720854, 4.06877355687367, 4.48635429831424

H 4.23027676258387, 2.90071890529325, 5.70696716621628

H 5.63397961372516, 3.94232580697882, 6.06653725147173

C 2.99568572417430, 1.46127336629264, 1.94993412151487

C 2.52028686153673, 2.70639275557723, 1.51859170809077

C 2.16011519121587, 0.62521392687636, 2.69622479437897

C 1.21951432527157, 3.10086951980761, 1.82079329416551

H 3.17256215227376, 3.36090034961134, 0.93477120255712

C 0.85963069315460, 1.02579352859494, 3.00449105815591

H 2.53009164318614, -0.34759723816270, 3.03073639503492

C 0.38633520967280, 2.26094120605054, 2.56470530663509

H 0.84996951827602, 4.06725898930323, 1.46904702873826

H 0.20909388457695, 0.36001007548694, 3.57661460112675

H -0.63844557485688, 2.56645499459286, 2.79038633926231

H 4.57083853348081, 0.00868504228879, 2.02469898105283

^{3}PC*/R−X for PC3

charge = 0, multiplicity = 3

C, -0.46803507337705, -3.59217977846356, 0.88347930119004

C, -1.29991732816428, -4.24470702080547, -1.35367680017514

C, -0.97665872518221, -2.72048273126062, 1.85733699799334

C, 0.31639183320698, -4.68192863615043, 1.28098881653883

C, -1.41590433622402, -3.94661164740954, -2.72412176498861

C, -1.81533588528161, -5.46859801890506, -0.89104803330024

C, -0.68406993606819, -2.92442030867243, 3.20346688731802

H, -1.59649106820182, -1.87443042352153, 1.55227554470404

C, 0.60001672953192, -4.88207869569511, 2.63041941264447

C, -2.00169671125538, -4.85450937779686, -3.59996183395299

H, -1.04976106490773, -2.99070397120921, -3.10059655737099

C, -2.39170156208047, -6.37100963007001, -1.78070863987409

C, 0.11001597244099, -4.00266089042799, 3.59690909259542

H, -1.08517204763075, -2.23515470926828, 3.95116836149329

H, 1.22200125234850, -5.73086361775457, 2.92641098576008

C, -2.48724780375509, -6.07971729274182, -3.14208216094305

H, -2.07558719698777, -4.59527882326708, -4.65956811109936

H, -2.78103811926254, -7.31688902561982, -1.39434558159798

H, 0.34247447462542, -4.15969649352903, 4.65293701438671

H, -2.94187674491987, -6.79271658980316, -3.83366514635417

C, -0.51335297940421, -1.97889653638950, -0.88896894848221

C, 0.77294238322275, -1.42939752409561, -0.93468320919089

C, -1.64504008309816, -1.09725053077928, -1.07354685953501

C, 0.98603419212863, -0.03943135108458, -1.11227319476192

C, -1.45147217857483, 0.30359611130943, -1.14419166459367

C, -0.16392257640647, 0.84916922583181, -1.10751373203181

N, -0.70732175784639, -3.32051632449230, -0.48892570693496

C, 0.00526724030796, 2.24776103824730, -1.10428880284172

N, 0.15398771937734, 3.40538806557317, -1.09238077227542

C, -2.94963932503399, -1.62824728865707, -1.12626708442696

N, -4.02439521844270, -2.08246281652460, -1.15760838826940

N, 2.24525213766739, 0.39811423902009, -1.57370096630597

C, 2.62850821554476, -0.04058975434512, -2.86487062600491

C, 2.97332161746911, 1.34961447858636, -0.86222233448121

C, 1.67532016830694, -0.04183779924302, -3.89437149025560

C, 3.92388396978113, -0.51180734902316, -3.11926310732925

C, 2.69093090241381, 1.55330934586024, 0.50117820902182

C, 3.97217014101556, 2.13274816307075, -1.47304038136506

C, 2.01377681662358, -0.51966684920809, -5.15712111739281

H, 0.66977513850131, 0.33633495882896, -3.69763694608815

C, 4.25169596026785, -0.98651245345353, -4.38608091499841

C, 3.41266034960348, 2.48216283281325, 1.23945551936001

H, 1.89848960460036, 0.97601901826544, 0.97610226622376

C, 4.68697838096480, 3.05701576614443, -0.72137334992597

C, 3.30091133549009, -0.99709079468299, -5.40860018884504

H, 1.26324552282809, -0.51469760539892, -5.95147609866868

H, 5.26085728052761, -1.36258576525479, -4.57205190780631

C, 4.42203768068794, 3.23627764576929, 0.63938480682356

H, 3.18802109564892, 2.61167694672241, 2.30121220333914

H, 5.45878286765092, 3.65614684498297, -1.21122318147130

H, 3.56366097676911, -1.37564918126599, -6.39921726286174

H, 4.98965925573609, 3.96501897680672, 1.22240120072964

N, 1.91039260405790, -2.23887318293015, -0.76349590058701

C, 2.82157492668157, -1.91743126151242, 0.26315861942258

C, 2.10717330523645, -3.37859459356439, -1.54999151652066

C, 2.32986040844579, -1.49739890312896, 1.50748611732286

C, 4.20536829166161, -2.04318755255382, 0.06012466710974

C, 1.68731132352691, -3.37605896754525, -2.89088842555674

C, 2.75669329851647, -4.51124538924096, -1.02213305377758

C, 3.21633955267862, -1.21335334132797, 2.54109746971098

H, 1.25230152664382, -1.43530647686762, 1.66303100491378

C, 5.07950221534380, -1.74102153715859, 1.09589192252075

C, 1.91561792569210, -4.48901338809170, -3.68976443616190

H, 1.21507409950014, -2.48651088496879, -3.30572515058383

C, 2.97245256798944, -5.61713799208021, -1.83073806818817

C, 4.59095755604245, -1.32889753449534, 2.33959170308969

H, 2.82803271655354, -0.90362677891547, 3.51385814131448

H, 6.15610371791210, -1.82461373274643, 0.93024636820169

C, 2.55534699581967, -5.61308681553013, -3.16635001472565

H, 1.59483276946217, -4.47277236137398, -4.73375366826077

H, 3.46386817699488, -6.49884278288087, -1.41304211919140

H, 5.28515137703237, -1.10083053813215, 3.15165236216673

H, 2.73128861439186, -6.48772819138401, -3.79678768643911

H, 4.66761173374206, -0.51129883068492, -2.32120825829296

H, 4.17980304008227, 2.02479699105526, -2.53816547408719

H, 3.06777921086298, -4.52389266177542, 0.02287181764121

H, 4.58674387978075, -2.36587730560209, -0.90939036502491

H, -1.77303689148481, -5.71589793931257, 0.17008456478941

H, 0.70946174928893, -5.36758215866455, 0.52921504282132

N, -2.56995788397383, 1.15246204838047, -1.25363372055124

C, -3.54109909148445, 1.13007786657007, -0.23371811109526

C, -2.66157049973326, 2.00040264492344, -2.37752116244619

C, -3.14788988651000, 0.84116843021916, 1.08100926904669

C, -4.89432513671633, 1.37405523079931, -0.50569356432387

C, -2.18921624265628, 1.55152114411608, -3.61906090446591

C, -3.18487662709612, 3.29585660545312, -2.26974301001884

C, -4.09183794065166, 0.78997300477729, 2.10205313606762

H, -2.09316212154475, 0.67208998214684, 1.30508707691152

C, -5.82680098111575, 1.34569675365085, 0.52776455534601

H, -5.21731311937964, 1.57946423634184, -1.52779101189748

C, -2.22605621997584, 2.39333033621595, -4.72778664270200

H, -1.79322423335428, 0.53744142107600, -3.71208698899877

C, -3.23660619108203, 4.12162881864130, -3.38877697026398

H, -3.53900236286577, 3.65646966916095, -1.30306560665464

C, -5.43647853256736, 1.04993924783314, 1.83543592116289

H, -3.76355418910899, 0.56616982102356, 3.12033282075841

H, -6.87809031311811, 1.54225543240528, 0.30168427151903

C, -2.75358131767413, 3.68062929602633, -4.62225518858268

H, -1.84879236085899, 2.03028761379462, -5.68734580237319

H, -3.64460176602723, 5.13077805461811, -3.28844305863004

H, -6.17534355641266, 1.02410291298910, 2.63978260374049

H, -2.78759917437110, 4.33710458957134, -5.49493128099900

C, -3.56076552058422, 4.53893160355368, 1.52629726956238

C, -2.23480062472891, 4.10820186353995, 1.47403232494857

C, -1.59844145590353, 3.65049066579756, 2.63242858663387

C, -2.29989031459410, 3.63420168415222, 3.84512266895438

C, -3.62449251180177, 4.05817995375268, 3.89181685156487

C, -4.25897995487877, 4.51003301590307, 2.73198506632355

H, -4.05199907039669, 4.89339113858240, 0.61665000169520

H, -1.68658587919294, 4.12206328610797, 0.52866367672655

H, -1.80747653201524, 3.27420456828934, 4.75128653161446

H, -4.16800436092671, 4.03357503330365, 4.83962987385774

H, -5.30089405794775, 4.83776875225353, 2.77013380431973

C, -0.16142615907410, 3.21388172422485, 2.55432709920940

H, 0.18641614658619, 3.23588651726837, 1.51521772746143

Br, 0.06233149048121, 1.35145289172746, 3.15115876512805

C, 0.73516031773304, 4.15707915095086, 3.33796244277943

O, 1.29689858256436, 5.07577458632123, 2.79666949937296

O, 0.76793515393733, 3.89830171530791, 4.63331452470744

C, 1.47928035142392, 4.81702265988896, 5.47893558117190

C, 0.60751944510465, 5.98210142527912, 5.87319295025498

H, 2.38744487147336, 5.14907869011828, 4.95564232741246

H, 1.77348834527879, 4.21999746267111, 6.35239326930942

H, 0.32103678680068, 6.58135654794980, 4.99616952738584

H, -0.30674771284845, 5.63910252854716, 6.38087880049552

H, 1.15967822614859, 6.63508443362576, 6.56620078161185

^{3}PC*/R−X for PC4

charge = 0, multiplicity = 3

C, 4.66421300280769, 1.49979631417875, 2.23659448342574

C, 4.81955063768354, 1.28932864267024, 0.86488827081870

C, 3.81415260912668, 1.70337835107665, -0.01484146139370

C, 3.48657155324312, 2.05097230468051, 2.72921734190611

C, 2.41936884256555, 2.36537572494061, 1.86635704941984

C, 2.62944237724569, 2.22999978813316, 0.47976534129145

C, 1.10510726787374, 2.70430877688264, 2.43009078422934

H, 3.34559128804638, 2.18111672204847, 3.80356156772851

H, 5.45855566193241, 1.18767291733321, 2.91937044875113

N, 5.93941830821706, 0.55360836671496, 0.37783581255833

H, 1.84417374764887, 2.51902915119089, -0.22200264843136

H, 3.95266586289283, 1.56500060315982, -1.08992675578789

C, -0.05481123872508, 2.40809040012950, 1.73428285817498

N, 1.05574570614681, 3.16024311996141, 3.71938896910355

C, -0.16286079948198, 3.28204837777294, 4.25541519976575

N, -1.31913412976082, 2.96966415405643, 3.69197824178952

C, -1.29679086362510, 2.50488531313953, 2.39887863984569

H, 0.00498877841374, 1.99104363601943, 0.72983738346786

C, -2.51239420807353, 1.98689255879220, 1.82473606827511

C, -0.21808394041565, 3.79020253175562, 5.65887690376883

C, -2.61411218600829, 1.62225109340184, 0.45206950624059

C, -3.73027005027408, 0.97773349251684, -0.04397055605704

C, -3.64827353407513, 1.73287598755159, 2.64488167835733

C, -4.76075236685845, 1.07157213240136, 2.15440659856707

C, -4.79949933455397, 0.67270555682099, 0.81158529434915

H, -1.79636612385646, 1.84367004790896, -0.23569325256593

H, -3.78148656668112, 0.68792061344498, -1.09639502472657

H, -3.60632599114185, 2.02318569498092, 3.69575450263790

H, -5.60224928481906, 0.83718381905731, 2.81145759282949

C, 0.95991898198409, 4.12008441690690, 6.34355360958460

C, 0.91246910660006, 4.58410984889364, 7.65679867626109

C, -0.31413045459473, 4.72721573568511, 8.30695421906592

C, -1.49342455563034, 4.40485400728750, 7.63201477508807

C, -1.44532732764160, 3.94121378029950, 6.31948920498263

H, -2.36139296759389, 3.68965686987350, 5.78224228267113

H, -2.45864479541831, 4.51674999126535, 8.13334373219623

H, 1.91274720849123, 4.00618498863437, 5.82369992743381

H, 1.84037861976954, 4.83612992093479, 8.17738853441180

H, -0.35180933252703, 5.09060008193692, 9.33738198162324

N, -5.89599381895769, -0.11251525392302, 0.32301203672805

C, -7.08393706857083, 0.50511120950215, -0.00139701042520

C, -5.71324056266465, -1.47433836658536, 0.18178572547907

C, -6.75461529576261, -2.30094030658410, -0.30504936527413

C, -8.19103978878405, -0.24832404672445, -0.46331861852286

C, -8.12895286759742, -1.75410706847617, -0.64608083343161

C, -7.19384083434323, 1.91031506633719, 0.11759901097485

C, -8.37278217828680, 2.55065349659511, -0.18503113133955

C, -9.50200722177024, 1.82579341302513, -0.62765121248376

C, -9.36483605338265, 0.43152664993150, -0.75734607785985

C, -4.46888833558988, -2.05083950518588, 0.53016962434597

C, -4.24764909406750, -3.39784505893636, 0.36448093799814

C, -5.25590631125017, -4.24318708626524, -0.14751022708249

C, -6.49711677292627, -3.65799081505140, -0.45457680361164

C, -9.16244377155144, -2.41435028125516, 0.28834063564145

H, -10.17657395789445, -2.05659568166816, 0.05811581611890

H, -9.15293125211110, -3.50686226568396, 0.16247784389610

H, -8.94345738137389, -2.18415139921616, 1.34167002805328

C, -8.45986299088183, -2.08928974516767, -2.11301366054007

H, -7.72913503156316, -1.63032282535985, -2.79551632402857

H, -8.45194585800275, -3.17658327496744, -2.27531585072486

H, -9.46128352084867, -1.72082212446337, -2.37767613699985

H, -6.32883351565060, 2.48918936431720, 0.43958277273139

H, -8.41273801121532, 3.63766269598361, -0.10236875210337

H, -10.22624570943706, -0.15017837636907, -1.08651455498560

H, -3.67739392648220, -1.42184732211078, 0.93467837626682

H, -3.28072002017539, -3.81008710750247, 0.65715223259414

H, -7.29045232978453, -4.29197424784977, -0.85239438096868

C, -5.01069885682061, -5.67521125699558, -0.34882810556253

C, -3.70562314422178, -6.16374775487596, -0.53174209709494

C, -3.44580972367698, -7.51444382708604, -0.72912174400911

C, -4.50679082440694, -8.43230111760281, -0.73961776294483

C, -6.06348067856077, -6.61406482280638, -0.37248227156393

C, -5.82007242920778, -7.96258764219837, -0.55899100037442

O, -4.36127457036266, -9.75054203332918, -0.91225163747628

C, -3.06420443691863, -10.27750526150736, -1.08175235243067

H, -2.86442973743920, -5.46846756285321, -0.54793687448743

H, -2.41672452336879, -7.84248821902306, -0.87899604905683

H, -7.09324900841336, -6.28609280394991, -0.21523691978470

H, -6.63890149424284, -8.68531549377181, -0.55984865272511

C, -10.76445325616504, 2.49914542786837, -0.95134878268327

C, -11.07310191612513, 3.76503109954757, -0.42276612181667

C, -12.26752581316915, 4.41296562296675, -0.71394726576271

C, -13.20142582030245, 3.80366085194922, -1.56550758220911

C, -12.90905806631310, 2.53968135378999, -2.10821569984151

C, -11.72012603609290, 1.90409563622814, -1.80324193498747

H, -13.63604875700830, 2.07713917770519, -2.77929437347732

H, -11.51891006109772, 0.93255697568946, -2.25911098673743

H, -10.37583918672195, 4.25648350375766, 0.25872037006754

H, -12.46691280501122, 5.38830145502146, -0.26905801530968

O, -14.37503845317538, 4.34376511546272, -1.90956322269934

C, -14.72026542543393, 5.61369235168766, -1.40242480220041

C, 5.78262455861007, -0.81782683496100, 0.18762168467567

C, 6.86701148423722, -1.63083366063597, -0.19564199747937

C, 8.27638376805649, -1.07317550439984, -0.38017153219127

C, 7.14565614915682, 1.18840008236206, 0.11208415238204

C, 8.28262986167694, 0.45096181247242, -0.27573003860989

C, 7.24173023321581, 2.58755663032068, 0.22319526071997

C, 8.43302074300353, 3.24722773460352, -0.03462387630674

C, 9.46680950714994, 1.14732026210626, -0.52373942697854

C, 9.58343098984287, 2.53996792591756, -0.41566702676246

H, 6.35962205799742, 3.16310553492209, 0.50702421611926

H, 8.45689057370051, 4.33680745453719, 0.04243468148564

H, 10.35531492018161, 0.57034042677525, -0.78865441336507

C, 8.81691017787313, -1.49451346236668, -1.75710146015819

H, 8.18605152643040, -1.09614745916708, -2.56648008506237

H, 8.84646903391624, -2.58982398293382, -1.85279511267296

H, 9.84267880882703, -1.12826755547276, -1.90987161811600

C, 9.18360870803083, -1.64758648323132, 0.72331439556013

H, 8.81782137969334, -1.36105838462070, 1.72132839079078

H, 10.21450049173363, -1.27608709588719, 0.61595770688827

H, 9.21354291775235, -2.74702072300145, 0.67216317658738

C, 4.52292403670678, -1.41467225276850, 0.38200741933084

C, 4.33281033598213, -2.77365335598740, 0.20118018545392

C, 5.39385480282667, -3.61020385503000, -0.17572392326784

C, 6.64337165535239, -3.00066199671585, -0.35986440688022

H, 7.49725142007871, -3.62821004847989, -0.62301600993586

H, 3.67238761619920, -0.79635171905342, 0.66481611614529

H, 3.32947889307659, -3.17806388637949, 0.34708970572977

C, 10.86216957477688, 3.22922683177752, -0.69072279366799

C, 11.78687148628663, 2.72162564447921, -1.61402600492894

C, 11.20240819392139, 4.42781629111244, -0.03509761388660

C, 12.39965346918662, 5.07955798109607, -0.28925893450556

C, 12.99643608464647, 3.36319683147314, -1.87906528475446

C, 13.31425669325752, 4.55450941062157, -1.21503944705528

O, 14.45116844122184, 5.24953165766178, -1.39891830052609

C, 15.40380628729009, 4.75738720906969, -2.30862373522087

H, 11.55550757275063, 1.80655764208613, -2.16504913608295

H, 13.67800761515225, 2.93062025134578, -2.61261154854546

H, 10.52204557979544, 4.85135113800216, 0.70787346171148

H, 12.65566549060293, 6.00434299048734, 0.23318588850197

C, 5.19137690568062, -5.06324664525617, -0.36432705806073

C, 4.12681475533533, -5.73678900025004, 0.26736909156601

C, 3.91884435339323, -7.09591889274641, 0.08792148814629

C, 6.03228515794546, -5.82928795226049, -1.18345638244485

C, 5.83852505426136, -7.19782259103785, -1.37137398367705

C, 4.77348447606439, -7.84634824975549, -0.73422032901364

O, 4.49818951076835, -9.15777902681255, -0.85262842855600

C, 5.32911899665860, -9.95100868502075, -1.66355087245978

H, 3.09131264083211, -7.60460722938526, 0.58869988125109

H, 3.44213915441807, -5.18956867017636, 0.91919058783615

H, 6.85777829608540, -5.34934064944980, -1.71471061542623

H, 6.51826925645352, -7.74520003829021, -2.02574089203719

H, 15.77467837615696, 3.76014837708371, -2.01526963315796

H, 16.24419781491186, 5.46374361638332, -2.29770181148518

H, 15.00146560350040, 4.70145922920387, -3.33483856167189

H, 6.37141434950137, -9.95783081014045, -1.30073260096878

H, 5.31888262039674, -9.61514152937572, -2.71483225300425

H, 4.93266353850752, -10.97370850702663, -1.61637203195040

H, -14.00235489183375, 6.38713839500412, -1.72238243141681

H, -14.78103986442017, 5.60859326733485, -0.30131602822567

H, -15.70930953827441, 5.85499625669202, -1.81213975404929

H, -2.57758992462162, -9.87620698903133, -1.98642269842999

H, -3.17985607220474, -11.36314682484456, -1.19177481546998

H, -2.42533329675906, -10.07257523384492, -0.20642758067821

C, -0.33734301027496, -2.26690440029623, 1.03599563734372

Br, 0.39702118418767, -1.30619839848135, -0.53329676403426

C, 0.28255599438442, -3.65346889210884, 0.95986428702859

O, 1.33659762139693, -3.96064538891307, 1.45891206599139

O, -0.48092295734749, -4.47044833680854, 0.25062111635380

C, 0.01226888771570, -5.80059009437003, 0.00090784740327

C, -0.22735815207141, -6.72412970181076, 1.16778145753511

H, 1.08096869841232, -5.73570519398481, -0.25160473551278

H, -0.53605350494742, -6.13000611899522, -0.89162853879523

H, 0.35972403878795, -6.42205719833430, 2.04682310864357

H, -1.29257311806299, -6.74826760963282, 1.44269960368602

H, 0.07414080239413, -7.74525156708155, 0.88833624901409

C, -0.08446727932386, -1.53387648959096, 2.31382881721539

C, 1.21207186551772, -1.25380420566566, 2.76651921776931

C, -1.17989134430708, -1.14009581355961, 3.09044750031825

C, 1.40413253896955, -0.60754473485870, 3.98352485255007

H, 2.07304386276785, -1.54533321301882, 2.16416084212132

C, -0.98491316163486, -0.48716598376909, 4.30800250761554

H, -2.19387756809114, -1.34941125745966, 2.73971925621943

C, 0.30698836462110, -0.22286356334925, 4.75796591265897

H, 2.41893736455037, -0.39525971417893, 4.32775223586745

H, -1.84854936654791, -0.17648148543642, 4.90073330154707

H, 0.46251186909968, 0.29075894532263, 5.70985099110851

H, -1.41116674774391, -2.33329795721778, 0.82855362409690

^{3}PC*/R−X for PC5

charge = 0, multiplicity = 3

C, 2.94528727653704, -2.90150046010611, -0.10466962723716

C, 3.20270927506464, -1.58218827966297, -0.50596612505032

C, 2.26408600514016, -0.78918551298321, -1.15074136027707

C, 0.95682284915112, -1.34456481323243, -1.43444116831963

C, 0.65935791434805, -2.62031813054651, -0.97699812558590

C, 1.56629932000086, -3.40378248802860, -0.24985118516669

C, 0.32337580526983, 0.72448932583257, -2.49943936148982

C, 1.60707040167583, 1.26724927344679, -2.22650755227416

C, 1.87251004185948, 2.59132173515759, -2.59952544092749

C, 0.92675468728502, 3.38678392925049, -3.23890779355421

C, -0.34147671129920, 2.82193115908304, -3.56549242566227

C, -0.61507459683784, 1.51512598956983, -3.17473200488338

N, 0.02792042657652, -0.55578153625963, -2.08247492164993

C, -1.30030020657999, -1.05790828407850, -2.25430684983662

C, -2.26644427100937, -0.75281086111654, -1.29549064702609

C, -1.60279587428959, -1.84911856130529, -3.35847585629755

C, -3.55491475454392, -1.24946167214540, -1.44653375021470

C, -2.89392684173941, -2.35128522566028, -3.50463904404649

C, -3.86310698465974, -2.05165077276806, -2.54869003993381

H, 4.20908632053562, -1.18303208415459, -0.38651507370801

H, -0.36204036429002, -2.97769542643100, -1.09908487798804

H, 2.83659429000595, 3.02649679822844, -2.33610136174854

H, -1.58225758200000, 1.07959546025760, -3.42626933792099

H, -2.00325312025601, -0.13364466317267, -0.43570167556729

H, -0.82955246282851, -2.07208005938041, -4.09601102453126

H, -4.31649682810198, -1.01597752244191, -0.69941016031415

H, -3.14121309183463, -2.97544338188637, -4.36467498104894

C, -3.43622889480664, -1.05580291976451, 2.31518608762989

C, -2.56146740512902, 0.01590225728409, 2.47652850919009

C, -1.18038040823557, -0.16467041940623, 2.32098961522196

C, -0.68891418747430, -1.43627112778420, 1.99978532152861

C, -1.56589007694445, -2.50904161368195, 1.84997110289592

C, -2.93919880704539, -2.32216312067404, 2.00308267782665

H, -4.51132251379848, -0.90138435081206, 2.43651432598302

H, -2.95062210166800, 1.00719014189776, 2.72484657392097

H, 0.38132028322624, -1.59209333202624, 1.85667731140209

H, -1.17083464143804, -3.49617199251078, 1.59958168111934

H, -3.62399425313519, -3.16403257776468, 1.87368715559133

C, -0.27626189336347, 1.02374140198353, 2.48554477651618

H, -0.77364978059587, 1.78723690011125, 3.09365253430983

Br, 0.03853063508093, 1.91092564583252, 0.74892082871880

C, 1.02967406978559, 0.69219832002850, 3.18502686357236

O, 1.15861239325919, 0.85287531310437, 4.37199368573565

O, 1.95252142574319, 0.18666340662595, 2.38196396238054

C, 3.17760382115104, -0.27408869376546, 2.97826910214348

C, 3.02028007826192, -1.65024420924449, 3.57211799412856

H, 3.50136828263176, 0.45998154681742, 3.73039269213233

H, 3.90048848061824, -0.27596361409909, 2.15223981638342

H, 2.29799372479899, -1.64984699112145, 4.40157403568501

H, 2.68679343207457, -2.37147142165013, 2.81187538545499

H, 3.99006657305498, -1.99206859063928, 3.96403063944961

N, 2.53570001328180, 0.50162882166784, -1.55268947740464

C, 3.79762185149960, 1.07864427782767, -1.20712561849285

C, 4.85688217684677, 1.01145958859316, -2.10785875200873

C, 3.94129254519761, 1.69292755825480, 0.03582659807937

C, 6.08264647789995, 1.57248088421935, -1.75976495048189

H, 4.71987003513792, 0.52014479860801, -3.07306671745726

C, 5.16651364064171, 2.25027043795320, 0.38243564723477

H, 3.09052193874956, 1.72397490169523, 0.71839356208466

C, 6.23343285043276, 2.18851025697308, -0.51749206672788

H, 6.92074357429400, 1.52462772703565, -2.45711273529236

H, 5.28964775667614, 2.72988337174899, 1.35572294197780

C, -5.25894594484122, -2.59251186440799, -2.67143552415985

C, 7.53761050457922, 2.83779686304382, -0.15067730262850

F, -5.43788514725244, -3.30010162778188, -3.78845859498165

F, -6.17017649470309, -1.61012469374028, -2.67400405427393

F, -5.56633459363945, -3.39425507887782, -1.64184663134837

F, 7.50216805977775, 4.16308141121772, -0.35443675668804

F, 8.55957888591176, 2.36222672570838, -0.86696526685284

F, 7.83735116231599, 2.65763925575527, 1.14076853573285

C, 4.05585239528743, -3.73117443158800, 0.33142758309625

C, 4.07065502627803, -5.12146968140596, 0.14469380151644

C, 5.19249458079058, -3.14697135127242, 0.97933214192263

C, 5.14357455922311, -5.92829176648694, 0.58818869697961

H, 3.24990366761345, -5.60987880288467, -0.38150965621525

C, 6.24175537618201, -3.90554367285275, 1.42632398337045

H, 5.20791079118226, -2.07004520624875, 1.16260514178873

C, 6.25797697670065, -5.32159255086242, 1.25268714014730

C, 5.15151967005561, -7.33836379138261, 0.40258790411240

H, 7.08166547683958, -3.42886637086299, 1.93952452582999

C, 7.32044724194299, -6.13428330854710, 1.70699370139154

C, 6.20489751660789, -8.10576804121719, 0.85415284445165

H, 4.30186116738416, -7.80567984774698, -0.10343068874927

C, 7.29914245668108, -7.50366301579179, 1.51334680279998

H, 8.16515747377998, -5.66023570214726, 2.21541682446574

H, 6.19357681653508, -9.18872456467816, 0.70376408448361

H, 8.12758586363555, -8.12175141668722, 1.86865735955355

C, 1.05785519176256, -4.57302652294354, 0.44891864800253

C, 1.58912428563418, -5.00195098363612, 1.67377653753477

C, -0.05290339566948, -5.31103830400768, -0.07481758886844

C, 1.06141213249441, -6.10983286275369, 2.37612551041844

H, 2.41162474806284, -4.45495439472706, 2.13389237727456

C, -0.57877556852132, -6.39326940612950, 0.58005399733407

H, -0.47665856009510, -5.02462864388750, -1.04019516507126

C, -0.04574423762786, -6.83381858476518, 1.82784849783162

C, 1.59923523824917, -6.53469219482275, 3.62204184558420

H, -1.41525290129184, -6.94462231257332, 0.14150202628991

C, -0.56445377040803, -7.94387965564396, 2.53127030680495

C, 1.06941670885315, -7.62022675467301, 4.28780055277969

H, 2.44518326768970, -5.98387327247449, 4.04328243520309

C, -0.01977770120632, -8.33397869402740, 3.74118960701537

H, -1.40896683947772, -8.49105771099777, 2.10225434934854

H, 1.49542390016138, -7.93319623430918, 5.24490116445843

H, -0.43058052194161, -9.19357961935357, 4.27683140426568

C, -1.37375290918107, 3.55594757065778, -4.33212475719987

C, -1.05474440982569, 4.25620594513861, -5.48231664458728

C, -2.72793523676485, 3.53802934540026, -3.89448053866753

C, -2.03753255969734, 4.96759525052316, -6.21138424857236

H, -0.02561468921578, 4.27072455255426, -5.84873179666589

C, -3.70472152535916, 4.21794616554660, -4.58168533987809

H, -2.98467303881729, 2.99107533924598, -2.98361870216359

C, -3.39260868384056, 4.95570200577033, -5.75320570007295

C, -1.71443700108991, 5.70189567971605, -7.38430467755916

H, -4.73831388553424, 4.20529222421103, -4.22534036289580

C, -4.37258438920788, 5.67917479754997, -6.48120825672554

C, -2.68718095674559, 6.39407562148112, -8.06821474636996

H, -0.67708234622437, 5.70984744342313, -7.73001637361629

C, -4.02799256917466, 6.38283265805318, -7.61328125858352

H, -5.40677956118386, 5.66783271384670, -6.12612455926884

H, -2.42736476831524, 6.95806706323269, -8.96778086120713

H, -4.79030338344826, 6.93723640591410, -8.16663876566627

C, 1.26686870074506, 4.80347203913328, -3.50349944647331

C, 0.39335929783542, 5.82450673572655, -3.17231936532431

C, 2.51951584663897, 5.13695186062104, -4.09051788310378

C, 0.71053329837075, 7.18039587308430, -3.42637433790389

H, -0.56643456750745, 5.59502313920015, -2.70348809063450

C, 2.85607224127236, 6.44412702871897, -4.34947849275608

H, 3.21164649097065, 4.33433081857462, -4.35736323086068

C, 1.96543111667663, 7.50267849719661, -4.03288882999331

C, -0.18702063895640, 8.23326185149332, -3.10180144762114

H, 3.81680083659644, 6.68334913346052, -4.81345964010045

C, 2.27476063929246, 8.86255097313390, -4.29576854408776

C, 0.14181587568535, 9.54229876282428, -3.36915829184178

H, -1.14557557868476, 7.98251690322830, -2.63913589324538

C, 1.38328666121412, 9.86019047932026, -3.97138943552318

H, 3.23466424156579, 9.10348224664597, -4.76093513151204

H, -0.55677946916616, 10.34439910210373, -3.11748304138321

H, 1.63120248791016, 10.90450602743468, -4.17796703814288

$PC^{\cdot+}/X^{-}$ for PC1

charge = 0, multiplicity = 2

C, 2.57576674848402, -3.03163769511541, 0.21554933964432

C, 3.02396124652432, -1.82896575829558, -0.30852121328665

C, 2.15638138131815, -0.99456715759237, -1.02390778082901

C, 0.80652091650424, -1.36015557362447, -1.22350812774973

C, 0.37505690147209, -2.58762979765340, -0.68370470080317

C, 1.24345482787717, -3.40669905070384, 0.02050247741512

C, 0.16438004571227, 0.74209669362568, -2.30832355125130

C, 1.45519115959998, 1.33282844691496, -2.19883291301100

C, 1.67448001463362, 2.65227253110394, -2.62419950802786

C, 0.64591758368754, 3.39536279297335, -3.17026501974506

C, -0.62820559660334, 2.82123998871072, -3.29688303476697

C, -0.86737760580322, 1.53070353128161, -2.87227460172415

S, 2.85662150067512, 0.44951142438887, -1.69887585944903

N, -0.08674578432369, -0.55930739011267, -1.93316685085388

C, -1.43932784920777, -1.03243325986908, -2.05023512875326

C, -2.35069969417657, -0.72073630377643, -1.04115467276026

C, -1.81008403149053, -1.79014425381609, -3.15745844162895

C, -3.66001154945571, -1.18408499703952, -1.14837966481772

C, -3.12340605058187, -2.24932136747642, -3.25422091781948

C, -4.04561646034796, -1.94817549426405, -2.25177196203718

H, 3.26051456767201, -3.67358444017076, 0.77347393152502

H, 4.06267857044199, -1.51872991351615, -0.16619036546784

H, -0.65970542606657, -2.89863361275588, -0.82074905006000

H, 0.87227299297986, -4.35068069414651, 0.42556230649307

H, 2.67543468106260, 3.07993186969033, -2.52504921150819

H, 0.82787763090234, 4.41987013964818, -3.50140121698673

H, -1.44768199017125, 3.39852723671651, -3.73073346307621

H, -1.86477882492304, 1.10670784376604, -2.97635458375889

H, -2.01505427818302, -0.10584210304090, -0.19960828979921

H, -1.07245699702155, -2.01505101409283, -3.93093975799893

H, -4.38479328480623, -0.94586342481657, -0.36594692128188

H, -3.42625734714574, -2.84496759876152, -4.11875835606245

H, -5.07374873236119, -2.31011886770718, -2.33163460897883

Br, 0.01173444720684, 1.79332154990153, 0.60530125642990

$PC^{\cdot+}/X^{-}$ for PC2

charge = 0, multiplicity = 2

C, -3.07283491462460, 1.03441565032621, 0.85182576830656

C, -1.82624882518011, 0.43276386582861, 0.59165576460018

C, -0.92040121431027, 1.09112089606579, -0.28930192423457

C, -1.29863385142719, 2.33368210510915, -0.88444921323644

C, -2.56017039125556, 2.89350429638833, -0.59257037957422

C, -3.43381175421591, 2.24553194176639, 0.26868555724573

H, -3.78350509851563, 0.55395667322253, 1.52423097624737

C, 0.35728791562110, 0.53450419623327, -0.59096091506786

C, -0.40417586562168, 2.99037458176836, -1.76232299898194

H, -2.83627247554571, 3.84401202984621, -1.05561060442068

H, -4.40905829190759, 2.68198475401316, 0.49355296147057

C, 0.83443474435719, 2.43759601723899, -2.04448217368710

C, 1.21849061332081, 1.22883796056595, -1.46673360186830

H, -0.70925845349014, 3.94012275429229, -2.20876710547192

H, 1.52479430706194, 2.94598022594263, -2.72145708410884

H, 2.21360336238498, 0.83236358105191, -1.72219470180882

C, -1.44049450715261, -0.82953013533059, 1.19130469068219

C, -0.16407520330935, -1.38510889549987, 0.88782355383558

C, -2.29025510860330, -1.53026519309059, 2.06879942840095

C, 0.74133389362182, -0.72506907653073, 0.00569402237465

C, 0.21744049051662, -2.62898143314519, 1.47814053391299

C, -1.91147087073903, -2.74171986211414, 2.64051419409998

H, -3.27226941563100, -1.12815567816295, 2.31673852932153

C, 1.99231070609799, -1.31931328505673, -0.26347725110698

C, 1.48307803530796, -3.18507008198469, 1.18050779821422

C, -0.67091048285017, -3.29099208120787, 2.35251714605938

H, -2.59730319156061, -3.25580782030297, 3.31688491171463

C, 2.35180273902746, -2.53241870160138, 0.31974188303079

H, 2.71632335795811, -0.84320567043914, -0.94237995074584

H, 1.76059666078720, -4.13660125848897, 1.64095642309674

H, -0.36438029995655, -4.24102824659639, 2.79688344429289

H, 3.32807486779768, -2.96559768379436, 0.09052525141969

Br, 4.42148991203614, 0.09809136368677, -2.55061228401312

$PC^{\cdot +}/X^{-}$ for PC3

charge = 0, multiplicity = 2

C, -0.09071795742531, -3.82303446765199, 0.87526745460057

C, -1.75918205461065, -3.93226921840797, -0.93538582791300

C, 0.16668415431386, -3.05398581639549, 2.01448165986099

C, 0.29774519949176, -5.17004227217474, 0.83914371459877

C, -2.01074787201882, -3.63607689444589, -2.28242947909855

C, -2.53348663431061, -4.90895595841610, -0.29410175821754

C, 0.83541606119045, -3.62260658381156, 3.09653615637979

H, -0.14806468018363, -2.00357381504203, 2.07098983614985

C, 0.93993919144491, -5.73262574820044, 1.93739684489595

C, -3.01209263613685, -4.31298846424145, -2.97318715917316

H, -1.43641780321556, -2.86199468653172, -2.79212389701121

C, -3.51989546853145, -5.59072226181207, -0.99865480122386

C, 1.22199689944700, -4.96147906832597, 3.06747590886023

H, 1.04102866144227, -3.00188856904134, 3.97254138652643

H, 1.23905833725260, -6.78316902850447, 1.90051155140469

C, -3.76650481389441, -5.30059687397294, -2.34171744989434

H, -3.19485676922705, -4.06604892206620, -4.02197611025174

H, -4.11552968408864, -6.34793498482593, -0.48253606982973

H, 1.73680535371190, -5.40696748506685, 3.92222197622098

H, -4.54672976288118, -5.83515544910357, -2.88845293766071

C, -0.50789930785148, -1.88630063722444, -0.52987629699384

C, 0.80582473560746, -1.42211495345752, -0.81687209684181

C, -1.58092558821995, -0.97277888045349, -0.55289772516391

C, 1.01163213787046, -0.06372477104679, -1.17069242952110

C, -1.40275270867654, 0.37341734728088, -0.95346781475357

C, -0.08913710528702, 0.80750930290188, -1.24950656163125

N, -0.75102721942460, -3.23011296025167, -0.23004008847639

C, 0.14149124691785, 2.15334513795694, -1.65960524567741

N, 0.34948649618215, 3.24550006138886, -1.98904379964210

C, -2.87512269069626, -1.44317102256717, -0.18316035003607

N, -3.91725158970393, -1.84317460388121, 0.13109095710269

N, 2.29060702547193, 0.38570668437300 -1.52874967795448

C, 2.88138260304953, -0.18509137905291, -2.68605285943798

C, 2.85394437763941, 1.51286092923234, -0.89170470320875

C, 2.08696947868324, -0.44032573288946, -3.81024714026466

C, 4.24987271328058, -0.48291129765618, -2.72214335905137

C, 2.47356892127162, 1.83168486533858, 0.42007493897158

C, 3.77218751590565, 2.33688929767300, -1.55978220928734

C, 2.64910094204741, -1.02071106210425, -4.94541289132267

H, 1.02692097873135, -0.17695593868371, -3.79569203995242

C, 4.80424483315594, -1.05023369238554, -3.86526481440698

C, 3.02190456905437, 2.94387939423653, 1.05160937376253

H, 1.72291865651381, 1.24221747710809, 0.96048024504254

C, 4.32396843312871, 3.43579978104066, -0.90836330112431

C, 4.00737361669033, -1.33135862522228, -4.97701262326105

H, 2.01705632211111, -1.22214344567109, -5.81372163945133

H, 5.87183060122637, -1.28329172804913, -3.88227925192292

C, 3.95900651424761, 3.74710761351625, 0.40274098661211

H, 2.70553648128376, 3.17266245734463, 2.07279259301979

H, 5.03984487360106, 4.06542510536616, -1.44314367927582

H, 4.44799932064504, -1.78384185942720, -5.86852617852118

H, 4.39374832086216, 4.61332134629708, 0.90714356882962

N, 1.90254839653599, -2.29986862456906, -0.76989712295216

C, 2.99329692832015, -1.98718340162015, 0.05594866242540

C, 1.89545274003439, -3.46628879386542, -1.55017886899080

C, 2.77386141120929, -1.25866774259808, 1.23808013635917

C, 4.29693811283763, -2.40147209623131, -0.28338495430008

C, 1.23391314679751, -3.46567025010712, -2.78975400869297

C, 2.54862810088221, -4.63190915514153, -1.10660915097718

C, 3.84415312772204, -0.96298924465250, 2.07074309311782

H, 1.77000867465415, -0.92728845920537, 1.52987875308791

C, 5.35222496518481, -2.10441721766366, 0.56546659649969

C, 1.23914310425774, -4.60710235075532, -3.57796122594745

H, 0.74512368065668, -2.55755231854886, -3.13966803618446

C, 2.55787032283727, -5.76174850244367, -1.91196827500342

C, 5.13361883884966, -1.38720601116141, 1.74664616808039

H, 3.65506931435946, -0.39946443750399, 2.98739075500387

H, 6.36207217267601, -2.41953628689756, 0.29350048710621

C, 1.90558977466456, -5.75703255303883, -3.14736087039880

H, 0.72962892461626, -4.59357372817749, -4.54391215799977

H, 3.06117301136135, -6.66488337149962, -1.55984661960468

H, 5.97157839012699, -1.15609627249720, 2.40850330631350

H, 1.91330006797768, -6.65193027119434, -3.77382398591586

H, 4.87666998059597, -0.26349911332658, -1.85771652632427

H, 4.04771300883839, 2.12904999113541, -2.59438909031788

H, 3.02065568670917, -4.65562816157026, -0.12501331712532

H, 4.48153681339936, -2.92604786770237, -1.22048030417205

H, -2.37514993795286, -5.12347546182934, 0.76363387630915

H, 0.09739767514558, -5.77345044834751, -0.04685956048054

N, -2.47772684423180, 1.23566262781646, -1.10592346265891

C, -3.48168738047541, 1.32563126612283, -0.11086548134043

C, -2.60180162501177, 1.97810915216600, -2.30712302686501

C, -3.11261631155990, 1.29838756443235, 1.23808559157085

C, -4.83116937564168, 1.42412368306169, -0.47434830391132

C, -2.29991794949571, 1.36695674805409, -3.52991177361842

C, -3.01497779808741, 3.31510610725195, -2.27331613088665

C, -4.10072397124190, 1.34267753348716, 2.21812137934202

H, -2.05720020093219, 1.21653831917338, 1.53292242583942

C, -5.80471270381054, 1.48627502107424, 0.51616717543305

H, -5.11449738286034, 1.43028860318301, -1.52862427790239

C, -2.38150132242733, 2.10281627493737, -4.70922307824542

H, -2.00311687660958, 0.31556673274821, -3.55266284818484

C, -3.10980425729572, 4.03488964583228, -3.45889094465243

H, -3.23936135524931, 3.78974935583858, -1.31609399204564

C, -5.44664143370000, 1.43965890889368, 1.86639385321556

H, -3.80371384627913, 1.30765634499839, 3.26961449792643

H, -6.85722258376399, 1.55598489909607, 0.23019103859349

C, -2.78653241740805, 3.43694182486278, -4.67960522036013

H, -2.13955502752045, 1.62187933308288, -5.66015630689035

H, -3.42438956842168, 5.08094763519533, -3.42773026683970

H, -6.21782873233968, 1.47979993252901, 2.63960289860411

H, -2.85434279940110, 4.01121010100327, -5.60656730302622

Br, 0.04165010337719, 0.48737189318035, 2.78101691260326

$PC^{\cdot+}/X^{-}$ for PC4

charge = 0, multiplicity = 2

C, 4.81769868489986, 0.75813911107528, 1.63910095253048

C, 4.92949305470361, 0.46791339654468, 0.27895879832219

C, 3.80754544957649, 0.43588255754511, -0.54297756833453

C, 3.56332690141591, 1.01429489432019, 2.17649086054832

C, 2.41543928949451, 0.97488894084989, 1.36992461251298

C, 2.55336174669696, 0.68872247080357, 0.00461279618579

C, 1.08931162535751, 1.23983798190210, 1.97766656374054

H, 3.45576252559317, 1.23958226846724, 3.23852598550321

H, 5.72446239515183, 0.78127067052436, 2.25138337546305

N, 6.22597272936525, 0.19353339625803, -0.26646455615709

H, 1.68122444018061, 0.67591097355058, -0.65137382985334

H, 3.92067571973899, 0.21721303352726, -1.60670502073619

C, -0.10392354119831, 0.87148535655024, 1.34955685435560

N, 1.07476689403959, 1.83538632696059, 3.17289784107313

C, -0.10547132577920, 2.06978804718368, 3.74521349275142

N, -1.28577643921685, 1.76147282652616, 3.20881770886783

C, -1.29881767686977, 1.16656646141955, 2.01313897258557

H, -0.10323150921630, 0.34383810017098, 0.39728849882846

C, -2.62188282093755, 0.82186518211308, 1.44185215685567

C, -0.10587040133013, 2.72851791122020, 5.07558750077615

C, -2.78814086621117, 0.57463583937937, 0.07149921430346

C, -4.03994995598970, 0.25248632015099, -0.44165687707359

C, -3.73987341026705, 0.74370173914091, 2.28491980325372

C, -4.99032031412410, 0.41577386573631, 1.77534073912889

C, -5.14243557561748, 0.16940566608326, 0.40971350762305

H, -1.94067975348661, 0.65123101805247, -0.61245745297347

H, -4.17122287869174, 0.06767851308966, -1.51002124784012

H, -3.61359862096520, 0.93352418956433, 3.35187914607951

H, -5.85892306176236, 0.34547755215677, 2.43380367032982

C, 1.10189822330511, 3.08089924575398, 5.69443144221369

C, 1.10023590455037, 3.69823160288010, 6.94225267831956

C, -0.10668661366933, 3.97223059443433, 7.58779638294598

C, -1.31321788642750, 3.62495745940358, 6.97773866901678

C, -1.31410657708322, 3.00770911103094, 5.72985922910275

H, -2.25369712143603, 2.73565697578819, 5.24598414747034

H, -2.26130621827272, 3.83819579045932, 7.47803876071185

H, 2.04178087881352, 2.86504075597830, 5.18357445446221

H, 2.04793889663894, 3.96855010670699, 7.41491756362112

H, -0.10699262289134, 4.45683257122209, 8.56762692666963

N, -6.42400974784702, -0.16572831745482, -0.11466417220778

C, -7.27162459093576, 0.85420439381521, -0.53879383045239

C, -6.80275372239568, -1.50419628699231, -0.17538378645078

C, -8.06542469615515, -1.87710204476771, -0.67585052301396

C, -8.54847762636038, 0.56465984869129, -1.05807961632066

C, -9.08206856404745, -0.85941053480643, -1.18573736225622

C, -6.85716762526760, 2.19664523143544, -0.45464533999577

C, -7.68188951633836, 3.22985523644511, -0.87107544327169

C, -8.95915385568497, 2.97201408504427, -1.39026451140456

C, -9.35340091904038, 1.62972536679511, -1.46697488682258

C, -5.92139838382402, -2.50748374236014, 0.26921278158582

C, -6.27585438753014, -3.84653025206125, 0.22262775701819

C, -7.52575218543953, -4.24793271215551, -0.27146908294030

C, -8.38876444575801, -3.23495951082845, -0.71015692627638

C, -10.37660207384745, -0.98898625752165, -0.36335676665556

H, -11.14217304119923, -0.28264032632660, -0.71798552725957

H, -10.79769170412833, -2.00200493527571, -0.44859740991575

H, -10.18773746199663, -0.78448324750247, 0.70166766819282

C, -9.38415837060666, -1.14829727438951, -2.66706894542229

H, -8.47241868727259, -1.06227143521418, -3.27790297588980

H, -9.78957247704956, -2.16307451178024, -2.79683615875049

H, -10.12895375817762, -0.44077118613301, -3.06176148961207

H, -5.86659401287810, 2.43295734558472, -0.06514843329998

H, -7.31077076604022, 4.25540040001540, -0.80710702222638

H, -10.35066292112006, 1.40132190219175, -1.84885344266908

H, -4.94555284618478, -2.23117515617289, 0.66941800503660

H, -5.56841627082865, -4.58900332003082, 0.59921615123358

H, -9.36016882263099, -3.51983307822639, -1.11962639097338

C, -7.91812248848911, -5.67241552892988, -0.32661282405858

C, -6.96503201316678, -6.68682784378741, -0.49181086617090

C, -7.31995589473110, -8.03427972030353, -0.54390924605848

C, -8.66541665320652, -8.40591725321759, -0.43141943426724

C, -9.26428376924510, -6.06878188603002, -0.21365867270051

C, -9.63411146119399, -7.40422791895970, -0.26511240469243

O, -9.11047182636942, -9.67454713564688, -0.47115865017041

C, -8.17502369746083, -10.71289447888162, -0.62867954598687

H, -5.91007873131122, -6.42499611833422, -0.60475414151279

H, -6.54046646304673, -8.78460928711330, -0.68178556772022

H, -10.04113042307847, -5.31556996618319, -0.06087192332090

H, -10.68144621598947, -7.69943334818408, -0.16721843723100

C, -9.85081287447275, 4.06408958981541, -1.83559260235661

C, -9.80176961675665, 5.33345467383276, -1.24309569735988

C, -10.63543019388746, 6.37375156457148, -1.65210186951492

C, -11.55882786670698, 6.16277584127720, -2.68358619378670

C, -11.62254591423543, 4.89830511857689, -3.28897753811975

C, -10.78501035071021, 3.87479711110416, -2.87173462140966

H, -12.33729323361347, 4.74186035452720, -4.10034199744747

H, -10.84482402609413, 2.90891539095359, -3.37957069403614

H, -9.10689607239238, 5.51991288297225, -0.42044544432021

H, -10.56270917025626, 7.34087386784753, -1.15308910114867

O, -12.40729002143639, 7.09709012609733, -3.14849581162191

C, -12.37094924178271, 8.38343233274916, -2.58111368447427

C, 6.71736366551687, -1.10088586215336, -0.16344002383418

C, 8.02562190768686, -1.40486050490090, -0.60088716775108

C, 8.85304343347944, -0.37591852899145, -1.34988527677508

C, 7.04212658017224, 1.25084818192390, -0.57922382456518

C, 8.37012376612308, 1.02693139939106, -1.03661638232438

C, 6.56453968275525, 2.57770017455987, -0.45684647490464

C, 7.37250183121443, 3.64599403958069, -0.76549920847860

C, 9.15077180056562, 2.13596885669606, -1.35265456903253

C, 8.69663430497524, 3.45322621516870, -1.22315403160586

H, 5.54338313739448, 2.75714446140189, -0.12257041940125

H, 6.96370101335759, 4.65417242945577, -0.67578363408284

H, 10.17032703078314, 1.96711932192540, -1.69612415020066

C, 8.60223835896869, -0.60884073125465, -2.86014665546913

H, 7.53508916996960, -0.50164551864321, -3.10840662850085

H, 8.92194430568297, -1.62360075880800, -3.14304967888344

H, 9.17103543899197, 0.11991120853668, -3.45778222071081

C, 10.34628444818211, -0.53047545976686, -1.05387831258323

H, 10.53661819279756, -0.35471819040224, 0.01557202107929

H, 10.94230283788818, 0.17765150152344, -1.64524525285815

H, 10.70334197321315, -1.53198678517971, -1.32843118035881

C, 5.91668709051990, -2.11470757673456, 0.39802064536233

C, 6.40297398779158, -3.39912911393032, 0.54276158886766

C, 7.70673495716266, -3.73028486215115, 0.12933643884357

C, 8.48219413046248, -2.71037251648844, -0.44168657076178

H, 9.49579200353467, -2.94745654583570, -0.76331035816460

H, 4.90080558651137, -1.89017285086650, 0.72171696466331

H, 5.74823999630159, -4.16283937564853, 0.96682064853111

C, 9.55895267351777, 4.59706502673322, -1.55867261185833

C, 10.60626195553238, 4.47921645910856, -2.48684037221768

C, 9.36835341110889, 5.85857448347436 -0.95847448246452

C, 10.18195474195922, 6.93557821914711, -1.26572662469648

C, 11.42857610151124, 5.55379861598058, -2.81082144410201

C, 11.22364611246451, 6.79827586181770, -2.19808165315444

O, 11.96016716694237, 7.89170084399314, -2.43971550287100

C, 13.02214989980697, 7.80710225714395, -3.36130113066819

H, 10.77717243136263, 3.52788087895601, -2.99577793393400

H, 12.22083547595383, 5.41490042222197, -3.54718103668628

H, 8.58157044325406, 5.99499846886736, -0.21342248763923

H, 10.03339340930722, 7.90515772530846, -0.78514576590270

C, 8.23410245740875, -5.09877184189607, 0.28091834034068

C, 7.76595343869536, -5.95372660421248, 1.29828925863153

C, 8.25998344575483, -7.23911062487976, 1.44713125796931

C, 9.21981618313572, -5.60475018450835, -0.57987741559698

C, 9.72418281654194, -6.89584388658640, -0.44563478028888

C, 9.24687472543901, -7.72820234431318, 0.57591587560513

O, 9.66855775076060, -8.98373959980672, 0.79077055453935

C, 10.66594102563149, -9.51795198086375, -0.04708668097062

H, 7.89778096536276, -7.88931032628399, 2.24674399385891

H, 7.01395215873042, -5.59737173305841, 2.00602050525898

H, 9.59571618760626, -4.98665396742501, -1.39860499140425

H, 10.48194587830428, -7.24647202719285, -1.14718983332966

H, 13.78254252219191, 7.07475844582879, -3.04125196986425

H, 13.48279299673825, 8.80251604318821, -3.40076577096832

H, 12.66621435230849, 7.53948756255053, -4.37052671766136

H, 11.60213767047737, -8.93703714169495, 0.01049487172708

H, 10.33503082337956, -9.56419903660409, -1.09851924194878

H, 10.86023995519804, -10.53840881775957, 0.30749204674164

H, -11.38689286204462, 8.86412752981470, -2.71732358905193

H, -12.61409208152863, 8.36475819010335, -1.50476318067781

H, -13.13017263931099, 8.98004035823836, -3.10351220600793

H, -7.62607691338326, -10.62976263706876, -1.58255229202064

H, -8.74360188597509, -11.65201675020158, -0.62980875862251

H, -7.44784226885715, -10.73957731109407, 0.20111534438224

Br, 8.71245637485124, 0.74522494126938, 2.09164703677627

$PC^{\cdot+}/X^-$ for PC5

charge = 0, multiplicity = 2

C, -2.37137145026535, -1.48684984083480, -0.12544176276830

C, -0.98033287733365, -1.46920004880625, -0.11830427540521

C, -0.25347124701485, -0.27960305428619, -0.23744084336066

C, -0.95548764053799, 0.94215383051263, -0.38541443188034

C, -2.35444954068638, 0.92612493984527, -0.37239392166088

C, -3.07667879147096, -0.25387542643063, -0.22496490065005

C, 1.12386700742193, 2.12956699656026, -0.44619415584190

C, 1.82724184882334, 0.90567510726515, -0.29750023334234

C, 3.22420188342040, 0.93247923940067, -0.21187986598518

H, 3.75849757585937, -0.00331968463190, -0.05041514736999

C, 3.94125610656733, 2.12096466243441, -0.26845445107849

C, 3.23747271608094, 3.34589064934721, -0.46405280793913

C, 1.85045755725746, 3.32280284334875, -0.53551460630680

H, -0.43840806762645, -2.41178730590847, -0.04571371656356

H, -2.89128567499903, 1.87226841945192, -0.43600818539915

H, 1.31064258748849, 4.25607123205792, -0.69385557110109

N, -0.24844402009289, 2.11856029824309, -0.51044261091817

N, 1.12457145226175, -0.27234362377069, -0.21969652527559

C, 1.82111267617745, -1.47723464385753, 0.12239144011418

C, 2.02032662679425, -1.75632573840174, 1.47419425656240

C, 2.27535095562311, -2.32830492178643, -0.87937299715041

C, 2.68988627849506, -2.92262186240149, 1.82547329280327

H, 1.65186384308306, -1.03891847769708, 2.21889806401097

C, 2.94588073869962, -3.49473454430252, -0.52044954584945

H, 2.10133692794028, -2.07984237696713, -1.92803762835315

C, 3.14954677042828, -3.78740907583099, 0.82799030674300

H, 2.85346972817076, -3.16046243795461, 2.87888236104769

H, 3.30591275768374, -4.17540148509721, -1.29372531797467

C, -0.95526028435988, 3.36443287260062, -0.46448909303068

C, -1.40425670721931, 3.95466119958013, -1.64039855849294

C, -1.17168732476935, 3.94820940617003, 0.78394548030780

C, -2.09298409032746, 5.16339364772973, -1.56856888625511

H, -1.21774915057567, 3.47089298911789, -2.60109874297929

C, -1.85809657234061, 5.15451416062068, 0.84829905076908

H, -0.78786076758646, 3.43650534051548, 1.67634661645985

C, -2.31687635273141, 5.75710336001659, -0.32734168039672

H, -2.45436905377849, 5.63889115458866, -2.48168744816581

H, -2.03990800662762, 5.62694086991547, 1.81654736509501

C, 3.90739556044927, -5.02281851821726, 1.22453006019617

C, -3.03448858441705, 7.07346510765954, -0.23055590235617

F, -3.62333082747219, 7.41876764646092, -1.37796162557155

F, -2.19732851589535, 8.06856424709246, 0.09597318525096

F, -3.98472808029028, 7.04948930692086, 0.71247864995062

F, 3.92304238339078, -5.94127353273087, 0.25454611262528

F, 5.18618073048272, -4.74485236427556, 1.51780550801522

F, 3.38187792999513, -5.60048656674457, 2.31075310181432

B,r 0.69289267457622, 1.45458366401624, 2.82874429373570

C, 3.92750791947098, 4.64706051503199, -0.61628416627054

C, 4.99420003017392, 4.79685169577423, -1.48403047350263

C, 3.48251304482122, 5.77229625609407, 0.13178508806384

C, 5.66012597392986, 6.03794638485850, -1.62455131995317

H, 5.34170065876535, 3.94962876334753, -2.07985987949803

C, 4.11123067434189, 6.98895412651907, 0.01829267250213

H, 2.64333303868518, 5.65438902486655, 0.82196385982937

C, 6.76536269004272, 6.19870362103556, -2.50282938566182

C, 5.21683489114981, 7.15927080989597, -0.85488854025937

H, 3.76911171123957, 7.84237915547398, 0.60983325551329

C, 7.40356963783599, 7.41276109423220, -2.61033732549290

H, 7.10123844273763, 5.33809879696780, -3.08776860270622

C, 5.89589249573574, 8.39814604320193, -0.98914492425059

C, 6.96496826587309, 8.52209027870105, -1.84744736425266

H, 8.25468132464196, 7.52571358715536, -3.28672687165847

H, 5.55447882263689, 9.25239549492844, -0.39810736890200

H, 7.48117939154036, 9.48072938793758, -1.94364302530135

C, -3.05679406518989, -2.79775381515056, -0.05547270113574

C, -4.07587945819086, -3.12067372804433, -0.93317811396259

C, -2.65386972615978, -3.75507968409397, 0.91644513214697

C, -4.73523504779138, -4.37144859724293, -0.86813693768289

H, -4.39008246136372, -2.40676027977821, -1.69806254289834

C, -3.27672082534207, -4.97688981609036, 1.00485275478111

H, -1.85151157528816, -3.50066801806632, 1.61344936757331

C, -5.79242702834410, -4.70694573462076, -1.75627312894572

C, -4.33465855708033, -5.32104814737830, 0.12375174541971

H, -2.96669670634978, -5.69859151730049, 1.76528065521524

C, -6.42479335689834, -5.92529132948944, -1.66346893591166

H, -6.09624182443036, -3.97677133220148, -2.51129270767849

C, -5.00650978924431, -6.56916301309245, 0.19288386367375

C, -6.02849962289391, -6.86449101563087, -0.68077743209920

H, -7.23899886234797, -6.17306230857721, -2.34927399425076

H, -4.69757249634598, -7.29172702413715, 0.95319656389940

H, -6.53988616744727, -7.82851524740598, -0.61869463021605

C, -4.55266134764274, -0.16682119534059, -0.13793712859328

C, -5.24758205433764, -0.81540562943446, 0.86691381976414

C, -5.27462689545419, 0.61279937846618, -1.08354240382654

C, -6.65763276925674, -0.73342852337977, 0.95874068286776

H, -4.71133910639248, -1.40525839238441, 1.61393323866519

C, -6.64414472171006, 0.70927470744133, -1.02100019693530

H, -4.72884070626165, 1.12352386391192, -1.88079882201597

C, -7.38108256095792, -1.40576532635710, 1.98024756987923

C, -7.37636900216295, 0.03996627085518, -0.00631716674406

H, -7.18836423424966, 1.30124542318921, -1.76172283247438

C, -8.75273041439242, -1.31767804322449, 2.04061691942851

H, -6.82695788954159, -1.99692937616461, 2.71444849166982

C, -8.79091080946749, 0.11124026163721, 0.08379481017696

C, -9.46371583249914, -0.55250831249321, 1.08460456664902

H, -9.30067927607590, -1.84042175679388, 2.82873586498853

H, -9.33639256819062, 0.70289057711874, -0.65648275681110

H, -10.55336001658658, -0.49146028289564, 1.14512239043535

C, 5.40938427197933, 2.06457345357224, -0.08507371680996

C, 6.05115831853211, 2.90844696333444, 0.80352310469563

C, 6.17867864594972, 1.11501657876055, -0.81294037425680

C, 7.45402229292749, 2.85822630057049, 0.98451116486345

H, 5.47733451484056, 3.63256561592183, 1.38646090256534

C, 7.54261715297819, 1.04470093072295, -0.66004197848483

H, 5.67440896872804, 0.44920515213779, -1.51770150957100

C, 8.12361842410626, 3.72984153943575, 1.88489099550771

C, 8.22134432099904, 1.91201806663590, 0.23488882101633

H, 8.12401516574114, 0.31962739194398, -1.23584586608922

C, 9.49012946385911, 3.66870750975075, 2.03260819336014

H, 7.53259100464120, 4.45238152192621, 2.45431024035208

C, 9.62901362785750, 1.87344115449022, 0.41087868241171

C, 10.24931029133083, 2.73255886722886, 1.28955653963606

H, 9.99650011934415, 4.34537980777229, 2.72569885072990

H, 10.21185606735783, 1.14905448568399, -0.16461211224724

H, 11.33419542234129, 2.69516592770046, 1.41737372299118

References

(1) Tossell, J. Calculation of the Properties of Molecules in the Pyridine Catalyst System for the Photochemical Conversion of CO_2 to Methanol. *Comput. Theor. Chem.* **2011**, *977*, 123–127.

(2) Theriot, J. C.; Lim, C. H.; Yang, H.; Ryan, M. D.; Musgrave, C. B.; Miyake, G. M. Organocatalyzed Atom Transfer Radical Polymerization Driven by Visible Light. *Science* **2016**, *352*, 1082–1086.

(3) Schäfer, A.; Horn, H.; Ahlrichs, R. Fully Optimized Contracted Gaussian Basis Sets for Atoms Li to Kr. *J. Chem. Phys.* **1992**, *97*, 2571–2577.

(4) Marenich, A. V.; Cramer, C. J.; Truhlar, D. G. Universal Solvation Model Based on Solute Electron Density and on a Continuum Model of the Solvent Defined by the Bulk Dielectric Constant and Atomic Surface Tensions. *J. Phys. Chem. B* **2009**, *113*, 6378–6396.

(5) Becke, A. D. Density-Functional Thermochemistry. III. The Role of Exact Exchange. *J. Chem. Phys.* **1993**, *98*, 5648–5652.

(6) Stephens, P. J.; Devlin, F. J.; Chabalowski, C. F.; Frisch, M. J. Ab Initio Calculation of Vibrational Absorption and Circular Dichroism Spectra using Density Functional Force Fields. *J. Phys. Chem.* **1994**, *98*, 11623–11627.

(7) Grimme, S.; Ehrlich, S.; Goerigk, L. Effect of the Damping Function in Dispersion Corrected Density Functional Theory. *J. Comput. Chem.* **2011**, *32*, 1456–1465.

(8) Adamo, C.; Barone, V. Toward Reliable Density Functional Methods without Adjustable Parameters: The PBE0 Model. *J. Chem. Phys.* **1999**, *110*, 6158–6170.

(9) Zhao, Y.; Truhlar, D. G. The M06 Suite of Density Functionals for Main Group Thermochemistry, Thermochemical Kinetics, Noncovalent Interactions, Excited States, and

Transition Elements: Two New Functionals and Systematic Testing of Four M06-Class Functionals and 12 Other Functionals. *Theor. Chem. Acc.* **2008**, *120*, 215–241.

(10) Grimme, S.; Antony, J.; Ehrlich, S.; Krieg, H. A Consistent and Accurate Ab Initio Parametrization of Density Functional Dispersion Correction (DFT-D) for the 94 Elements H-Pu. *J. Chem. Phys.* **2010**, *132*, 154104.

(11) Y., T.; T., D. P.; H., N. C. A New Hybrid Exchange-Correlation Functional Using the Coulomb-Attenuating Method (CAM-B3LYP). *Chem. Phys. Lett.* **2004**, *393*, 51–57.

(12) Lin, Y.; Li, G.; Mao, S.; Chai, J. Long-Range Corrected Hybrid Density Functionals with Improved Dispersion Corrections. *J. Chem. Theory Comput.* **2013**, *9*, 263–272.

(13) Pan, X.; Fang, C.; Fantin, M.; Malhotra, N.; So, W. Y.; Peteanu, L. A.; Isse, A. A.; Gennaro, A.; Liu, P.; Matyjaszewski, K. Mechanism of Photoinduced Metal-Free Atom Transfer Radical Polymerization: Experimental and Computational Studies. *J. Am. Chem. Soc.* **2016**, *138*, 2411–2425.

(14) Miyake, G. M.; Theriot, J. C. Perylene as an Organic Photocatalyst for the Radical Polymerization of Functionalized Vinyl Monomers through Oxidative Quenching with Alkyl Bromides and Visible Light. *Macromolecules* **2014**, *47*, 8255–8261.

(15) Singh, V. K. et al. Highly Efficient Organic Photocatalysts Discovered via a Computer-Aided-Design Strategy for Visible-Light-Driven Atom Transfer Radical Polymerization. *Nat. Catal.* **2018**, *1*, 794ÍC804.

(16) Polgar, A. M.; Huang, S. H.; Hudson, Z. M. Donor modification of thermally activated delayed fluorescence photosensitizers for organocatalyzed atom transfer radical polymerization. *Polym. Chem.* **2022**, *13*, 3892–3903.

(17) Cole, J. P.; Federico, C. R.; Lim, C. H.; Miyake, G. M. Photoinduced Organocatalyzed

Atom Transfer Radical Polymerization Using Low ppm Catalyst Loading. *Macromolecules* **2019**, *52*, 747–754.

(18) Liu, W.; Xiao, Y. Relativistic time-dependent density functional theories. *Chem. Soc. Rev.* **2018**, *47*, 4481–4509.

(19) Lu, T.; Chen, F. Multiwfn: A Multifunctional Wavefunction Analyzer. *J. Comput. Chem.* **2012**, *33*, 580–592.

(20) Humphrey, W.; Dalke, A.; Schulten, K. VMD: Visual Molecular Dynamics. *J. Mol. Graph.* **1996**, *14*, 33–38.

(21) Li, Z.; Li, H.; Suo, B.; Liu, W. Localization of Molecular Orbitals: from Fragments to Molecule. *Acc. Chem. Res.* **2014**, *47*, 2758–2767.

(22) Wu, F.; Liu, W.; Zhang, Y.; Li, Z. Linear-Scaling Time-Dependent Density Functional Theory Based on the Idea of "From Fragments to Molecule". *J. Chem. Theory Comput.* **2011**, *7*, 3643–3660.

(23) Li, H.; Liu, W.; Suo, B. Localization of Open-Shell Molecular Orbitals via Least Change from Fragments to Molecule. *J. Chem. Phys.* **2017**, *146*, 104104.

(24) Liu, W.; Hong, G.; Dai, D.; Li, L.; Dolg, M. The Beijing Four-Component Density Functional Program Package (BDF) and Its Application to EuO, EuS, YbO and YbS. *Theoret. Chem. Acc.* **1997**, *96*, 75–83.

(25) Zhang, Y. et al. BDF: A Relativistic Electronic Structure Program Package. *J. Chem. Phys.* **2020**, *152*, 064113.

(26) Treat, N. J.; Sprafke, H.; Kramer, J. W.; Clark, P. G.; Barton, B. E.; Read de Alaniz, J.; Fors, B. P.; Hawker, C. J. Metal-Free Atom Transfer Radical Polymerization. *J. Am. Chem. Soc.* **2014**, *136*, 16096–16101.

(27) Corbin, D. A.; Puffer, K. O.; Chism, K. A.; Cole, J. P.; Theriot, J. C.; McCarthy, B. G.; Buss, B. L.; Lim, C.; Lincoln, S. R.; Newell, B. S.; Miyake, G. M. Radical Addition to N,N-Diaryl Dihydrophenazine Photoredox Catalysts and Implications in Photoinduced Organocatalyzed Atom Transfer Radical Polymerization. *Macromolecules* **2021**, *54*, 4507–4516.

(28) Price, M. J.; Puffer, K. O.; Kudisch, M.; Knies, D.; Miyake, G. M. StructureÍCproperty Relationships of Core-substituted Diaryl Dihydrophenazine Organic Photoredox Catalysts and Their Application in O-ATRP. *Polym. Chem.* **2021**, *12*, 6110–6122.

(29) H., W.; Z., J.; H., X.; D., J.; F., Z.; Z., N.; G., K. Continuous Flow Photoinduced Phenothiazine Derivatives Catalyzed Atom Transfer Radical Polymerization. *Eur. Polym. J.* **2020**, *126*, 109565.

(30) Pearson, R. M.; Lim, C. H.; McCarthy, B. G.; Musgrave, C. B.; Miyake, G. M. Organocatalyzed Atom Transfer Radical Polymerization Using N-Aryl Phenoxazines as Photoredox Catalysts. *J. Am. Chem. Soc.* **2016**, *138*, 11399–11407.

(31) McCarthy, B. G.; Pearson, R. M.; Lim, C. H.; Sartor, S. M.; Damrauer, N. H.; Miyake, G. M. Structure-Property Relationships for Tailoring Phenoxazines as Reducing Photoredox Catalysts. *J. Am. Chem. Soc.* **2018**, *140*, 5088–5101.

(32) Li, Z.; Suo, B.; Zhang, Y.; Xiao, Y.; Liu, W. Combining Spin-adapted Open-shell TD-DFT with SpinÍCOrbit Coupling. *Mol. Phys.* **2013**, *111*, 3741–3755.
